# Effects of different intervention modalities combined with exercise in patients with insomnia: a systematic review and network meta-analysis

**DOI:** 10.3389/fpubh.2026.1873034

**Published:** 2026-06-18

**Authors:** Xiangyang Zhou, Lei Chen, Wenhao Chen, Yanyan Zhou, Ziqi Li, Aichun Li, Junlai Zhou

**Affiliations:** 1 School of Physical Education, Hainan Normal University, Haikou, Hainan, China; 2 School of Agriculture and Environment, Faculty of Science, The University of Western Australia, Perth, WA, Australia

**Keywords:** combined interventions, exercise interventions, insomnia, network meta-analysis, sleep quality

## Abstract

**Background:**

Exercise, as a non-pharmacological intervention, is a promising adjunct for improving sleep outcomes in insomnia. However, a comprehensive assessment of exercise-based combination interventions remains insufficient. This study aimed to systematically evaluate and rank the intervention effects of different modalities combined with exercise on sleep outcomes via network meta-analysis (NMA).

**Methods:**

Following the population, intervention, comparator, outcome, and study design (PICOS) framework, literature was searched in PubMed, Web of Science, Cochrane Library, Embase, CNKI, and CQVIP for randomized controlled trials (RCTs) until March 20, 2026. Independent screening and data extraction were conducted. NMA utilized a frequentist framework, with risk of bias assessed by the Cochrane RoB 2 tool and evidence certainty assessed by CINeMA.

**Results:**

A total of 31 RCTs involving 2,457 participants were included, evaluating five types of exercise-based combination interventions. Results based on the surface under the cumulative ranking curve (SUCRA) indicated: (1) Pittsburgh Sleep Quality Index (PSQI): exercise plus cognitive behavioral therapy (EX + CBT) > qigong plus CBT (QG + CBT) > exercise plus traditional Chinese medicine interventions (EX + TCM) > qigong plus TCM interventions (QG + TCM) > exercise (EX) > TCM > conventional control (CON) > qigong (QG); (2) sleep onset latency (SOL): EX + CBT > QG + TCM > QG plus active repetitive transcranial magnetic stimulation (QG + ArTMS) > QG + CBT > EX > CON > EX + TCM > QG plus sham repetitive transcranial magnetic stimulation (QG + SrTMS); (3) total sleep time (TST): EX + TCM > EX > QG + SrTMS > QG + ArTMS > EX + CBT > CON > QG + TCM > QG + CBT; (4) wake after sleep onset (WASO): EX + CBT > QG + ArTMS > QG + SrTMS > CON > EX > EX + TCM. Compared with CON, EX + CBT showed significant effects on PSQI, SOL, and WASO, whereas no intervention significantly increased TST.

**Conclusions:**

Current evidence suggests that exercise-based combination interventions may improve subjective sleep quality and selected sleep-continuity parameters in insomnia. Of these, EX + CBT appears to be the most consistently supported combination, with advantages for PSQI, SOL, and WASO. However, evidence certainty was generally limited. For other exercise-based combination interventions, further validation in large, high-quality trials is warranted.

**Systematic review registration:**

PROSPERO, https://www.crd.york.ac.uk/PROSPERO/view/CRD420251125784, identifier CRD420251125784.

## 1 Introduction

In recent years, alongside the accelerating pace of modern life, insomnia has become an increasingly prominent public health concern. Data indicate that more than 800 million individuals worldwide are affected by insomnia, and the global prevalence of insomnia disorder has surpassed 16%, representing a substantial and growing public health burden (1–3). As research on insomnia disorder has advanced, pharmacological therapies, psychological and behavioral interventions, and physical treatment modalities have been widely incorporated into clinical practice and have shown benefits in alleviating insomnia symptoms (4–7). Nevertheless, these approaches remain constrained by suboptimal treatment adherence, limited long-term efficacy, and treatment costs (8, 9). Therefore, identifying safe, effective, and cost-effective intervention strategies for insomnia remains a priority in both clinical practice and public health.

Exercise has emerged as a promising non-pharmacological strategy for alleviating insomnia symptoms, given its favorable safety profile, feasibility, cost-efficiency, and broad accessibility (10, 11). Memon et al. (12) suggested that exercise may indirectly improve sleep by modulating neurotransmitter levels, attenuating immune-inflammatory responses, and alleviating negative emotional states such as depression and anxiety. Korkutata et al. (13) further indicated that exercise may promote sleep through multiple pathways, including regulation of circadian rhythms, modulation of melatonin secretion, and improvement of sleep architecture. Beyond subjective sleep symptoms, poor sleep quality has also been associated with cognitive and functional impairments, including slower reaction time and reduced psychomotor performance in physically active populations (14, 15). Meanwhile, with the continued development of related research, exercise interventions have increasingly been integrated with other therapeutic modalities, potentially yielding additive or synergistic effects that further enhance sleep quality and serve as an important adjunct to conventional interventions (16–18).

Recent European and Brazilian clinical guidelines on insomnia, together with Australian 24-h movement guidelines, collectively support the role of exercise and physical activity in improving sleep and promoting sleep health. The 2023 European Insomnia Guideline recommends incorporating exercise into the psychological and behavioral treatment framework for insomnia as an adjunct to cognitive behavioral therapy (19). The 2023 Brazilian Guidelines for the Diagnosis and Treatment of Insomnia explicitly recommend aerobic exercise as an adjuvant therapy for insomnia (20). The Australian 24-h Movement Guidelines for Adults and Older Adults provide specific physical activity recommendations, advising adults to engage regularly in moderate-to-vigorous physical activity and to perform muscle-strengthening activities on at least 2 days per week to promote sleep quality (21). However, existing studies have not adequately compared the potential benefits of combining exercise with different intervention modalities. Published meta-analyses and network meta-analyses have mainly focused on conventional interventions or stand-alone exercise interventions, leaving the additive or synergistic effects of exercise combined with different intervention strategies insufficiently explored, with no consistent conclusions established (22).

Conventional pairwise meta-analysis relies primarily on direct comparative evidence and is therefore limited in its ability to comprehensively compare and rank the relative effects of multiple exercise-based combination interventions within a unified analytical framework (23, 24). Accordingly, we conducted a network meta-analysis to systematically compare the effects of exercise combined with different intervention modalities on sleep outcomes in patients with insomnia, aiming to identify the potentially optimal combination intervention for improving insomnia symptoms and to provide evidence for intervention selection and practical application.

## 2 Materials and Methods

This study was conducted and reported in accordance with the Preferred Reporting Items for Systematic Reviews and Meta-Analyses 2020 statement (PRISMA 2020) and the PRISMA extension for network meta-analyses (PRISMA-NMA) (25, 26). The protocol was prospectively registered with the International Prospective Register of Systematic Reviews (PROSPERO; registration number: CRD420251125784).

### 2.1 Data sources and search strategy

Guided by the population, intervention, comparator, outcome, and study design (PICOS) framework, two investigators (X.Y.Z. and W.H.C.) independently searched PubMed, Web of Science, the Cochrane Library, Embase, China National Knowledge Infrastructure (CNKI), and the VIP Chinese Journal Database (CQVIP) from database inception to March 20, 2026. The literature search was completed on May 20, 2026. Search strategies were developed using a combination of subject headings and free-text terms related to insomnia, exercise, combined interventions, and randomized controlled trials. The complete search strategies are provided in Supplementary Table 1.

### 2.2 Eligibility criteria

Eligible studies were required to satisfy all of the following criteria: (1) Participants were patients with insomnia disorder diagnosed according to the Diagnostic and Statistical Manual of Mental Disorders, Fifth Edition (DSM-5), the International Classification of Sleep Disorders, Third Edition (ICSD-3), the International Classification of Diseases (ICD), or other recognized diagnostic criteria; participants without a formally reported clinical diagnosis were also eligible if insomnia symptoms were assessed using a validated standardized insomnia assessment instrument (27, 28) and met the diagnostic cut-off prespecified in the original study. (2) The experimental group received exercise combined with at least one non-exercise intervention. (3) Comparator interventions included routine care, exercise alone, or another single treatment modality. (4) At least one extractable sleep-related outcome was reported. (5) The study was a randomized controlled trial (RCT). Studies were excluded if they met any of the following criteria: (1) Non-RCT design. (2) Participants were not patients with insomnia or the diagnostic criteria were insufficiently described. (3) The intervention did not meet the inclusion criteria. (4) The full text was unavailable. (5) Outcome data were incomplete or could not be extracted. (6) Duplicate publications, in which case only the report with the most complete information was retained.

### 2.3 Study selection, data extraction, and intervention coding

All retrieved records were imported into EndNote 2025 for reference management and deduplication. Two investigators (X.Y.Z. and W.H.C.) independently screened titles and abstracts, followed by full-text assessment of potentially eligible studies. Disagreements were resolved through discussion and, when necessary, adjudicated by a third investigator (A.C.L.). For eligible studies, two investigators independently extracted data using a prespecified standardized data extraction form. Extracted information covered four domains: (1) study characteristics, including first author, publication year, and country; (2) participant characteristics, including sample size, age, diagnostic criteria or insomnia assessment instrument, and baseline insomnia severity; (3) intervention characteristics, including intervention type, session duration, weekly frequency, and intervention duration; and (4) outcome data, including pre- and post-intervention means, standard deviations, and sample sizes for each group. The primary outcome was the total score of the Pittsburgh Sleep Quality Index (PSQI). Secondary outcomes included total sleep time (TST), sleep onset latency (SOL), and wake after sleep onset (WASO). For multi-arm trials, data from each intervention arm were extracted separately. Intervention nodes were coded according to the core intervention components of each study arm. Traditional Chinese mind–body exercises were assigned to the qigong (QG) node, rather than the general exercise therapy node (EX), because they integrate movement, breathing regulation, and attentional regulation as core features of mind–body practice (29, 30). In this review, exercise was used broadly to include both conventional exercise therapy and traditional Chinese mind–body exercises, while EX and QG were analyzed as separate nodes because of their distinct intervention characteristics. The operational definitions of all intervention nodes are provided in Supplementary Table 2.

### 2.4 Risk of bias assessment and certainty of evidence assessment

The risk of bias in the included studies was independently assessed by two investigators (X.Y.Z. and W.H.C.) using the revised Cochrane risk-of-bias tool for randomized trials (RoB 2) (31). RoB 2 assessments were conducted at the outcome level and covered five domains: bias arising from the randomization process, bias due to deviations from intended interventions, bias due to missing outcome data, bias in measurement of the outcome, and bias in selection of the reported result. Each domain, together with the overall risk of bias, was judged as low risk, some concerns, or high risk. If disagreements arose, they were resolved through discussion and, when necessary, adjudicated by a third investigator (J.L.Z.).

The certainty of evidence for the network meta-analysis (NMA) estimates was assessed using the Confidence in Network Meta-Analysis (CINeMA) framework (32, 33), which covers six domains: within-study bias, reporting bias, indirectness, imprecision, heterogeneity, and incoherence. The results of the RoB 2 assessment were incorporated into the within-study bias domain. Imprecision was judged against clinically important effect thresholds prespecified for this review within the CINeMA assessment. The certainty of evidence for each pairwise treatment comparison within each outcome network was ultimately rated as high, moderate, low, or very low.

### 2.5 Statistical analysis

Statistical analyses were performed using R software (version 4.5.2). NMA was conducted within a frequentist framework using the netmeta package (34). Continuous outcomes were summarized as mean differences (MDs) with 95% confidence intervals (CIs). The NMA was implemented using a graph-theoretical approach (35). Overall network heterogeneity was assessed using *I*^2^ and Q statistics. Both common-effect and random-effects models were fitted. Given the anticipated clinical and methodological heterogeneity across included studies in terms of population characteristics, intervention modalities, and outcome measurement, the random-effects model was used as the primary model for interpretation, whereas the common-effect model was used as a supplementary analysis (36). Network plots were constructed to illustrate the pattern of direct comparisons across all intervention nodes. Global inconsistency was assessed using the design-by-treatment interaction model (37, 38). Local inconsistency was assessed using the node-splitting approach (Separating indirect from direct evidence, SIDE) to compare direct and indirect evidence (39). A two-sided *P*-value < 0.05 was considered to indicate statistically significant inconsistency. In addition, design contribution plots and net heat plots were used as complementary tools to identify designs and comparison pathways that may have contributed to network inconsistency (40, 41). Intervention ranking was expressed as the surface under the cumulative ranking curve (SUCRA), with higher SUCRA values indicating a more favorable relative ranking (42). Ranking results were interpreted together with effect estimates and their 95% CIs rather than being used as the sole basis for determining intervention superiority. Potential small-study effects and publication bias were assessed using comparison-adjusted funnel plots in combination with Egger's test (43, 44).

### 2.6 Network meta-regression analysis

To explore potential sources of heterogeneity, univariable network meta-regression analyses were performed for each prespecified potential effect modifier in turn (45). The covariates included comorbidity status, geographic region classified by continent, diagnostic criteria or insomnia assessment instrument, intervention duration, publication year, and sample size category. Results of each network meta-regression were visualized using bubble plots. Because study-level network meta-regression is observational in nature and may be affected by ecological bias, and because the number of studies was limited within some covariate categories, these results were interpreted as exploratory, and no definitive conclusions were drawn from them (46).

## 3 Results

### 3.1 Literature search results

**Figure 1:**
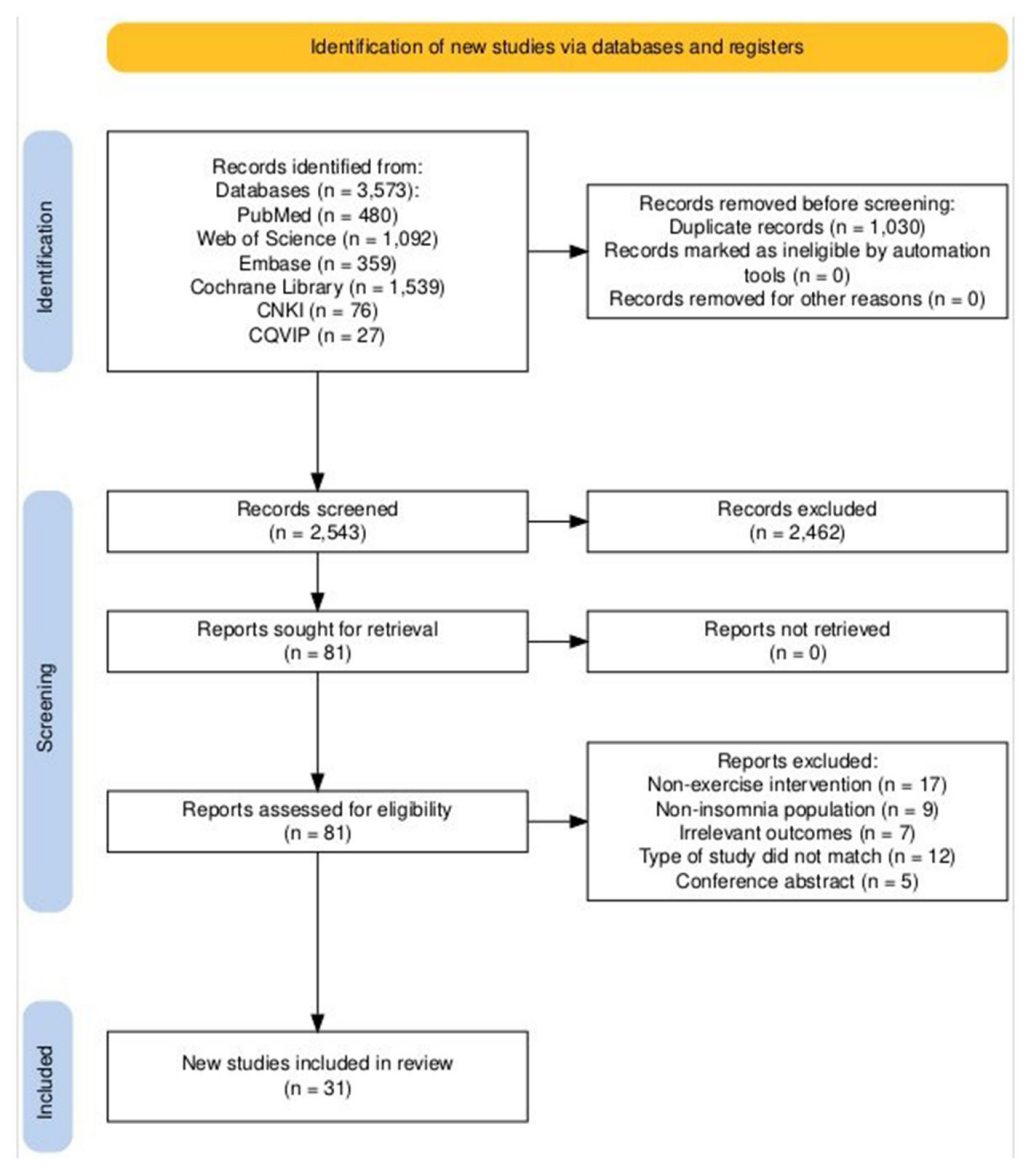
PRISMA flow diagram of the study selection process.

A systematic search of six databases yielded 3,573 records relevant to the research topic. After deduplication using EndNote 2025, 1,030 duplicate records were removed. The remaining 2,543 records were screened by title and abstract, of which 2,462 were excluded. Subsequently, 81 articles underwent full-text review, and 31 RCTs were ultimately included. The detailed study selection process is shown in Figure 1 and the reasons for exclusion after full-text assessment are presented in Supplementary Table 3.

### 3.2 Characteristics and risk of bias of included studies

A total of 31 RCTs involving 2,457 patients with insomnia were included, evaluating five types of exercise-based combination interventions (47–77). The number of studies for each intervention category was as follows: qigong-based traditional exercise combined with traditional Chinese medicine therapy (QG + TCM), 12 studies (48, 52–54, 56, 59, 62, 68, 69, 72, 73, 76) exercise therapy combined with cognitive behavioral therapy (EX + CBT), 9 studies (47, 49, 50, 57, 60, 61, 64, 71, 74) qigong-based traditional exercise combined with cognitive behavioral therapy (QG + CBT), 5 studies (51, 63, 65–67) exercise therapy combined with traditional Chinese medicine therapy (EX + TCM), 4 studies (55, 58, 70, 75) and qigong-based traditional exercise combined with repetitive transcranial magnetic stimulation (QG + rTMS), 1 study (77). The one QG + rTMS trial included both active stimulation (QG + ArTMS) and sham stimulation (QG + SrTMS) arms, which were treated as separate intervention nodes in the network analysis. The baseline characteristics of the included studies are presented in Table 1.

**Table 1:** Characteristics of the included studies.

Study	Country	Age	Sample size		Diagnostic standards	Intervention		Intervention period	Outcome	Quality assessment
			E	C		E	C			
Li (76)	China	56.93 ± 3.89	30	30	Clinical diagnosis	QG + TCM	CON	8 weeks	②③	S
Høeg et al. (47)	Denmark	71–81	5	7	ISI	EX + CBT	CON	12 weeks	①②③④	H
Li (48)	China	46.17 ± 2.16	40	C1:40 C2:40	Clinical diagnosis	QG + TCM	C1:QG C2:TCM	8 weeks	①	S
Qiu (49)	China	70.14 ± 3.65	60	60	Chinese guideline	EX + CBT	EX	4 weeks	①②③	S
Wu (50)	China	65.95 ± 4.35	39	39	PSQI	EX + CBT	EX	4 weeks	①	S
Xiong (51)	China	45.38 ± 7.98	63	63	Clinical diagnosis	QG + CBT	CON	4 weeks	①②③	S
Xiong (52)	China	NR	30	30	Chinese guideline	QG + TCM	CON	8 weeks	①	S
Han (53)	China	45.5 ± 10.7	32	C1:35 C2:30 C3:31	DSM-5	QG + TCM	C1:CON C2:QG C3:TCM	6 weeks	①	S
He (77)	China	T1: 66.63 ± 5.32 T2: 68.50 ± 4.97	T1:38 T2:38	38	PSQI	T1:QG + ArTMS T2:QG + SrTMS	EX	4 weeks	②③④	S
He (54)	China	51.30 ± 7.51	30	C1:38 C2:38	Clinical diagnosis	QG + TCM	C1:QG C2:TCM	4 weeks	①	S
Jiang (55)	China	40.00 ± 13.04	30	15	PSQI	EX + TCM	EX	8 weeks	①	S
Xiong (56)	China	70.24 ± 3.19	30	30	PSQI	QG + TCM	CON	E:4 weeks; C: 12 days	①	S
Cammalleri et al. (57)	Canada	50 ± 14	6	7	DSM-5	EX + CBT	EX	16 weeks	①②③④	H
Chen (58)	China	37.18 ± 7.28	11	C1:10 C2:13	Clinical diagnosis	EX + TCM	C1:EX C2:TCM	4 weeks	①	S
Chen (59)	China	62.05 ± 7.86	40	C1:40 C2:40	CCMD-3	QG + TCM	C1:QG C2:TCM	2 weeks	①	S
He (60)	China	36.21 ± 6.36	70	70	Clinical diagnosis	EX + CBT	CON	12 weeks	①	S
Liu (61)	China	50.69 ± 5.11	40	40	Clinical diagnosis	EX + CBT	CON	3 weeks	①	S
Min (62)	China	68.58 ± 3.41	40	40	Chinese guideline	QG + TCM	TCM	4 weeks	①	S
Ferreira et al. (75)	Brazil	43.2 ± 10.7	8	8	DSM-5	EX + TCM	EX	12 weeks	①②③④	H
Wang (63)	China	45.17 ± 5.74	36	36	Clinical diagnosis	QG + CBT	CON	12 weeks	①	S
Zhai (64)	China	37.68 ± 7.59	38	38	PSQI	EX + CBT	CON	4 weeks	①	S
Gao (65)	China	61.32 ± 12.42	50	50	PSQI	QG + CBT	CON	4 weeks	①	S
Yang (66)	China	67.83 ± 4.69	43	43	Clinical diagnosis	QG + CBT	CON	4 weeks	①	S
Wei (67)	China	66.76 ± 3.58	32	29	DSM-5	QG + CBT	EX + CBT	8 weeks	①	S
Zhang (68)	China	45.2 ± 10.7	73	73	CCMD-3	QG + TCM	TCM	4 weeks	①	S
Ren (69)	China	T1: 42.37 ± 5.99 T2: 38.57 ± 7.43	T1:30 T2:30	30	PSQI	T1:QG + TCMa T2:QG + TCMb	TCM	4 weeks	①	S
Zheng (70)	China	57.60 ± 5.20	45	45	CCMD-3	EX + TCM	EX	8 weeks	①	S
Song (71)	China	49.97 ± 4.62	50	50	CCMD	EX + CBT	EX	8 weeks	①	S
Wang (72)	China	62.32 ± 3.41	40	40	Clinical diagnosis	QG + TCM	CON	4 weeks	①	H
Zhao et al. (73)	China	21.14 ± 3.88	14	13	Clinical diagnosis	QG + TCM	TCM	4 weeks	①	S
Reid (74)	USA	62 ± 4.5	10	7	PSQI	EX + CBT	CON	16 weeks	①	H

① PSQI, ② SOL, ③ TST, ④ WASO. E, experimental group. C, comparator/control group. T1/T2, different treatment arms. C1/C2/C3, different comparator arms. EX, exercise; QG, qigong-based exercise; TCM, non-pharmacological traditional Chinese medicine therapy; CBT, cognitive behavioral therapy; CON, control; ArTMS, active rTMS; SrTMS, sham rTMS; rTMS, repetitive transcranial magnetic stimulation; TCMa and TCMb indicate Ziwu liuzhu nashi acupuncture and conventional acupuncture, respectively. PSQI, Pittsburgh Sleep Quality Index; ISI, Insomnia Severity Index; DSM-5, diagnostic and statistical manual of mental disorders, fifth edition; CCMD, Chinese classification of mental disorders; CCMD-3, Chinese classification of mental disorders, third edition; NR, not reported; S, some concerns; H, high risk of bias.

The detailed risk-of-bias assessment is presented in Supplementary Table 4. Among the 31 included studies, 26 were judged as having some concerns, and 5 were judged as having a high risk of bias (47, 57, 72, 74, 75). No study was rated as low risk of bias. Overall, the risk-of-bias profile of the included studies was suboptimal, with concerns mainly arising from the randomization process, deviations from intended interventions, missing outcome data, measurement of the outcome, and selection of the reported result. Therefore, the findings of this network meta-analysis should be interpreted with caution, particularly for comparisons supported by only a small number of trials, in which the risk of bias in individual studies may have a greater influence on the overall network estimates.

### 3.3 Network meta-analysis results

#### 3.3.1 Network evidence plots

**Figure 2:**
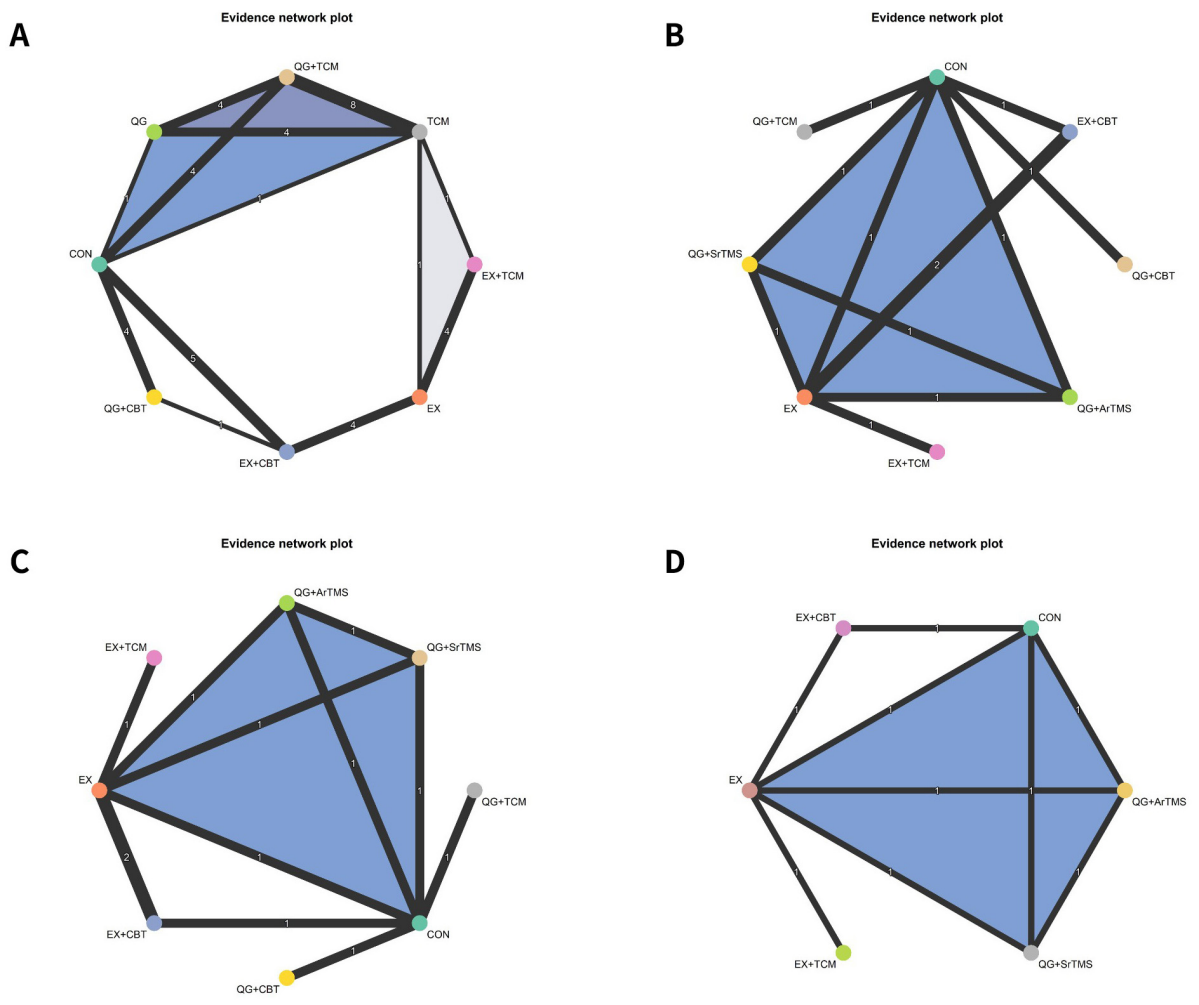
Network evidence plots for the four sleep outcomes: **(A)** PSQI, **(B)** SOL, **(C)** TST, and **(D)** WASO.

Network geometry varied across outcomes in the extent and distribution of direct evidence; however, all outcome-specific networks were connected, allowing subsequent indirect comparisons. For the primary outcome, PSQI, the network comprised 29 studies and 8 interventions, including 4 combination interventions (47–75), with 13 direct-comparison links. The most frequently studied comparison was QG + TCM vs. TCM, followed by CON vs. QG + CBT and EX vs. EX + CBT. For the secondary outcomes, the SOL network comprised 7 studies and eight interventions, including six combination interventions (47, 49, 51, 57, 75–77), with 11 direct-comparison links; EX vs. EX + CBT was the most frequently studied comparison (two studies). The TST network also comprised seven studies and eight interventions, including six combination interventions (47, 49, 51, 57, 75–77), with 11 direct-comparison links; EX vs. EX+CBT was the most frequently studied comparison (two studies). The WASO network comprised four studies and six interventions, including four combination interventions (47, 57, 75, 77), with nine direct-comparison links, each supported by only one direct comparison study. In the network plots, nodes represent different interventions, node size is proportional to the total sample size for each intervention, and line thickness reflects the number of studies contributing direct comparisons. The overall network evidence plots are shown in Figure 2, and individual outcome-specific network plots are provided in Supplementary Figures 1–4.

#### 3.3.2 Inconsistency assessment

Under the common-effect model, the global inconsistency test indicated significant inconsistency in the PSQI network (*Q* = 69.20, df = 7, *p* < 0.0001). Given the substantial heterogeneity in the network (*I*^2^ = 91.7%), the random-effects model was used as the primary basis for interpretation. Under the random-effects framework, global inconsistency was not statistically significant (*Q* = 12.33, df = 7, *p* = 0.0902). Further node-splitting analyses identified significant local inconsistency in three comparisons: EX + TCM vs. TCM, EX + CBT vs. QG + CBT, and QG + CBT vs. CON (*p* < 0.05, based on node-splitting). The corresponding inconsistency diagnostic plots are provided in Supplementary Figures 5–9. These comparisons should therefore be interpreted with caution.

#### 3.3.3 Comparative results for the primary outcome

Twenty-nine RCTs reported PSQI scores, involving 2,283 patients with insomnia and 8 interventions, of which 4 were combination interventions (47–75). The NMA results showed that, compared with conventional control (CON), EX + CBT (MD: −2.80, 95% CI: −4.15–−1.45), QG + CBT (MD: −2.73, 95% CI: −4.21–−1.24), EX + TCM (MD: −2.68, 95% CI: −5.02–−0.34), and QG + TCM (MD: −2.19, 95% CI: −3.60–−0.77) significantly reduced PSQI scores.

**Figure 3:**
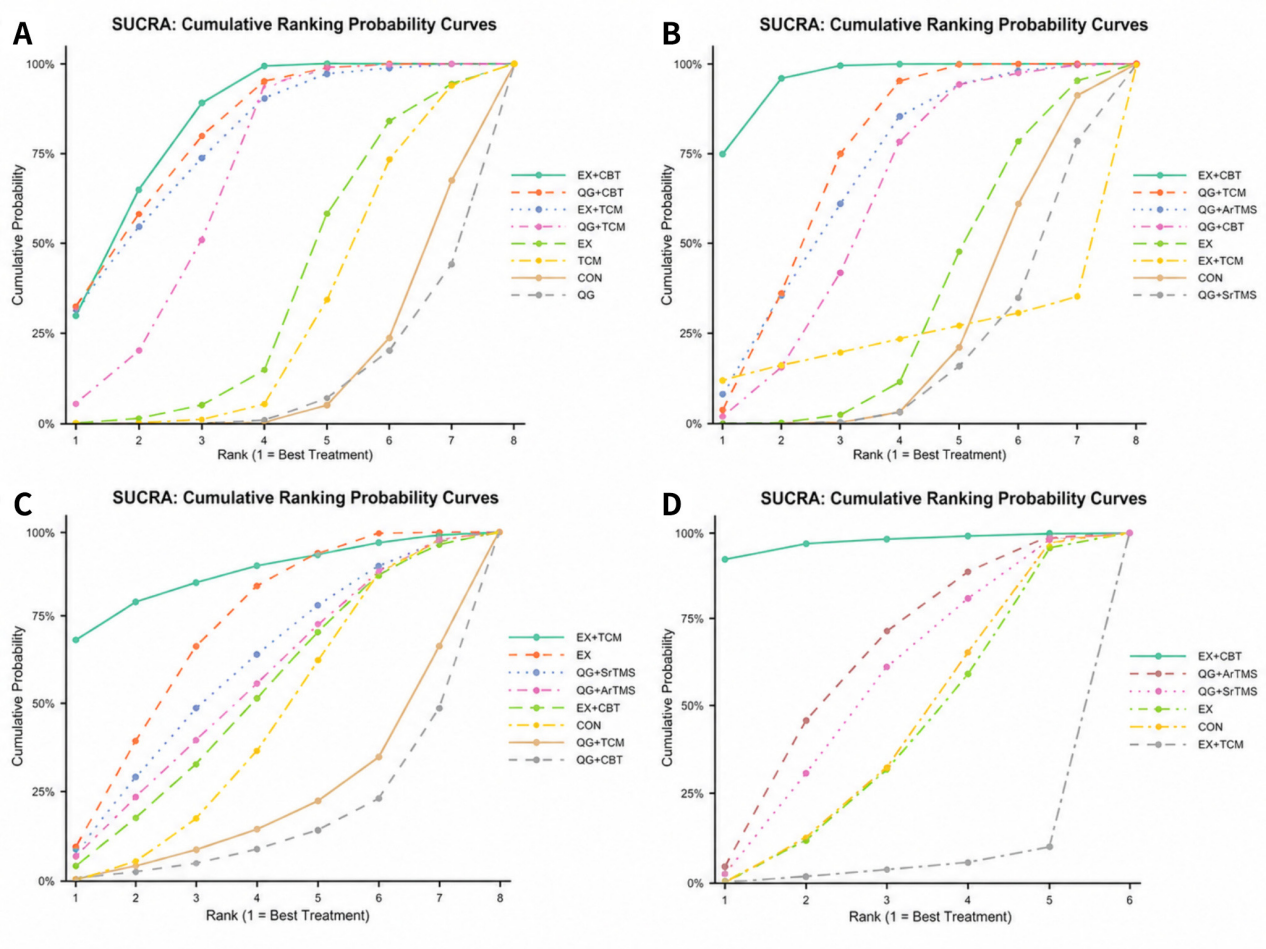
SUCRA ranking plots for the four sleep outcomes: **(A)** PSQI, **(B)** SOL, **(C)** TST, and **(D)** WASO.

Compared with EX alone, EX + CBT (MD: −1.92, 95% CI: −3.45–−0.38) and EX + TCM (MD: −1.79, 95% CI: −3.50–−0.09) showed greater effects. Compared with TCM alone, EX + CBT (MD: −2.20, 95% CI: −4.11–−0.29) and QG + TCM (MD: −1.59, 95% CI: −2.67–−0.50) were more effective. Compared with QG alone, EX + CBT (MD: −3.07, 95% CI: −5.24–−0.91), QG + CBT (MD: −3.00, 95% CI: −5.35–−0.64), EX + TCM (MD: −2.95, 95% CI: −5.62–−0.28), and QG + TCM (MD: −2.46, 95% CI: −3.89–−1.02) showed superior effects. No statistically significant differences were observed for the remaining comparisons. Notably, EX + CBT was superior to all single-component comparators. Detailed league tables and forest plots of the NMA estimates for PSQI are provided in Supplementary Table 5.1 and Supplementary Figures 10–13. The SUCRA rankings were as follows: EX + CBT (83.3%) > QG + CBT (80.7%) > EX + TCM (78.0%) > QG + TCM (67.0%) > EX (36.9%) > TCM (29.8%) > CON (13.8%) > QG (10.5%). The cumulative probability comparison is shown in Figure 3, and the detailed SUCRA ranking probabilities are presented in Supplementary Table 6.1.

#### 3.3.4 Comparative results for secondary outcomes

Seven RCTs reported TST, involving 461 patients with insomnia and eight interventions, of which six were combination interventions (47, 49, 51, 57, 75–77). The NMA results showed that none of the interventions differed significantly from conventional control (CON). No statistically significant differences were observed in any other pairwise comparisons. Detailed league tables and forest plots of the NMA estimates for TST are provided in Supplementary Table 5.2 and Supplementary Figures 14–15. The SUCRA rankings for TST were as follows: EX + TCM (86.2%) > EX (69.7%) > QG + SrTMS (58.7%) > QG + ArTMS (54.3%) > EX + CBT (51.0%) > CON (43.5%) > QG + TCM (21.7%) > QG + CBT (15.0%). Although EX + TCM and EX ranked relatively high, none of the pairwise comparisons reached statistical significance; therefore, these ranking results should be interpreted cautiously. The cumulative probability comparison is shown in Figure 3, and the detailed SUCRA ranking probabilities are presented in Supplementary Table 6.2.

Seven RCTs reported SOL, involving 461 patients with insomnia and eight interventions, of which six were combination interventions (47, 49, 51, 57, 75–77). The NMA results showed that, compared with conventional control (CON), EX + CBT (MD: −6.95, 95% CI: −10.79–−3.12), QG + TCM (MD: −4.03, 95% CI: −5.41–−2.65), and QG + CBT (MD: −3.00, 95% CI: −5.48–−0.52) significantly shortened SOL. QG + ArTMS, however, did not differ significantly from CON (MD: −3.83, 95% CI: −8.07–0.40). Compared with EX alone, EX + CBT significantly shortened SOL (MD: −6.33, 95% CI: −7.40–−5.26), whereas no statistically significant differences were observed between EX and the other interventions. Detailed league tables and forest plots of the NMA estimates for SOL are provided in Supplementary Table 5.3 and Supplementary Figures 16–17. The SUCRA rankings for SOL were as follows: EX + CBT (95.7%) > QG + TCM (72.8%) > QG + ArTMS (68.9%) > QG + CBT (61.3%) > EX (33.6%) > CON (25.2%) > EX + TCM (23.5%) > QG + SrTMS (19.0%). The cumulative probability comparison is shown in Figure 3, and the detailed SUCRA ranking probabilities are presented in Supplementary Table 6.3.

**Figure 4:**
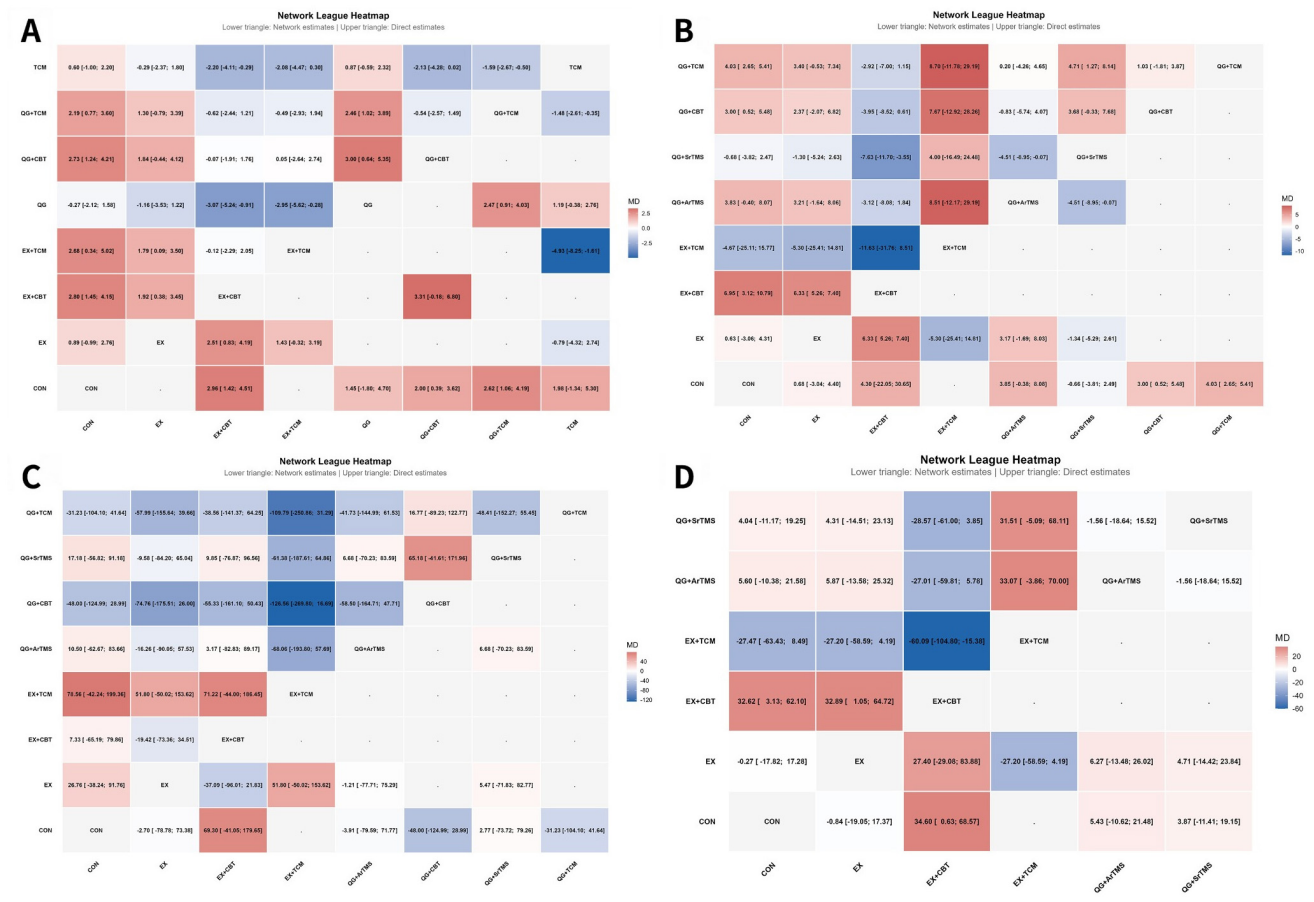
Network league heatmaps for the four sleep outcomes: **(A)** PSQI, **(B)** SOL, **(C)** TST, and **(D)** WASO.

Four RCTs reported WASO, involving 155 patients with insomnia and six interventions, of which four were combination interventions (47, 57, 75, 77). The NMA results showed that, compared with conventional control (CON), EX + CBT significantly shortened WASO (MD: −32.62, 95% CI: −62.10–−3.13). However, QG + ArTMS, QG + SrTMS, and EX + TCM did not differ significantly from CON. Compared with EX alone, EX + CBT significantly shortened WASO (MD: −32.89, 95% CI: −64.72–−1.05), whereas no statistically significant differences were observed between EX and the other interventions. Detailed league tables and forest plots of the NMA estimates for WASO are provided in Supplementary Table 5.4 and Supplementary Figures 18–19. The SUCRA rankings for WASO were as follows: EX + CBT (97.2%) > QG + ArTMS (61.9%) > QG + SrTMS (54.7%) > CON (41.9%) > EX (39.9%) > EX + TCM (4.4%). It should be noted that this outcome was supported by only a small number of studies (4 RCTs), and EX + TCM was supported by only one direct comparison study with wide confidence intervals. Therefore, the ranking results should be interpreted with caution. The cumulative probability comparison is shown in Figure 3, and the detailed SUCRA ranking probabilities are presented in Supplementary Table 6.4. Individual SUCRA ranking plots are provided in Supplementary Figures 20–23. The league heatmaps for PSQI, SOL, TST, and WASO are shown in Figure 4, with individual heatmaps provided in Supplementary Figures 24–27.

#### 3.3.5 Heterogeneity and network meta-regression analysis

**Figure 5:**
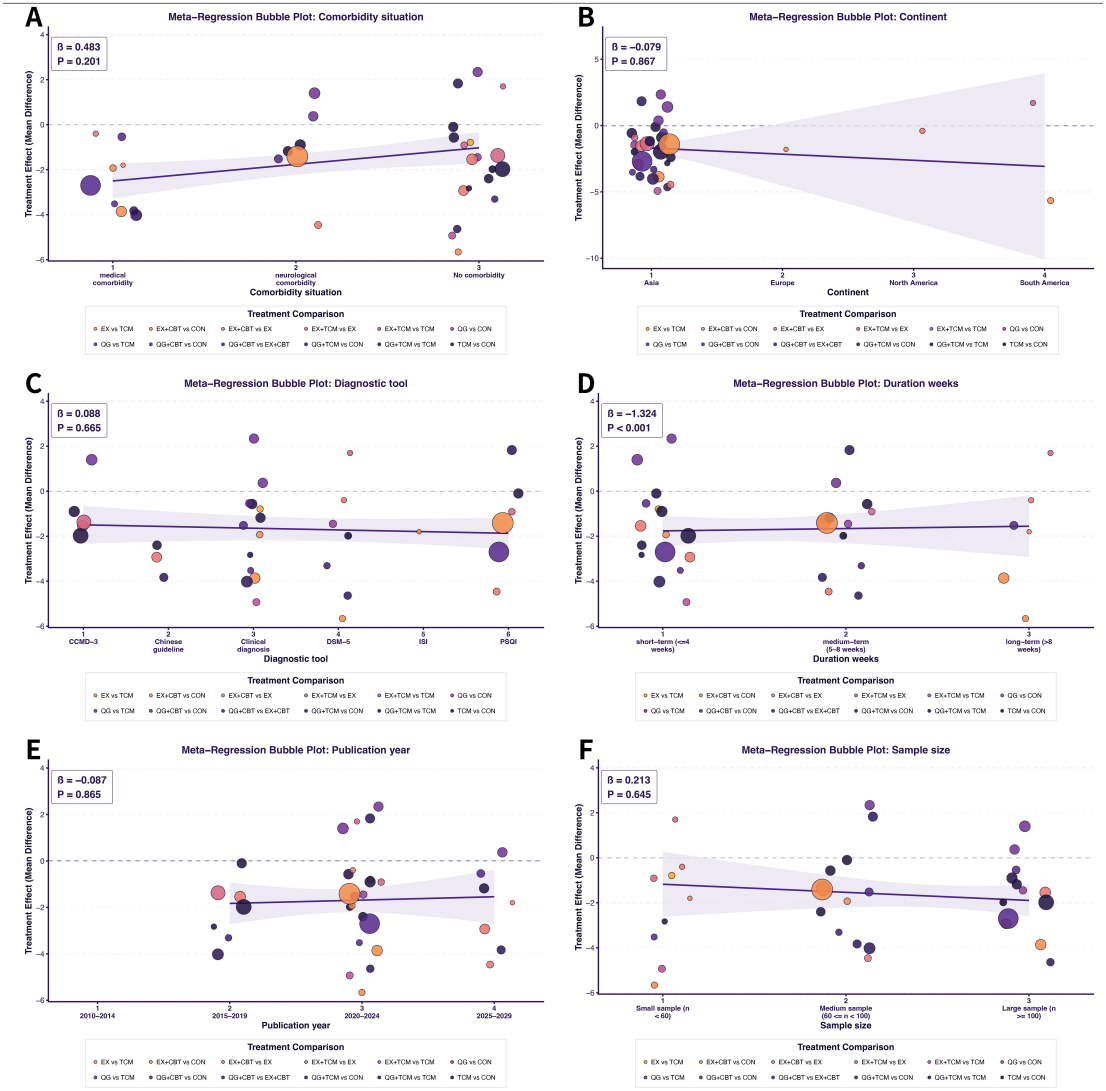
Network meta-regression bubble plots for Pittsburgh Sleep Quality Index (PSQI). The covariates shown are **(A)** comorbidity situation, **(B)** continent, **(C)** diagnostic tool, **(D)** duration in weeks, **(E)** publication year, and **(F)** sample size.

Heterogeneity was assessed using *I*^2^ and Q statistics. Substantial heterogeneity was observed across outcomes, particularly for the PSQI network (*I*^2^ = 91.7%). Further univariable network meta-regression showed that only intervention duration was significantly associated with PSQI treatment effects (β = −1.324, *p* < 0.001), suggesting that longer interventions were generally associated with greater improvement in PSQI scores (Figure 5). The full meta-regression estimates, including β, SE, z statistics, *P*-values, and 95% CIs for all covariates, are presented in Supplementary Table 7, and detailed bubble plots for each covariate are provided in Supplementary Figures 28–33. No significant moderating effects were identified for the remaining covariates. Nevertheless, these covariates did not fully explain the observed heterogeneity, and the direction of the main NMA findings was not substantially altered.

#### 3.3.6 Publication bias

**Figure 6:**
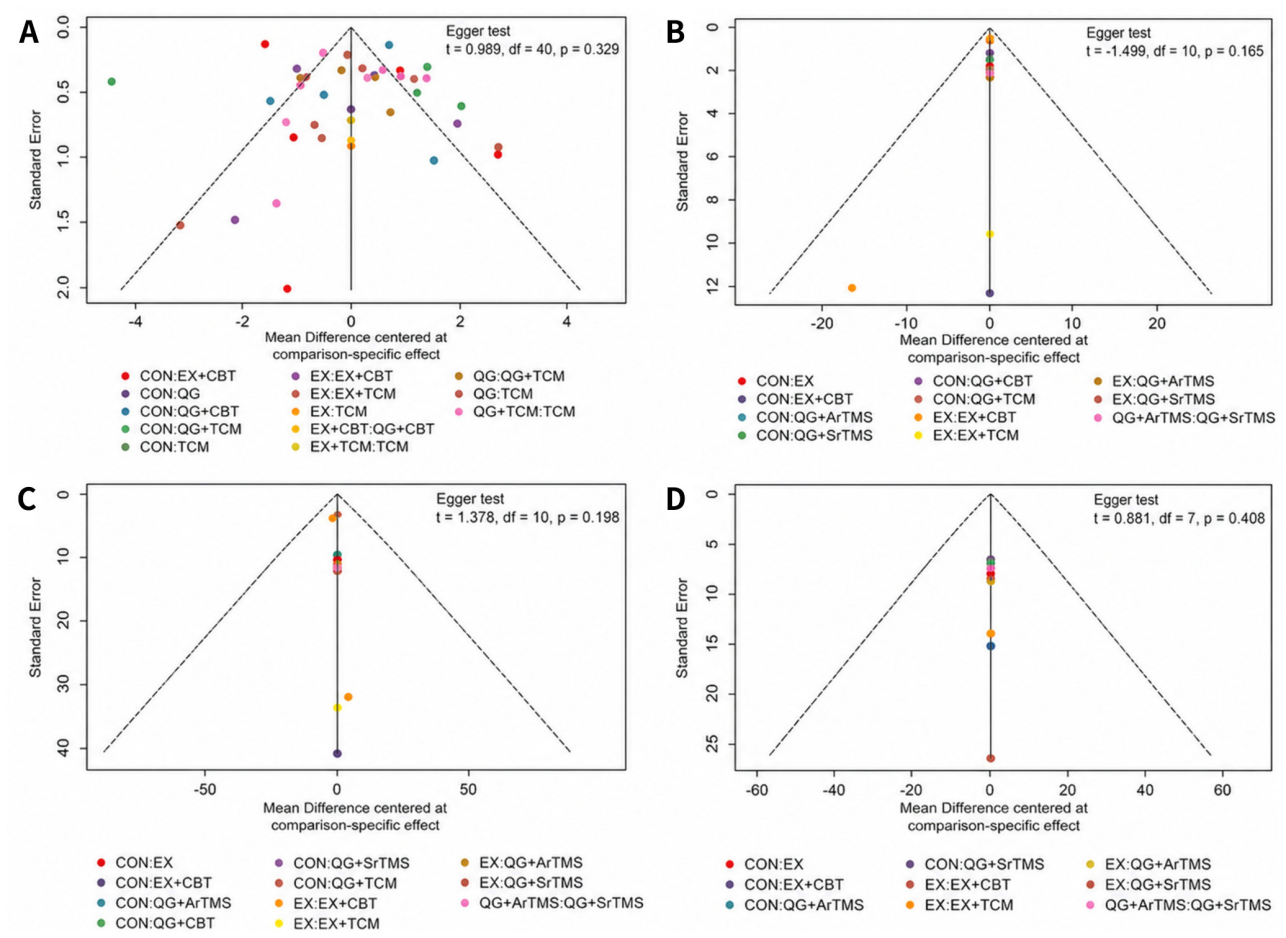
Comparison-adjusted funnel plots for the four sleep outcomes: **(A)** PSQI, **(B)** SOL, **(C)** TST, and **(D)** WASO.

Comparison-adjusted funnel plots combined with Egger's test were used to assess publication bias. The funnel plots for PSQI, TST, SOL, and WASO showed no obvious asymmetry, providing no clear evidence of publication bias. Egger's test also showed no statistically significant results for any outcome (all *p* > 0.05; Supplementary Table 8), which was consistent with the visual inspection of the funnel plots (Figure 6). Individual comparison-adjusted funnel plots are provided in Supplementary Figures 34–37.

#### 3.3.7 Certainty of evidence

According to the CINeMA assessment results (Supplementary Table 9), the overall certainty of evidence was limited. Most comparisons were rated as low or very low confidence, with only a small number of comparisons reaching moderate confidence. The main reasons for downgrading included within-study bias, imprecision, heterogeneity, and incoherence. Therefore, the comparative effects and ranking results should be interpreted cautiously, especially for comparisons supported by sparse direct evidence or showing inconsistency.

## 4 Discussion

This network meta-analysis included 31 RCTs involving 2,457 patients with insomnia or clinically relevant insomnia symptoms and systematically compared the effects of five categories of exercise-based combination interventions against conventional control and single-component interventions. Overall, exercise-based combination interventions showed greater improvements in sleep quality than conventional control and single-component interventions. Among these strategies, EX + CBT ranked highest for the primary outcome, PSQI, and showed certain advantages for some secondary outcomes, representing the most consistently supported pattern under the current, generally limited certainty of evidence.

PSQI is a multidimensional self-rated sleep assessment instrument comprising seven components: subjective sleep quality, sleep latency, sleep duration, habitual sleep efficiency, sleep disturbances, use of sleeping medication, and daytime dysfunction, thereby providing a broad measure of overall sleep quality (27). For PSQI, EX + CBT, QG + CBT, EX + TCM, and QG + TCM significantly reduced PSQI scores compared with CON; among single-component comparators, EX + CBT and EX + TCM were superior to EX, EX + CBT and QG + TCM were superior to TCM, and all four combination strategies were superior to QG. Network meta-regression further indicated that intervention duration significantly moderated PSQI improvement, with longer intervention periods generally associated with greater benefits; accordingly, an adequate intervention duration may be important for achieving more favorable treatment effects. In terms of SUCRA rankings, EX + CBT ranked highest (83.3%), whereas QG + TCM ranked relatively lower (67.0%). Nevertheless, because substantial heterogeneity was observed in the PSQI network and local inconsistency was detected in several comparisons, the SUCRA ranking should be interpreted together with effect estimates, confidence intervals, and evidence certainty, rather than as a stand-alone indicator of superiority. From a mechanistic perspective, EX may influence sleep more directly by increasing physical activity levels and energy expenditure, thereby enhancing sleep propensity and supporting sleep homeostasis (13, 78). By contrast, QG, as a mind–body exercise, may work more through breath regulation and gentle somatic movement, thereby reducing emotional tension, lowering pre-sleep psychophysiological arousal, and promoting mental and physical relaxation (79). Compared with QG, EX may more directly enhance physiological sleep drive, which may partly explain why EX-based combinations appeared to perform better when paired with the same adjunctive intervention. This pattern is consistent with the ranking results reported by Bu et al. (80) in their network meta-analysis and with the findings of Yang et al. (81) in their systematic review and meta-analysis. Therefore, when the adjunctive intervention was the same, EX-based combinations appeared to confer greater benefits than QG-based combinations, suggesting that exercise modality itself may influence intervention effects. However, this interpretation should be considered hypothesis-generating rather than definitive.

Further analysis of the adjunctive intervention component showed that CBT-based combinations ranked higher overall than TCM-based combinations, suggesting that the choice of adjunctive intervention may be an important factor contributing to differences in intervention effects. CBT primarily works by reducing time spent awake in bed, correcting dysfunctional sleep-related beliefs, and decreasing excessive nocturnal worry and sleep-monitoring behaviors (82, 83). Altena et al. (82) reported that excessive time in bed, sleep-related anxiety, and excessive self-monitoring are core psychological drivers of the persistence and recurrence of chronic insomnia. Accordingly, through cognitive restructuring, relaxation training, and sleep hygiene education, CBT may alleviate insomnia symptoms more directly at the cognitive and emotional levels. By contrast, the TCM-based therapies included in the present study mainly consisted of external approaches such as acupuncture, auricular acupressure, massage, and tuina. These interventions may act primarily by modulating autonomic nervous system activity, influencing melatonin secretion and GABAergic neurotransmission, and improving circadian regulation and brain functional activity; however, their direct effects on the core clinical symptoms of insomnia may be comparatively less immediate (84, 85). This interpretation is also supported by previous evidence. In an 8-week randomized clinical trial involving 160 cancer survivors with insomnia, Garland et al. found that CBT was superior to acupuncture in reducing insomnia severity (86). Baglioni et al. (87) further reported in a systematic review and network meta-analysis that CBT showed relatively strong overall performance among available non-pharmacological interventions. Taken together, EX + CBT may offer a relatively stable and promising pattern among exercise-based combination strategies. The EX + CBT vs. CON comparison for PSQI was rated as moderate certainty by CINeMA, lending additional support to EX + CBT as a potentially preferable combination strategy, while recognizing that most other comparisons were supported by low or very low certainty evidence.

SOL is an important indicator of difficulty initiating sleep and, compared with PSQI, more directly reflects the sleep-onset process in patients with insomnia (88). For SOL, EX + CBT, QG + TCM, and QG + CBT all significantly shortened sleep onset latency, with SUCRA values of 95.7%, 72.8%, and 61.3%, respectively. Direct comparisons further showed that EX + CBT was superior to EX alone, suggesting that adding CBT may further enhance the effect of exercise on SOL. This may reflect mechanistic complementarity: CBT may reduce pre-sleep hyperarousal and worry through cognitive restructuring and behavioral regulation, whereas exercise may facilitate sleep initiation by increasing energy expenditure, strengthening sleep homeostasis, and augmenting sleep drive (89, 90). Zhang et al. (91), in a randomized controlled trial in Chinese women with sleep disturbances, reported that 8 weeks of high-intensity circuit training combined with sleep health education improved sleep onset latency, providing additional evidence that exercise combined with a behavioral intervention may improve SOL. Notably, among the QG-based combinations, QG + TCM ranked higher than QG + CBT for SOL, a pattern not fully consistent with the PSQI findings, in which CBT-based combinations generally ranked ahead of TCM-based combinations. Because SOL is more closely linked to the immediate process of sleep initiation, TCM-related therapies such as acupuncture and auricular acupressure may act more directly on sleep onset through modulation of melatonin secretion, facilitation of GABAergic neurotransmission, and improvement of circadian regulation; this may partly explain the relative advantage of QG + TCM for this specific outcome (85, 92). Nevertheless, given the limited direct comparative evidence, this interpretation warrants caution.

WASO refers to the cumulative duration of wakefulness between sleep onset and final awakening and is an important indicator of sleep-maintenance difficulties (88). Compared with PSQI and SOL, WASO focuses more specifically on persistent wakefulness after nocturnal awakenings and may therefore be more susceptible to variation in measurement method, sample size, and study design. In the present NMA, only EX + CBT was superior to conventional control and EX alone for this outcome. Notably, EX + TCM ranked relatively low for both WASO and SOL, suggesting that evidence supporting its effects on sleep-continuity-related parameters remains insufficient. Importantly, the WASO network was supported by only a small number of RCTs, and some intervention nodes were informed by only one direct comparison; therefore, the rankings of interventions such as QG + rTMS and EX + TCM should be considered exploratory and unstable. A systematic review by Lowe et al. (90) similarly suggested that the evidence for exercise-related improvement in WASO is inconsistent. Overall, the findings for WASO should be interpreted cautiously, and the relative advantages of different combination strategies for sleep-maintenance difficulties still require confirmation in adequately powered, rigorously designed RCTs.

Although EX + TCM had the highest SUCRA ranking for TST, this should not be interpreted as evidence of statistically significant or clinically confirmed superiority, because ranking probabilities indicate relative ranking probability rather than treatment effectiveness. No exercise-based combination intervention showed a statistically significant advantage over conventional control or single-component interventions in increasing TST. This finding may be closely related to the measurement characteristics of TST itself and the pathophysiology of insomnia. Patients with insomnia commonly focus on difficulty initiating sleep and difficulty maintaining sleep; accordingly, early treatment benefits are more likely to be reflected in shortened SOL and reduced WASO, whereas TST, as a global measure of total nocturnal sleep duration, may improve more slowly and to a more limited extent. This is particularly relevant in CBT-based combination interventions, in which core techniques such as sleep restriction initially require a deliberate reduction in time in bed to rebuild sleep homeostasis; in the short term, this targeted process may objectively constrain gains in TST (93). Previous studies on exercise, qigong, acupuncture, and CBT have likewise shown that direct improvement in TST is often limited (83, 92, 94, 95). Therefore, TST may not be the most sensitive outcome for capturing the combined benefits of insomnia interventions. The non-significant between-strategy differences observed here should not be interpreted simply as evidence that combination interventions were ineffective; rather, they may indicate that early clinical gains are more likely to accumulate through restoration of sleep continuity and optimization of sleep efficiency. Further large-scale, longer-duration randomized controlled trials are warranted to verify and explore this relationship.

This study has several limitations. First, the methodological quality of the included studies and the confidence in the evidence were limited, as most studies were judged as having some concerns or a high risk of bias, and the CINeMA assessment indicated low or very low confidence in most network estimates. Second, differences in participant characteristics, insomnia diagnostic criteria or assessment tools, intervention formats, exercise prescriptions, and intervention duration may have contributed to clinical and methodological heterogeneity and affected the transitivity assumption. Substantial heterogeneity was observed in the PSQI network, and local inconsistency was detected in some comparisons. In particular, the inclusion of both formally diagnosed insomnia disorder and insomnia symptoms identified using standardized instruments and cut-offs may have increased clinical heterogeneity. Although diagnostic criteria or assessment instruments were explored in network meta-regression, this analysis was exploratory and could not fully eliminate this concern. Third, evidence for several secondary outcomes and intervention nodes was sparse, with limited studies reporting TST, SOL, and WASO and some direct comparisons supported by only one study. Therefore, these effect estimates and SUCRA rankings should be interpreted cautiously and in conjunction with the corresponding confidence intervals and evidence ratings. Fourth, sleep outcomes mainly relied on PSQI and other subjective sleep parameters, whereas objective sleep measurements and long-term follow-up data were not consistently available across the included trials. Future well-designed, adequately powered RCTs with more detailed intervention reporting and comprehensive outcome assessment are needed to further verify the efficacy and applicable populations of exercise-based combination interventions for insomnia disorder.

## 5 Conclusion

Based on the current evidence, exercise-based combination interventions may be beneficial for improving sleep outcomes in adults with insomnia. Among these, EX + CBT emerged as the most consistently supported strategy, ranking highest for PSQI and showing significant effects on SOL and WASO, while no intervention significantly improved TST. However, the findings remain limited by sparse direct evidence and generally low certainty of evidence, and the ranking results should therefore be interpreted as exploratory. Future large-scale randomized controlled trials with standardized protocols, objective sleep assessments, and long-term follow-up are needed to verify these findings.

## Data Availability

The original contributions presented in the study are included in the article/Supplementary material, further inquiries can be directed to the corresponding author.
