# Supplementary material for: Effects of different intervention modalities combined with exercise in patients with insomnia: a systematic review and network meta-analysis
Source: Front Public Health. 2026 Jun 18;14:1873034. doi: 10.3389/fpubh.2026.1873034 (PMC13322952; doi:10.3389/fpubh.2026.1873034)
Supplement: Supplementary file 1 [file Data_Sheet_1.pdf]

1. Supplementary Table

Table S1 PRISMA NMA Checklist of Items to Include When Reporting A Systematic Review Involving a Network Meta-analysis

Section/Topic	Item #	Checklist Item	Reported on Section
**TITLE**			
Title	1	Identify the report as a systematic review incorporating a network meta-analysis (or related form of meta-analysis).	**Title**
**ABSTRACT**			
Structured summary	2	Provide a structured summary including, as applicable: Background: main objectives. Methods: data sources; study eligibility criteria, participants, and interventions; study appraisal; and synthesis methods, such as network meta-analysis. Results: number of studies and participants identified; summary estimates with corresponding confidence intervals; treatment rankings may also be discussed. Discussion/Conclusions: limitations; conclusions and implications of findings. Other: primary source of funding; systematic review registration number with registry name.	**Abstract**
**INTRODUCTION**			
Rationale	3	Describe the rationale for the review in the context of what is already known, including mention of why a network meta-analysis has been conducted.	**Introduction**
Objectives	4	Provide an explicit statement of questions being addressed, with reference to participants, interventions, comparisons, outcomes, and study design (PICOS).	**Abstract; Introduction**
**METHODS**			
Protocol and registration	5	Indicate whether a review protocol exists and if and where it can be accessed; and, if available, provide registration information, including registration number.	**Materials and Methods**
Eligibility criteria	6	Specify study characteristics and report characteristics used as criteria for eligibility, giving rationale. Clearly describe eligible treatments included in the treatment network, and note whether any have been clustered or merged into the same node, with justification.	**Eligibility Criteria**
Information sources	7	Describe all information sources, such as databases with dates of coverage, contact with study authors to identify additional studies, and the date last searched.	**Data Sources and Search Strategy**
Search	8	Present full electronic search strategy for at least one database, including any limits used, such that it could be repeated.	**Data Sources and Search Strategy**
Study selection	9	State the process for selecting studies, including screening, eligibility, included in systematic review, and, if applicable, included in the meta-analysis.	**Study Selection, Data Extraction, and Intervention Coding; Literature search results (Figure 1)**
Data collection process	10	Describe method of data extraction from reports, such as piloted forms, independently and in duplicate, and any processes for obtaining and confirming data from investigators.	**Study Selection, Data Extraction, and Intervention Coding**

Data items	11	List and define all variables for which data were sought, such as PICOS and funding sources, and any assumptions and simplifications made.	**Study Selection, Data Extraction, and Intervention Coding**
**Geometry of the network**	**S1**	Describe methods used to explore the geometry of the treatment network under study and potential biases related to it. This should include how the evidence base has been graphically summarized for presentation, and what characteristics were compiled and used to describe the evidence base to readers.	**Statistical Analysis; Network evidence plots (Figure 2)**
Risk of bias within individual studies	12	Describe methods used for assessing risk of bias of individual studies, including specification of whether this was done at the study or outcome level, and how this information is to be used in any data synthesis.	**Risk of Bias Assessment and Certainty of Evidence Assessment**
Summary measures	13	State the principal summary measures, such as risk ratio or difference in means. Also describe the use of additional summary measures assessed, such as treatment rankings and surface under the cumulative ranking curve (SUCRA) values, as well as modified approaches used to present summary findings from meta-analyses.	**Statistical Analysis; Network meta-analysis results**
Planned methods of analysis	14	Describe the methods of handling data and combining results of studies for each network meta-analysis. This should include handling of multi-arm trials, selection of variance structure, selection of prior distributions in Bayesian analyses, and assessment of model fit.	**Statistical Analysis**
**Assessment of Inconsistency**	**S2**	Describe the statistical methods used to evaluate the agreement of direct and indirect evidence in the treatment network(s) studied. Describe efforts taken to address its presence when found.	**Statistical Analysis; Inconsistency assessment**
Risk of bias across studies	15	Specify any assessment of risk of bias that may affect the cumulative evidence, such as publication bias or selective reporting within studies.	**Statistical Analysis; Publication bias**
Additional analyses	16	Describe methods of additional analyses if done, indicating which were pre-specified. This may include sensitivity or subgroup analyses, meta-regression analyses, alternative formulations of the treatment network, and use of alternative prior distributions for Bayesian analyses, if applicable.	**Network Meta-Regression Analysis**
**RESULTS†**			
Study selection	17	Give numbers of studies screened, assessed for eligibility, and included in the review, with reasons for exclusions at each stage, ideally with a flow diagram.	**Literature search results (Figure 1)**
**Presentation of network structure**	**S3**	Provide a network graph of the included studies to enable visualization of the geometry of the treatment network.	**Network evidence plots (Figure 2)**
**Summary of network geometry**	**S4**	Provide a brief overview of characteristics of the treatment network. This may include commentary on the abundance of trials and randomized patients for the different interventions and pairwise comparisons in the network, gaps of evidence in the treatment network, and potential biases reflected by the network structure.	**Network evidence plots (Figure 2)**
Study characteristics	18	For each study, present characteristics for which data were extracted, such as study size, PICOS, and follow-up period, and provide the citations.	**Characteristics and risk of bias of included studies (Table 1)**
Risk of bias within studies	19	Present data on risk of bias of each study and, if available, any outcome-level assessment.	**Characteristics and risk of bias of included studies**

Results of individual studies	20	For all outcomes considered, present, for each study: 1) simple summary data for each intervention group, and 2) effect estimates and confidence intervals. Modified approaches may be needed to deal with information from larger networks.	**Characteristics and risk of bias of included studies (Table 1); Network meta-analysis results**
Synthesis of results	21	Present results of each meta-analysis done, including confidence intervals. In larger networks, authors may focus on comparisons versus a particular comparator, such as placebo or standard care, with full findings presented in an appendix. League tables and forest plots may be considered to summarize pairwise comparisons. If additional summary measures were explored, such as treatment rankings, these should also be presented.	**Network meta-analysis results (Figure 3) (Figure 4)**
**Exploration for inconsistency**	**S5**	Describe results from investigations of inconsistency. This may include measures of model fit, P values from statistical tests, or summary of inconsistency estimates from different parts of the treatment network.	**Inconsistency assessment**
Risk of bias across studies	22	Present results of any assessment of risk of bias across studies for the evidence base being studied.	**Publication bias; Certainty of evidence (Figure 6)**
Results of additional analyses	23	Give results of additional analyses, if done, such as sensitivity or subgroup analyses, meta-regression analyses, alternative network geometries studied, alternative choice of prior distributions for Bayesian analyses, and so forth.	**Heterogeneity and network meta-regression analysis (Figure 5)**
**DISCUSSION**			
Summary of evidence	24	Summarize the main findings, including the strength of evidence for each main outcome; consider their relevance to key groups, such as healthcare providers, users, and policy-makers.	**Discussion**
Limitations	25	Discuss limitations at study and outcome level, such as risk of bias, and at review level, such as incomplete retrieval of identified research or reporting bias. Comment on the validity of the assumptions, such as transitivity and consistency. Comment on any concerns regarding network geometry, such as avoidance of certain comparisons.	**Discussion**
Conclusions	26	Provide a general interpretation of the results in the context of other evidence, and implications for future research.	**Conclusion**
**FUNDING**			
Funding	27	Describe sources of funding for the systematic review and other support, such as supply of data; role of funders for the systematic review. This should also include information regarding whether funding has been received from manufacturers of treatments in the network and/or whether some of the authors are content experts with professional conflicts of interest that could affect use of treatments in the network.	**Funding**

PICOS = population, intervention, comparators, outcomes, study design.

* Text in italics indicates wording specific to reporting of network meta-analyses that has been added to guidance from the PRISMA statement.

† Authors may wish to plan for use of appendices to present all relevant information in full detail for items in this section.
