# Supplementary material for: Effects of different intervention modalities combined with exercise in patients with insomnia: a systematic review and network meta-analysis
Source: Front Public Health. 2026 Jun 18;14:1873034. doi: 10.3389/fpubh.2026.1873034 (PMC13322952; doi:10.3389/fpubh.2026.1873034)
Supplement: Supplementary file 2 [file Data_Sheet_2.pdf]

Supplementary Materials

Supplementary materials	Page
Supplementary Table 1: Search strategy	2－6
Supplementary Table 2: Included interventions and their definitions	7
Supplementary Table 3: Excluded full-text reports and reasons for exclusion	8–11
Supplementary Table 4: Risk-of-bias assessment for each study	12
Supplementary Table 5: League tables for all outcomes	13－14
Supplementary Table 6: SUCRA rankings for all outcomes	15
Supplementary Table 7: Main results of prespecified univariable network meta-regression	16
Supplementary Table 8: Egger's test results	16
Supplementary Table 9: CINeMA assessment results	17－20
Supplementary Figures 1 –4: Network evidence plots	21－22
Supplementary Figures 5–9: Inconsistency diagnostic plots	23－27
Supplementary Figures 10–13: Network meta-analysis forest plots for PSQI	28－29
Supplementary Figures 14–15: Network meta-analysis forest plots for TST	30
Supplementary Figures 16–17: Network meta-analysis forest plots for SOL	31
Supplementary Figures 18–19: Network meta-analysis forest plots for WASO	32
Supplementary Figures 20–23: SUCRA ranking plots	33－34
Supplementary Figures 24–27: League heatmaps	35－38
Supplementary Figures 28–33: Network meta-regression bubble plots	39－44
Supplementary Figures 34–37: Comparison-adjusted funnel plots	45－48

Supplementary Table 1. Search strategy

Supplementary Table 1.1 Search strategy for PubMed

Step	Search strategy	Results	Date
#1	Sleep Initiation and Maintenance Disorders[MeSH Terms]	21313	2026-03-20
#2	((((((((((((((((((((((((DIMS (Disorders of Initiating[Title/Abstract] OR Maintaining Sleep[Title/Abstract])) OR (Disorders of Initiating[Title/Abstract] OR Maintaining Sleep[Title/Abstract])) OR (Sleeplessness[Title/Abstract])) OR (Insomnia Disorder[Title/Abstract])) OR (Insomnia Disorders[Title/Abstract])) OR (Insomnia[Title/Abstract])) OR (Insomnias[Title/Abstract])) OR (Chronic Insomnia[Title/Abstract])) OR (Insomnia, Chronic[Title/Abstract])) OR (Early Awakening[Title/Abstract])) OR (Awakening, Early[Title/Abstract])) OR (Nonorganic Insomnia[Title/Abstract])) OR (Insomnia, Nonorganic[Title/Abstract])) OR (Primary Insomnia[Title/Abstract])) OR (Insomnia, Primary[Title/Abstract])) OR (Psychophysiological Insomnia[Title/Abstract])) OR (Insomnia, Psychophysiological[Title/Abstract])) OR (Rebound Insomnia[Title/Abstract])) OR(Insomnia, Rebound [Title/Abstract])) OR (Secondary Insomnia[Title/Abstract])) OR (Insomnia, Secondary[Title/Abstract])) OR (Sleep Initiation Dysfunction[Title/Abstract])) OR (Dysfunction, Sleep Initiation[Title/Abstract])) OR (Dysfunctions, Sleep Initiation[Title/Abstract])) OR (Sleep Initiation Dysfunctions[Title/Abstract])) OR (Transient Insomnia[Title/Abstract])) OR (Insomnia, Transient[Title/Abstract])	40067	2026-03-20
#3	Exercise[MeSH Terms]	285872	2026-03-20
#4	(((((((((((((((((((((((Exercises[Title/Abstract]) OR (Exercise, Physical[Title/Abstract])) OR (Exercises, Physical[Title/Abstract])) OR (Physical Exercise[Title/Abstract])) OR (Physical Exercises[Title/Abstract])) OR (Exercise, Aerobic[Title/Abstract])) OR (Aerobic Exercise[Title/Abstract])) OR (Aerobic Exercises[Title/Abstract])) OR (Exercises, Aerobic[Title/Abstract])) OR (Exercise, Isometric[Title/Abstract])) OR (Exercises, Isometric[Title/Abstract])) OR (Isometric Exercises[Title/Abstract])) OR (Isometric Exercise[Title/Abstract])) OR (Acute Exercise[Title/Abstract])) OR (Acute Exercises[Title/Abstract])) OR (Exercise, Acute[Title/Abstract])) OR (Exercises, Acute[Title/Abstract])) OR (Exercise Training[Title/Abstract])) OR (Exercise Trainings[Title/Abstract])) OR (Training, Exercise[Title/Abstract])) OR (Trainings, Exercise[Title/Abstract])) OR (Physical Activity[Title/Abstract])) OR (Activities, Physical[Title/Abstract])) OR (Activity, Physical[Title/Abstract])) OR (Physical Activities[Title/Abstract])	366495	2026-03-20
#5	Physical Education and Training[MeSH Terms]	14629	2026-03-20
#6	((Physical Education, Training[Title/Abstract]) OR (Physical Education[Title/Abstract])) OR (Education, Physical[Title/Abstract])	9048	2026-03-20
#7	(((((((((((randomized controlled trial[Publication Type]) OR (controlled clinical trial[Publication Type])) OR (clinical trials as topic[MeSH Terms])) OR (random allocation[MeSH Terms])) OR (randomized controlled trials as topic[MeSH Terms])) OR (clinical trial[Publication Type])) OR (clinical trial[Text Word])) OR (singl*[Text Word])) OR (doubl*[Text Word])) OR (trebl*[Text Word])) OR (tripl*[Text Word])) OR (randomly[Text Word])) OR (trial[Text Word])	5080151	2026-03-20
#8	#1 OR #2	45233	2026-03-20
#9	#3 OR #4 OR #5 OR #6	519860	2026-03-20
#10	#8 AND #9 AND #7	480	2026-03-20

Supplementary Table 1.2 Search strategy for Web of Science Database

Step	Search strategy	Results	Date
#1	TS=("Sleep Initiation and Maintenance Disorders" OR DIMS OR "Disorders of Initiating and Maintaining Sleep" OR Sleeplessness OR "Insomnia Disorder" OR "Insomnia Disorders" OR Insomnia OR Insomnias OR "Chronic Insomnia" OR "Insomnia, Chronic" OR "Early Awakening" OR "Awakening, Early" OR "Nonorganic Insomnia" OR "Insomnia, Nonorganic" OR "Primary Insomnia" OR "Insomnia, Primary" OR "Psychophysiological Insomnia" OR "Insomnia, Psychophysiological" OR "Rebound Insomnia" OR "Insomnia, Rebound" OR "Secondary Insomnia" OR "Insomnia, Secondary" OR "Sleep Initiation Dysfunction" OR "Dysfunction, Sleep Initiation" OR "Dysfunctions, Sleep Initiation" OR "Sleep Initiation Dysfunctions" OR "Transient Insomnia" OR "Insomnia, Transient")	82292	2026-03-20
#2	TS=("Exercise" OR Exercises OR "Exercise, Physical" OR "Exercises, Physical" OR "Physical Exercise" OR "Physical Exercises" OR "Exercise, Aerobic" OR "Aerobic Exercise" OR "Aerobic Exercises" OR "Exercises, Aerobic" OR "Exercise, Isometric" OR "Exercises, Isometric" OR "Isometric Exercises" OR "Isometric Exercise" OR "Acute Exercise" OR "Acute Exercises" OR "Exercise, Acute" OR "Exercises, Acute" OR "Exercise Training" OR "Exercise Trainings" OR "Training, Exercise" OR "Trainings, Exercise" OR "Physical Activity" OR "Activities, Physical" OR "Activity, Physical" OR "Physical Activities" OR "Physical Education and Training" OR "Physical Education, Training" OR "Physical Education" OR "Education, Physical")	907446	2026-03-20
#3	TS=("randomized controlled trial" OR "controlled clinical trial" OR "clinical trials as topic" OR "random allocation" OR "randomized controlled trials as topic" OR "clinical trial" OR singl* OR doubl* OR trebl* OR tripl* OR randomly OR trial)	8894099	2026-03-20
#4	#1 AND #2 AND #3	1092	2026-03-20

Supplementary Table 1.3 Search strategy for Cochrane Library

Step	Search strategy	Results	Date
#1	Search: ("Exercise" OR "Exercises" OR "Physical exercise" OR "Physical exercises" OR "Exercise, physical" OR "Exercises, physical" OR "Physical activity" OR "Physical activities" OR "Activity, physical" OR "Activities, physical" OR "Aerobic exercise" OR "Aerobic exercises" OR "Exercise, aerobic" OR "Exercises, aerobic" OR "Isometric exercise" OR "Isometric exercises" OR "Exercise, isometric" OR "Exercises, isometric" OR "Acute exercise" OR "Acute exercises" OR "Exercise, acute" OR "Exercises, acute" OR "Exercise training" OR "Exercise trainings" OR "Training, exercise" OR "Trainings, exercise" OR "Resistance exercise" OR "Resistance exercises" OR "Walking" OR "Jogging")	212312	2026-03-20
#2	Search: ("Sleep initiation and maintenance disorders" OR "Sleep initiation and maintenance disorder" OR "Disorders of initiating and maintaining sleep" OR "DIMS" OR "Sleeplessness" OR "Insomnia" OR "Insomnias" OR "Insomnia disorder" OR "Insomnia disorders" OR "Chronic insomnia" OR "Insomnia, chronic" OR "Primary insomnia" OR "Insomnia, primary" OR "Psychophysiological insomnia" OR "Insomnia, psychophysiological" OR "Nonorganic insomnia" OR "Insomnia, nonorganic" OR "Secondary insomnia" OR "Insomnia, secondary" OR "Transient insomnia" OR "Insomnia, transient" OR "Rebound insomnia" OR "Insomnia, rebound" OR "Early awakening" OR "Awakening, early" OR "Sleep initiation dysfunction" OR "Sleep initiation dysfunctions" OR "Dysfunction, sleep initiation" OR "Dysfunctions, sleep initiation")	18720	2026-03-20
#3	Search: ("Randomized controlled trial" OR "Randomized controlled trials" OR "Randomized trial" OR "Randomized trials" OR "Controlled clinical trial" OR "Controlled clinical trials" OR "Clinical trial" OR "Clinical trials" OR "RCT" OR "RCTs" OR "Random allocation" OR "Randomly" OR "Trial" OR "Trials" OR "Placebo")	2357957	2026-03-20
#4	Search: (#1) AND (#2) AND (#3)	1539	2026-03-20

Supplementary Table 1.4 Search strategy for Embase

Step	Search strategy	Results	Date
#1	Search: (DIMS OR 'disorders of initiating and maintaining sleep' OR sleeplessness OR 'insomnia disorder' OR 'insomnia disorders' OR insomnia OR insomnias OR 'chronic insomnia' OR 'insomnia, chronic' OR 'early awakening' OR 'awakening, early' OR 'nonorganic insomnia' OR 'insomnia, nonorganic' OR 'primary insomnia' OR 'insomnia, primary' OR 'psychophysiological insomnia' OR 'insomnia, psychophysiological' OR 'rebound insomnia' OR 'insomnia, rebound' OR 'secondary insomnia' OR 'insomnia, secondary' OR 'sleep initiation dysfunction' OR 'dysfunction, sleep initiation' OR 'dysfunctions, sleep initiation' OR 'sleep initiation dysfunctions' OR 'transient insomnia' OR 'insomnia, transient')	68089	2026-03-20
#2	Search: (exercise OR exercises OR 'exercise, physical' OR 'exercises, physical' OR 'physical exercise' OR 'physical exercises' OR 'exercise, aerobic' OR 'aerobic exercise' OR 'aerobic exercises' OR 'exercises, aerobic' OR 'exercise, isometric' OR 'exercises, isometric' OR 'isometric exercises' OR 'isometric exercise' OR 'acute exercise' OR 'acute exercises' OR 'exercise, acute' OR 'exercises, acute' OR 'exercise training' OR 'exercise trainings' OR 'training, exercise' OR 'trainings, exercise' OR 'physical activity' OR 'activities, physical' OR 'activity, physical' OR 'physical activities' OR 'physical education and training' OR 'physical education, training' OR 'physical education' OR 'education, physical' OR walking OR jogging OR 'resistance exercise' OR 'resistance exercises')	303986	2026-03-20
#3	Search: ('randomized controlled trial' OR 'controlled clinical trial' OR 'clinical trial' OR 'randomized trial' OR 'controlled trial' OR RCT OR random* OR placebo* OR trial OR randomly)	344861	2026-03-20
#4	Search: (#1) AND (#2) AND (#3)	359	2026-03-20

Supplementary Table 1.5 Search strategy for China National Knowledge Infrastructure (CNKI)

Step	Search strategy	Results	Date
#1	主题: "运动+体育锻炼+身体活动+有氧运动+抗阻运动+运动训练"	3563954	2026-03-20
#2	主题: "联合治疗+联合干预+联合应用+辅助治疗+附加治疗+综合治疗+联合疗法"	906366	2026-03-20
#3	主题: "失眠+不寐+失眠障碍"	83215	2026-03-20
#4	主题: (#1) AND 主题: (#2) AND 主题: (#3)	76	2026-03-20

Supplementary Table 1.6 Search strategy for VIP Chinese Journal Database (CQVIP)

Step	Search strategy	Results	Date
#1	题名或关键词 = （联合治疗 OR 联合干预 OR 联合应用 OR 辅助治疗 OR 附加治疗 OR 综合治疗 OR 联合疗法）	764855	2026-03-20
#2	题名或关键词 = （失眠 OR 不寐 OR 失眠障碍）	36039	2026-03-20
#3	题名或关键词 =（运动 OR 体育锻炼 OR 身体活动 OR 有氧运动 OR 抗阻运动 OR 运动训练）	761119	2026-03-20
#4	题名或关键词 =（#1 AND #2 AND #3）	27	2026-03-20

Supplementary Table 2. Included interventions and their definitions

Component	Full name	Operational definition	Examples in this review
EX	Exercise therapy	Planned, structured, and repeated physical activity or exercise training aimed at improving or maintaining physical function (1).	Aerobic exercise, walking, exercise training, circuit training
QG	Traditional Chinese mind–body exercise	Traditional Chinese mind–body exercise involving coordinated body posture or movement, breathing regulation, and attentional focus (2, 3).	Baduanjin, Tai Chi, Wuqinxi, sitting Baduanjin
TCM	Traditional Chinese medicine non-pharmacological therapy	Non-exercise, non-pharmacological interventions rooted in traditional Chinese medicine theory, mainly involving acupoint stimulation, meridian-based regulation, or external therapeutic approaches (4, 5).	Acupuncture, auricular acupressure, massage, acupoint application, moxibustion, herbal foot bath
CBT	Cognitive behavioral therapy-based intervention	Structured psychological or behavioral interventions for insomnia or sleep improvement based on cognitive and behavioral principles (6).	CBT, CBT-I, sleep health intervention

1. Caspersen CJ, Powell KE, Christenson GM. Physical activity, exercise, and physical fitness: Definitions and distinctions for health-related research. *Public Health Rep*. (1985) 100:126–31.

2. Jahnke R, Larkey L, Rogers C, Etnier J, Lin F. A comprehensive review of health benefits of qigong and tai chi. *Am J Health Promot*. (2010) 24:e1–e25. doi: 10.4278/ajhp.081013-LIT-248

3. Yu Y, Wu T, Wu M, Liu S, Chen X, Wu J, et al. Evidence map of traditional chinese exercises. *Front Public Health*. (2024) 12:1347201. doi: 10.3389/fpubh.2024.1347201

4. World Health Organization. Who international standard terminologies on traditional chinese medicine. Geneva, editor. World Health Organization; 2022. [3 March 2022]. Available from:https://www.who.int/publications/i/item/9789240042322 . 453.

5. Matos LC, Machado JP, Monteiro FJ, Greten HJ. Understanding traditional Chinese medicine therapeutics: An overview of the basics and clinical applications. *Healthcare*. (2021) 9:257. doi: 10.3390/healthcare9030257

6. Edinger JD, Arnedt JT, Bertisch SM, Carney CE, Harrington JJ, Lichstein KL, et al. Behavioral and psychological treatments for chronic insomnia disorder in adults: An american academy of sleep medicine systematic review, meta-analysis, and grade assessment. *J Clin Sleep Med*. (2021) 17:263–298. doi: 10.5664/jcsm.8988

Supplementary Table 3: Excluded full-text reports and reasons for exclusion

Study	Reason for exclusion
Jansson-Fröjmark et al. (2025)(1)	Non-exercise intervention
Baron et al. (2023)(2)	Non-insomnia population
Hartescu et al. (2020)(3)	Irrelevant outcomes
Gulia et al. (2023)(4)	Type of study did not match
Baron et al. (2013)(5)	Conference abstract
Michelson et al. (2014)(6)	Non-exercise intervention
Noyan et al. (2024)(7)	Non-insomnia population
Jamshidi et al. (2019)(8)	Irrelevant outcomes
Montgomery et al. (2002)(9)	Type of study did not match
Lv et al. (2011)(10)	Conference abstract
Horsch et al. (2017)(11)	Non-exercise intervention
Teruel-Hernandez et al. (2023)(12)	Non-insomnia population
Li et al. (2024)(13)	Irrelevant outcomes
McGranahan et al. (2024)(14)	Type of study did not match
D'Aurea et al. (2018)(15)	Conference abstract
Kaku et al. (2012)(16)	Non-exercise intervention
Buchanan et al. (2017)(17)	Non-insomnia population
Chin et al. (2022)(18)	Irrelevant outcomes
Lo et al. (2014)(19)	Type of study did not match
Ferreira et al. (2019)(20)	Conference abstract
Reynolds et al. (2010)(21)	Non-exercise intervention
Mendham et al. (2021)(22)	Non-insomnia population
Zhang et al. (2020)(23)	Irrelevant outcomes
Youngstedt (2005)(24)	Type of study did not match
Ji et al. (2019)(25)	Conference abstract
Abbasi et al. (2012)(26)	Non-exercise intervention
Niu et al. (2021)(27)	Non-insomnia population
Irwin et al. (2014)(28)	Irrelevant outcomes
Markwald et al. (2018)(29)	Type of study did not match
Akyar et al. (2013)(30)	Non-exercise intervention
Lunasin et al. (2025)(31)	Non-insomnia population
Irwin et al. (2015)(32)	Irrelevant outcomes
Sherrill et al. (1998)(33)	Type of study did not match
Wong et al. (2022)(34)	Non-exercise intervention
Lindegård et al. (2022)(35)	Non-insomnia population
Malfliet et al. (2019)(36)	Type of study did not match
Kakuei et al. (2025)(37)	Non-exercise intervention
Ezpeleta et al. (2023)(38)	Non-insomnia population
Hachul et al. (2014)(39)	Type of study did not match
Pickett et al. (2024)(40)	Non-exercise intervention
Turmel et al. (2022)(41)	Type of study did not match
Sunnhed et al. (2020)(42)	Non-exercise intervention
Erlacher et al. (2015)(43)	Type of study did not match

Jha et al. (2023)(44)	Non-exercise intervention
Barrett et al. (2020)(45)	Type of study did not match
Manber et al. (2019)(46)	Non-exercise intervention
Hürlimann et al. (2023)(47)	Non-exercise intervention
Freedman et al. (1976)(48)	Non-exercise intervention
Zakiei et al. (2021)(49)	Non-exercise intervention
Ji et al. (2017)(50)	Non-exercise intervention

1. Jansson-Fröjmark M, Sunnhed R. Smartphone application-delivered cognitive behavioural therapy for insomnia with telephone support for insomnia disorder compared to a waitlist control: A randomised clinical trial. *Journal of Sleep Research*. (2025) 34: doi: 10.1111/jsr.14363

2. Baron K, Duffecy J, Simonsen S, Bress A, Conroy M, Greene T, et al. Sleep technology intervention to target cardiometabolic health (stitch): A randomized controlled study of a behavioral sleep extension intervention compared to an education control to improve sleep duration, blood pressure, and cardiometabolic health among adults with elevated blood pressure/hypertension. *Trials*. (2023) 24: doi: 10.1186/s13063-023-07658-6

3. Hartescu I, Morgan K, Stevinson CD. Psychomotor performance decrements following a successful physical activity intervention for insomnia. *Behavioral sleep medicine*. (2020) 18:298–308. doi: 10.1080/15402002.2019.1578774

4. Gulia K, Sreedharan S. Yoga nidra, a nonpharmacological technique in management of insomnia and overall health in postmenopausal women. *Sleep Medicine Clinics*. (2023) 18:463–471. doi: 10.1016/j.jsmc.2023.06.007

5. Baron KG, Reid KJ, Wolfe LF, Naylor E, Zee P. Structured activity and aerobic exercise improve actigraphically estimated sleep among older adults with insomnia. *Sleep*. (2013) 36:A198–A199.

6. Michelson D, Snyder E, Paradis E, Chengan-Liu M, Snavely DB, Hutzelmann J, et al. Safety and efficacy of suvorexant during 1-year treatment of insomnia with subsequent abrupt treatment discontinuation: A phase 3 randomised, double-blind, placebo-controlled trial. *The lancet Neurology*. (2014) 13:461–471. doi: 10.1016/S1474-4422(14)70053-5

7. Noyan G, Direk G, Örengul A. A randomized controlled trial of effects of sleep hygiene training and progressive muscle relaxation training in children with adhd. *Sleep Medicine*. (2024) 117:169–176. doi: 10.1016/j.sleep.2024.03.001

8. Jamshidi O, Seghatoleslami A, Salmanpour M. Low intensity aerobic exercise improves the motor perceptual functions in aged males with insomnia. *Sleep and hypnosis*. (2019) 21:112–116. doi: 10.5350/Sleep.Hypn.2019.21.0179

9. Montgomery P, Dennis J. Physical exercise for sleep problems in adults aged 60+. *Cochrane Database Syst Rev*. (2002) 2002:Cd003404. doi: 10.1002/14651858.Cd003404

10. Lv X, Sun S, Wang W. The sleep-improving efficacy of mind-tranquilization caring exercise on female patients with insomnia. *Sleep medicine*. (2011) 12:S101.

11. Horsch C, Spruit S, Lancee J, van Eijk R, Beun R, Neerincx M, et al. Reminders make people adhere better to a self-help sleep intervention. *Health and Technology*. (2017) 7:173–188. doi: 10.1007/s12553-016-0167-x

12. Teruel-Hernandez E, Lopez-Pina JA, Souto-Camba S, Baez-Suarez A, Medina-Ramirez R, Gomez-Conesa A. Improving sleep quality, daytime sleepiness, and cognitive function in patients with dementia by therapeutic exercise and nesa neuromodulation: A multicenter clinical trial. *International journal of environmental research and public health*. (2023) 20: doi: 10.3390/ijerph20217027

13. Li L, Xie X, Jiang H, Yu J. Improving memory through better sleep in community-dwelling older adults: A tai chi intervention study. *Journals of Gerontology Series B-Psychological Sciences and Social Sciences*. (2024) 79: doi: 10.1093/geronb/gbae156

14. McGranahan MJ, O'Connor PJ. Influence of regular physical activity on sleep. *Curr Top Behav Neurosci*. (2024) 67:309–328. doi: 10.1007/7854_2024_503

15. D'Aurea CV, Santana MG, Passos GS, Youngstedt SD, Poyares D, De Souza AA, et al. Mood and quality of life of patients with chronic insomnia after resistance exercise and stretching. *Sleep*. (2018) 41:A145.

16. Kaku A, Nishinoue N, Takano T, Eto R, Kato N, Ono Y, et al. Randomized controlled trial on the effects of a combined sleep hygiene education and behavioral approach program on sleep quality in workers with insomnia. *Industrial Health*. (2012) 50:52–59. doi: 10.2486/indhealth.MS1318

17. Buchanan D, Landis C, Hohensee C, Guthrie K, Otte J, Paudel M, et al. Effects of yoga and aerobic exercise on actigraphic sleep parameters in menopausal women with hot flashes. *Journal of Clinical Sleep Medicine*. (2017) 13:11–18. doi: 10.5664/jcsm.6376

18. Chin E, Yu A, Leung C, Bernal J, Au W, Fong D, et al. Effects of exercise frequency and intensity on reducing depressive symptoms in older adults with insomnia: A pilot randomized controlled trial. *Frontiers in Physiology*. (2022) 13: doi: 10.3389/fphys.2022.863457

19. Lo C, Lee P. Feasibility and effects of tai chi for the promotion of sleep quality and quality of life a single-group study in a sample of older chinese individuals in hong kong. *Journal of Gerontological Nursing*. (2014) 40:46–52. doi: 10.3928/00989134-20131028-08

20. Ferreira WS, Santana MG, Assis DE, Assis BP, De Cerqueira DP, Oliveira DM, et al. The effects of exercise training versus exercise plus acupuncture on chronic insomnia: Preliminary results. *Sleep Science*. (2019) 12:45.

21. Reynolds C, Serody L, Okun M, Hall M, Houck P, Patrick S, et al. Protecting sleep, promoting health in later life: A randomized clinical trial. *Psychosomatic Medicine*. (2010) 72:178–186. doi: 10.1097/PSY.0b013e3181c870a5

22. Mendham A, Goedecke J, Fortuin-de Smidt M, Phiri L, Clamp L, Swart J, et al. Improved sleep quality and depressive symptoms with exercise training in obese women from a low socioeconomic community: A randomized controlled trial. *Journal of Physical Activity & Health*. (2021) 18:440–449. doi: 10.1123/jpah.2020-0648

23. Zhang X, Wang X, Le S, Ojanen X, Tan X, Wiklund P, et al. Effects of exercise and dietary interventions on serum metabolites in men with insomnia symptoms: A 6-month randomized controlled trial. *Sports Medicine and Health Science*. (2020) 2:95–101. doi: 10.1016/j.smhs.2020.05.002

24. Youngstedt S. Effects of exercise on sleep. *Clinics in Sports Medicine*. (2005) 24:355–+. doi: 10.1016/j.csm.2004.12.003

25. Ji Y, Ma S. The effect of taichi for college students on sleep disorders: A randomized control. *Basic & clinical pharmacology & toxicology*. (2019) 125:81–82. doi: 10.1111/bcpt.13226

26. Abbasi B, Kimiagar M, Sadeghniiat K, Shirazi MM, Hedayati M, Rashidkhani B. The effect of magnesium supplementation on primary insomnia in elderly: A double-blind placebo-controlled clinical trial. *Journal of research in medical sciences*. (2012) 17:1161–1169.

27. Niu S, Lin C, Chen P, Fan Y, Huang H, Chiu H. Immediate and lasting effects of aerobic exercise on the actigraphic sleep parameters of female nurses: A randomized controlled trial. *Research in Nursing & Health*. (2021) 44:449–457. doi: 10.1002/nur.22126

28. Irwin M, Olmstead R, Carrillo C, Sadeghi N, Breen E, Witarama T, et al. Cognitive behavioral therapy vs. Tai chi for late life insomnia and inflammatory risk: A randomized controlled comparative efficacy trial. *Sleep*. (2014) 37:1543–U361. doi: 10.5665/sleep.4008

29. Markwald R, Iftikhar I, Youngstedt S. Behavioral strategies, including exercise, for addressing insomnia. *Acsms Health & Fitness Journal*. (2018) 22:23–29. doi: 10.1249/fit.0000000000000375

30. Akyar I, Akdemir N. The effect of light therapy on the sleep quality of the elderly: An intervention study. *Australian Journal of Advanced Nursing*. (2013) 31:31–38.

31. Lunasin R, Cleland J, Brizzolara K, Weber M, Wang-Price S. The effects of dry needling and therapeutic exercise on sleep in individuals with chronic neck pain and sleep disturbance: A feasibility randomized clinical trial. *Journal of Manual & Manipulative Therapy*. (2025) doi: 10.1080/10669817.2025.2581047

32. Irwin M, Olmstead R, Breen E, Witarama T, Carrillo C, Sadeghi N, et al. Cognitive behavioral therapy and tai chi reverse cellular and genomic markers of inflammation in late-life insomnia: A randomized controlled trial. *Biological Psychiatry*. (2015) 78:721–729. doi: 10.1016/j.biopsych.2015.01.010

33. Sherrill D, Kotchou K, Quan S. Association of physical activity and human sleep disorders. *Archives of Internal Medicine*. (1998) 158:1894–1898. doi: 10.1001/archinte.158.17.1894

34. Wong KF, Perini F, Lin J, Goldstein M, Ong JL, Lo J, et al. Dissociable changes in sleep architecture with mindfulness and sleep hygiene intervention in older adults: Secondary and exploratory analysis of polysomnography data from the mindfulness sleep therapy (mist) trial. *Sleep health*. (2022) 8:364–372. doi: 10.1016/j.sleh.2022.02.003

35. Lindegård A, Glise K, Wiegner L, Reinhardt P, Ellbin S, Pettersson S, et al. Effects of additional individually tailored interventions on sick-leave and symptoms in patients with exhaustion disorder: A randomized controlled trial. *Journal of rehabilitation medicine*. (2022) 54:jrm00321. doi: 10.2340/jrm.v54.2941

36. Malfliet A, Bilterys T, Van Looveren E, Meeus M, Danneels L, Ickmans K, et al. The added value of cognitive behavioral therapy for insomnia to current best evidence physical therapy for chronic spinal pain: Protocol of a randomized controlled clinical trial. *Brazilian journal of physical therapy*. (2019) 23:62–70. doi: 10.1016/j.bjpt.2018.10.007

37. Kakuei A, Ravari A, Mirzaei T, Kamiab Z, Bahrami R. Comparison of diaphragmatic breathing relaxation training and cognitive-behavioral therapy on sleep quality in the elderly: A randomized clinical trial. *Schlaf & Atmung [Sleep & breathing]*. (2025) 29:131. doi: 10.1007/s11325-025-03296-1

38. Ezpeleta M, Gabel K, Cienfuegos S, Kalam F, Lin S, Pavlou V, et al. Alternate-day fasting combined with exercise: Effect on sleep in adults with obesity and nafld. *Nutrients*. (2023) 15: doi: 10.3390/nu15061398

39. Hachul H, Monson C, Kozasa E, Oliveira D, Goto V, Afonso R, et al. Complementary and alternative therapies for treatment of insomnia in women in postmenopause. *Climacteric*. (2014) 17:645–653. doi: 10.3109/13697137.2014.926321

40. Pickett S, Kozak A, Lanni D, Warnke A, Gaillard P, Jarrett N. The comparison of brief, online mindfulness and relaxation interventions to reduce stress and improve sleep-related outcomes in college students. *Journal of American College Health*. (2024) 72:1085–1093. doi: 10.1080/07448481.2022.2066979

41. Turmel D, Carlier S, Bruyneel A, Bruyneel M. Tailored individual yoga practice improves sleep quality, fatigue, anxiety, and depression in chronic insomnia disorder. *Bmc Psychiatry*. (2022) 22: doi: 10.1186/s12888-022-03936-w

42. Sunnhed R, Hesser H, Andersson G, Carlbring P, Morin C, Harvey A, et al. Comparing internet-delivered cognitive therapy and behavior therapy with telephone support for insomnia disorder: A randomized controlled trial. *Sleep*. (2020) 43: doi: 10.1093/sleep/zsz245

43. Erlacher C, Erlacher D, Schredl M. The effects of exercise on self-rated sleep among adults with chronic sleep complaints. *Journal of Sport and Health Science*. (2015) 4:289–298. doi: 10.1016/j.jshs.2014.01.001

44. Jha P, Kumar D. Comparative study of efficacy and safety of ramelteon vs zolpidem in patients of insomnia in tertiary care centre of east india. *International journal of pharmaceutical sciences review and research*. (2023) 78:149–155. doi: 10.47583/ijpsrr.2023.v78i02.023

45. Barrett B, Harden C, Brown R, Coe C, Irwin M. Mindfulness meditation and exercise both improve sleep quality: Secondary analysis of a randomized controlled trial of community dwelling adults. *Sleep Health*. (2020) 6:804–813. doi: 10.1016/j.sleh.2020.04.003

46. Manber R, Bei B, Simpson N, Asarnow L, Rangel E, Sit A, et al. Cognitive behavioral therapy for prenatal insomnia a randomized controlled trial. *Obstetrics and Gynecology*. (2019) 133:911–919. doi: 10.1097/aog.0000000000003216

47. Hürlimann P, Bodenmann G, Riemann D, Weitkamp K. Cognitive behavioural therapy to treat stress and insomnia: A randomized wait list-controlled trial of two online courses. *Journal of Sleep Research*. (2023) 32: doi: 10.1111/jsr.13874

48. Freedman R, Papsdorf JD. Biofeedback and progressive relaxation treatment of sleep-onset insomnia: A controlled, all-night investigation. *Biofeedback and self-regulation*. (1976) 1:253–271. doi: 10.1007/BF01001167

49. Zakiei A, Khazaie H, Rostampour M, Lemola S, Esmaeili M, Dursteler K, et al. Acceptance and commitment therapy (act) improves sleep quality, experiential avoidance, and emotion regulation in individuals with insomnia-results from a randomized interventional study. *Life-Basel*. (2021) 11: doi: 10.3390/life11020133

50. Ji XW, Chan CH, Lau BH, Chan JS, Chan CL, Chung KF. The interrelationship between sleep and depression: A secondary analysis of a randomized controlled trial on mind-body-spirit intervention. *Sleep medicine*. (2017) 29:41–46. doi: 10.1016/j.sleep.2016.08.025

Supplementary Table 4. Risk of bias of each study

Study	Randomization process	Deviations from intended interventions	Missing outcome data	Measurement of the outcome	Selection of the reported result	Overall bias
Li 2026	S	S	S	S	S	S
Høeg 2025	S	S	S	H	S	H
Li 2025	S	S	S	S	S	S
Qiu 2025	S	S	S	S	S	S
Wu 2025	S	S	S	S	S	S
Xiong 2025a	S	S	S	S	S	S
Xiong 2025b	S	S	S	S	S	S
Han 2024	S	S	S	S	S	S
He 2024a	S	S	S	S	S	S
He 2024b	S	L	L	S	S	S
Jiang 2024	S	L	S	S	S	S
Xiong 2024	S	S	S	S	S	S
Cammalleri 2023	L	S	H	S	S	H
Chen 2023a	S	S	S	S	S	S
Chen 2023b	S	S	S	S	S	S
He 2023	S	S	S	S	S	S
Liu 2023	S	S	S	S	S	S
Min 2023	S	S	S	S	S	S
Ferreira 2022	S	S	H	S	S	H
Wang 2022	S	S	S	S	S	S
Zhai 2021	S	S	S	S	S	S
Gao 2020	S	S	S	S	S	S
Yang 2020	S	S	L	S	S	S
Wei 2019	L	S	S	S	S	S
Zhang 2019	S	S	S	S	S	S
Ren 2018	L	S	L	S	S	S
Zheng 2018	S	S	S	S	S	S
Song 2017	S	S	S	S	S	S
Wang 2017	H	S	S	H	S	H
Zhao 2016	S	S	S	S	S	S
Reid 2010	S	H	H	H	S	H

Note: L, low risk; S, some concerns; H, high risk.

Supplementary Table 5. League tables for all outcomes

Supplementary Table 5.1 League table for PSQI

	CON	EX	EX+CBT	EX+TCM	QG	QG+CBT	QG+TCM	TCM
CON	CON	.	2.96 [1.42; 4.51]	.	1.45 [-1.80; 4.70]	2.00 [0.39; 3.62]	2.62 [1.06; 4.19]	1.98 [-1.34; 5.30]
EX	0.89 [-0.99; 2.76]	EX	2.51 [0.83; 4.19]	1.43 [-0.32; 3.19]	.	.	.	-0.79 [-4.32; 2.74]
EX+CBT	2.80 [1.45; 4.15]	1.92 [0.38; 3.45]	EX+CBT	.	.	3.31 [-0.18; 6.80]	.	.
EX+TCM	2.68 [0.34; 5.02]	1.79 [0.09; 3.50]	-0.12 [-2.29; 2.05]	EX+TCM	.	.	.	-4.93 [-8.25; -1.61]
QG	-0.27 [-2.12; 1.58]	-1.16 [-3.53; 1.22]	-3.07 [-5.24; -0.91]	-2.95 [-5.62; -0.28]	QG	.	2.47 [0.91; 4.03]	1.19 [-0.38; 2.76]
QG+CBT	2.73 [1.24; 4.21]	1.84 [-0.44; 4.12]	-0.07 [-1.91; 1.76]	0.05 [-2.64; 2.74]	3.00 [0.64; 5.35]	QG+CBT	.	.
QG+TCM	2.19 [0.77; 3.60]	1.30 [-0.79; 3.39]	-0.62 [-2.44; 1.21]	-0.49 [-2.93; 1.94]	2.46 [1.02; 3.89]	-0.54 [-2.57; 1.49]	QG+TCM	-1.48 [-2.61; -0.35]
TCM	0.60 [-1.00; 2.20]	-0.29 [-2.37; 1.80]	-2.20 [-4.11; -0.29]	-2.08 [-4.47; 0.30]	0.87 [-0.59; 2.32]	-2.13 [-4.28; 0.02]	-1.59 [-2.67; -0.50]	TCM

Supplementary Table 5.2 League table for TST

	CON	EX	EX+CBT	EX+TCM	QG+ArTMS	QG+CBT	QG+SrTMS	QG+TCM
CON	CON	-2.70 [-78.78; 73.38]	69.30 [-41.05; 179.65]	.	-3.91 [-79.59; 71.77]	-48.00 [-124.99; 28.99]	2.77 [-73.72; 79.26]	-31.23 [-104.10; 41.64]
EX	26.76 [-38.24; 91.76]	EX	-37.09 [-96.01; 21.83]	51.80 [-50.02; 153.62]	-1.21 [-77.71; 75.29]	.	5.47 [-71.83; 82.77]	.
EX+CBT	7.33 [-65.19; 79.86]	-19.42 [-73.36; 34.51]	EX+CBT	.	.	.	.	.
EX+TCM	78.56 [-42.24; 199.36]	51.80 [-50.02; 153.62]	71.22 [-44.00; 186.45]	EX+TCM	.	.	.	.
QG+ArTMS	10.50 [-62.67; 83.66]	-16.26 [-90.05; 57.53]	3.17 [-82.83; 89.17]	-68.06 [-193.80; 57.69]	QG+ArTMS	.	6.68 [-70.23; 83.59]	.
QG+CBT	-48.00 [-124.99; 28.99]	-74.76 [-175.51; 26.00]	-55.33 [-161.10; 50.43]	-126.56 [-269.80; 16.69]	-58.50 [-164.71; 47.71]	QG+CBT	.	.
QG+SrTMS	17.18 [-56.82; 91.18]	-9.58 [-84.20; 65.04]	9.85 [-76.87; 96.56]	-61.38 [-187.61; 64.86]	6.68 [-70.23; 83.59]	65.18 [-41.61; 171.96]	QG+SrTMS	.
QG+TCM	-31.23 [-104.10; 41.64]	-57.99 [-155.64; 39.66]	-38.56 [-141.37; 64.25]	-109.79 [-250.86; 31.29]	-41.73 [-144.99; 61.53]	16.77 [-89.23; 122.77]	-48.41 [-152.27; 55.45]	QG+TCM

Supplementary Table 5.3 League table for SOL

	CON	EX	EX+CBT	EX+TCM	QG+ArTMS	QG+SrTMS	QG+CBT	QG+TCM
CON	CON	0.68 [-3.04; 4.40]	4.30 [-22.05; 30.65]	.	3.85 [-0.38; 8.08]	-0.66 [-3.81; 2.49]	3.00 [0.52; 5.48]	4.03 [2.65; 5.41]
EX	0.63 [-3.06; 4.31]	EX	6.33 [5.26; 7.40]	-5.30 [-25.41; 14.81]	3.17 [-1.69; 8.03]	-1.34 [-5.29; 2.61]	.	.
EX+CBT	6.95 [3.12; 10.79]	6.33 [5.26; 7.40]	EX+CBT	.	.	.	.	.
EX+TCM	-4.67 [-25.11; 15.77]	-5.30 [-25.41; 14.81]	-11.63 [-31.76; 8.51]	EX+TCM	.	.	.	.
QG+ArTMS	3.83 [-0.40; 8.07]	3.21 [-1.64; 8.06]	-3.12 [-8.08; 1.84]	8.51 [-12.17; 29.19]	QG+ArTMS	-4.51 [-8.95; -0.07]	.	.
QG+SrTMS	-0.68 [-3.82; 2.47]	-1.30 [-5.24; 2.63]	-7.63 [-11.70; -3.55]	4.00 [-16.49; 24.48]	-4.51 [-8.95; -0.07]	QG+SrTMS	.	.
QG+CBT	3.00 [0.52; 5.48]	2.37 [-2.07; 6.82]	-3.95 [-8.52; 0.61]	7.67 [-12.92; 28.26]	-0.83 [-5.74; 4.07]	3.68 [-0.33; 7.68]	QG+CBT	.
QG+TCM	4.03 [2.65; 5.41]	3.40 [-0.53; 7.34]	-2.92 [-7.00; 1.15]	8.70 [-11.78; 29.19]	0.20 [-4.26; 4.65]	4.71 [1.27; 8.14]	1.03 [-1.81; 3.87]	QG+TCM

Supplementary Table 5.4 League table for WASO

	**CON**	**EX**	**EX+CBT**	**EX+TCM**	**QG+ArTMS**	**QG+SrTMS**
**CON**	CON	-0.84 [-19.05; 17.37]	34.60 [0.63; 68.57]	.	5.43 [-10.62; 21.48]	3.87 [-11.41; 19.15]
**EX**	-0.27 [-17.82; 17.28]	EX	27.40 [-29.08; 83.88]	-27.20 [-58.59; 4.19]	6.27 [-13.48; 26.02]	4.71 [-14.42; 23.84]
**EX+CBT**	32.62 [3.13; 62.10]	32.89 [1.05; 64.72]	EX+CBT	.	.	.
**EX+TCM**	-27.47 [-63.43; 8.49]	-27.20 [-58.59; 4.19]	-60.09 [-104.80; -15.38]	EX+TCM	.	.
**QG+ArTMS**	5.60 [-10.38; 21.58]	5.87 [-13.58; 25.32]	-27.01 [-59.81; 5.78]	33.07 [-3.86; 70.00]	QG+ArTMS	-1.56 [-18.64; 15.52]
**QG+SrTMS**	4.04 [-11.17; 19.25]	4.31 [-14.51; 23.13]	-28.57 [-61.00; 3.85]	31.51 [-5.09; 68.11]	-1.56 [-18.64; 15.52]	QG+SrTMS

Supplementary Table 6. SUCRA rankings for all outcomes

Supplementary Table 6.1 SUCRA cumulative probability ranking for PSQI

Treatment	SUCRA(%)	PrBest(%)	MeanRank
EX+CBT	83.3	30	2.2
QG+CBT	80.7	32.5	2.4
EX+TCM	78	31.7	2.5
QG+TCM	67	5.6	3.3
EX	36.9	0.2	5.4
TCM	29.8	0	5.9
CON	13.8	0	7
QG	10.5	0	7.3

Supplementary Table 6.2 SUCRA cumulative probability ranking for TST

Treatment	SUCRA(%)	PrBest(%)	MeanRank
EX+TCM	86.2	67	2
EX	69.7	9.7	3.1
QG+SrTMS	58.7	9	3.9
QG+ArTMS	54.3	7.1	4.2
EX+CBT	51	4.5	4.4
CON	43.5	0.7	5
QG+TCM	21.7	0.9	6.5
QG+CBT	15	1.1	7

Supplementary Table 6.3 SUCRA cumulative probability ranking for SOL

Treatment	SUCRA(%)	PrBest(%)	MeanRank
EX+CBT	95.7	74.5	1.3
QG+TCM	72.8	3.7	2.9
QG+ArTMS	68.9	8.1	3.2
QG+CBT	61.3	2	3.7
EX	33.6	0	5.6
CON	25.2	0	6.2
EX+TCM	23.5	11.7	6.4
QG+SrTMS	19	0	6.7

Supplementary Table 6.4 SUCRA cumulative probability ranking for WASO

Treatment	SUCRA(%)	PrBest(%)	MeanRank
EX+CBT	97.2	92.3	1.1
QG+ArTMS	61.9	4.4	2.9
QG+SrTMS	54.7	2.5	3.3
CON	41.9	0.3	3.9
EX	39.9	0.3	4
EX+TCM	4.4	0.2	5.8

Supplementary Table 7. Main results of prespecified univariable network meta-regression

Moderator	Model term or contrast	Reference/coding/order	β	SE	z	P value	95% CI
Duration of treatment	Slope of Duration weeks	short-term (≤4 weeks) ≥ medium-term (5-8 weeks) ≥ long-term (> 8 weeks)	-1.32	0.38	-3.50	<0.001	-2.07 to -0.58
Sample size	Slope of Sample size	Small sample (n < 60) ≥ Medium sample (60 ≤ n < 100) ≥ Large sample (n ≥ 100)	0.21	0.46	0.46	0.645	-0.69 to 1.12
Comorbidity	Slope of Comorbidity situation	medical comorbidity ≥ neurological comorbidity ≥ No comorbidity	0.48	0.38	1.28	0.201	-0.26 to 1.22
Continent	Slope of Continent	Asia ≥ Europe ≥ North America ≥ South America	-0.08	0.47	-0.17	0.867	-1.01 to 0.85
Diagnostic tool	Slope of Diagnostic tool	CCMD-3 ≥ Chinese guideline ≥ Clinical diagnosis ≥ DSM-5 ≥ ISI ≥ PSQI	0.09	0.20	0.43	0.665	-0.31 to 0.49
Publication year	Slope of Publication year	2015-2019 ≥ 2020-2024 ≥ 2025-2029	-0.09	0.51	-0.17	0.865	-1.08 to 0.91
Duration of treatment	Medium-term (5–8 weeks)	long-term (>8 weeks)	-0.56	1.08	-0.51	0.607	-2.68 to 1.57
Duration of treatment	Short-term (≤4 weeks)	long-term (>8 weeks)	-0.17	1.06	-0.16	0.875	-2.25 to 1.91
Sample size	Medium sample (60 ≤ n < 100)	Large sample (n ≥ 100)	0.27	0.79	0.34	0.732	-1.28 to 1.82
Sample size	Small sample (n < 60)	Large sample (n ≥ 100)	-0.59	1.09	-0.54	0.587	-2.72 to 1.54
Comorbidity	Neurological comorbidity	medical comorbidity	0.76	1.03	0.74	0.462	-1.26 to 2.78
Comorbidity	No comorbidity	medical comorbidity	0.54	0.91	0.59	0.555	-1.25 to 2.32
Continent	Europe	Asia	1.20	2.86	0.42	0.676	-4.42 to 6.81
Continent	North America	Asia	2.60	2.45	1.06	0.289	-2.20 to 7.39
Continent	South America	Asia	-0.14	1.65	-0.09	0.931	-3.37 to 3.09
Diagnostic tool	Chinese guideline	CCMD-3	-1.54	1.32	-1.16	0.245	-4.13 to 1.05
Diagnostic tool	Clinical diagnosis	CCMD-3	-0.96	1.05	-0.91	0.362	-3.01 to 1.10
Diagnostic tool	DSM-5	CCMD-3	-1.23	1.25	-0.99	0.324	-3.67 to 1.21
Diagnostic tool	ISI	CCMD-3	0.41	2.92	0.14	0.889	-5.31 to 6.13
Diagnostic tool	PSQI	CCMD-3	0.35	1.11	0.32	0.752	-1.83 to 2.54
Publication year	2020–2024	2015–2019	0.25	0.86	0.29	0.770	-1.44 to 1.94
Publication year	2025–2029	2015–2019	-0.17	1.09	-0.16	0.873	-2.30 to 1.96

Notes: Estimates were obtained from prespecified univariable network meta-regression models.

β = regression coefficient; SE = standard error; CI = confidence interval. P values are two-sided.

No categorical contrast showed statistically significant moderator effects, whereas the duration-weeks slope in the bubble plot showed a significant negative trend (β = -1.32, P < 0.001).

Supplementary Table 8. Egger's test results

Outcome	k	Intercept	SE	t	df	P value	Interpretation
PSQI	42	0.849	0.858	0.989	40	0.329	No evidence of small-study effects
SOL	12	-0.240	0.160	- 1.499	10	0.165	No evidence of small-study effects
TST	12	0.063	0.046	1.378	10	0.198	No evidence of small-study effects
WASO	9	0.000	0.000	0.881	7	0.408	No evidence of small-study effects

Note: Egger's test was performed using comparison-adjusted funnel plot data. k, number of data points in the comparison-adjusted funnel plot; SE, standard error. P < 0.05 was considered to indicate potential small-study effects. Results for outcomes with fewer than 10 data points should be interpreted cautiously.

Supplementary Table 9. CINeMA assessment results

Supplementary Table 9.1 The overall certainty of evidence assessed using CINeMA: direct evidence

Comparison	Number of studies	Within-study bias	Reporting bias	Indirectness	Imprecision	Heterogeneity	Incoherence	Confidence rating	Reason(s) for downgrading
**PSQI**									
CON : EX+CBT	5	Some concerns	Low risk	No concerns	No concerns	Some concerns	No concerns	Moderate	[Heterogeneity]
CON : QG	1	Some concerns	Low risk	No concerns	Major concerns	No concerns	No concerns	Very low	[Imprecision]
CON : QG+CBT	4	Some concerns	Low risk	No concerns	No concerns	Some concerns	Some concerns	Low	[Heterogeneity]
CON : QG+TCM	4	Some concerns	Low risk	No concerns	No concerns	Major concerns	No concerns	Very low	[Heterogeneity]
CON : TCM	1	Some concerns	Low risk	No concerns	Major concerns	No concerns	No concerns	Very low	[Imprecision]
**TST**									
CON : EX	1	No concerns	Low risk	No concerns	Major concerns	No concerns	No concerns	Low	[Imprecision]
CON : EX+CBT	1	Some concerns	Low risk	No concerns	Major concerns	No concerns	No concerns	Very low	[Within-study bias, Imprecision]
CON : QG+ArTMS	1	No concerns	Low risk	No concerns	Major concerns	No concerns	No concerns	Low	[Imprecision]
CON : QG+SrTMS	1	No concerns	Low risk	No concerns	Major concerns	No concerns	No concerns	Low	[Imprecision]
CON : QG+TCM	1	Some concerns	Low risk	No concerns	Major concerns	No concerns	Major concerns	Very low	[Imprecision, Incoherence]
CON : QG+CBT	1	Some concerns	Low risk	No concerns	Major concerns	No concerns	Major concerns	Very low	[Imprecision, Incoherence]
**SOL**									
CON : EX	1	No concerns	Low risk	No concerns	Major concerns	No concerns	No concerns	Low	[Imprecision]
CON : EX+CBT	1	No concerns	Low risk	No concerns	No concerns	Major concerns	No concerns	Low	[Heterogeneity]
CON : QG+ArTMS	1	No concerns	Low risk	No concerns	Some concerns	Some concerns	No concerns	Moderate	[Heterogeneity]
CON : QG+SrTMS	1	No concerns	Low risk	No concerns	Major concerns	No concerns	No concerns	Low	[Imprecision]

Comparison	Number of studies	Within-study bias	Reporting bias	Indirectness	Imprecision	Heterogeneity	Incoherence	Confidence rating	Reason(s) for downgrading
CON : QG+CBT	1	Some concerns	Low risk	No concerns	No concerns	Major concerns	No concerns	Very low	[Heterogeneity]
CON : QG+TCM	1	Some concerns	Low risk	No concerns	No concerns	No concerns	No concerns	Moderate	[Within-study bias]
**WASO**									
CON : EX	1	No concerns	Low risk	No concerns	Major concerns	No concerns	No concerns	Low	[Imprecision]
CON : EX+CBT	1	Major concerns	Low risk	No concerns	Some concerns	No concerns	No concerns	Very low	[Within-study bias, Imprecision]
CON : QG+ArTMS	1	No concerns	Low risk	No concerns	Major concerns	No concerns	No concerns	Low	[Imprecision]
CON : QG+SrTMS	1	No concerns	Low risk	No concerns	Major concerns	No concerns	No concerns	Low	[Imprecision]

Supplementary Table 9.2 The overall certainty of evidence assessed using CINeMA: indirect evidence

Comparison	Number of studies	Within-study bias	Reporting bias	Indirectness	Imprecision	Heterogeneity	Incoherence	Confidence rating	Reason(s) for downgrading
**PSQI**									
CON : EX	0	Some concerns	Low risk	No concerns	Major concerns	No concerns	Major concerns	Very low	[Imprecision, Incoherence]
CON : EX+TCM	0	Some concerns	Low risk	No concerns	No concerns	Major concerns	Major concerns	Very low	[Heterogeneity, Incoherence]
**TST**									
CON : EX+TCM	0	Some concerns	Low risk	No concerns	Major concerns	No concerns	Major concerns	Very low	[Imprecision, Incoherence]
**SOL**									
CON : EX+TCM	0	Some concerns	Low risk	No concerns	Major concerns	No concerns	No concerns	Low	[Imprecision]
**WASO**									
CON : EX+TCM	0	Some concerns	Low risk	No concerns	Some concerns	Some concerns	No concerns	Low	[Imprecision]

Reason for Downgrading

Based on the recommendations from the CINeMA online documentation (https://cinema.ispm.unibe.ch/), the need for downgrading in each domain was assessed according to the criteria outlined below. The methodology referenced the CINeMA guidance(1).

Within-study bias

Based on the risk-of-bias assessment of the included studies (Supplementary Table 4), each study was judged as "Low risk", "Some concerns", or "High risk" using the revised Cochrane risk-of-bias tool for randomized trials (RoB 2). These judgements were incorporated into the within-study bias domain in CINeMA.

Reporting bias

The search strategy was comprehensive, with no language restrictions applied at the search stage; however, only Chinese and English publications were included at the eligibility stage. Reporting bias was assessed using comparison -adjusted funnel plots presented in Supplementary Figures34–37 and Egger's tests presented in Supplementary Table 8.

Indirectness

Indirectness refers to the relevance of the included studies to the research question. Study populations, interventions, outcomes, and study settings might not be representative of the settings, populations, or outcomes about which reviewers want to make inferences. We addressed this issue by strictly adhering to the inclusion and exclusion criteria (limited to patients with insomnia disorder diagnosed according to recognized standards); therefore, no downgrading was required in this domain.

Imprecision

The outcome measures (PSQI, TST, SOL, WASO) in this network meta-analysis were continuous variables. Given the use of different rating scales or units across studies, mean difference (MD) was selected as the effect measure for continuous variables, calculated based on change scores (final score minus baseline score). Imprecision was judged according to whether the confidence interval crossed the null line and whether it extended beyond the prespecified clinically important effect thresholds within the CINeMA assessment.

Heterogeneity

For heterogeneity assessment, we used the same threshold as the clinically significant threshold described above. Following the automated recommendations of CINeMA, the generation of conclusions was based on the alignment between confidence intervals and prediction intervals. Specifically, CINeMA makes its judgments by evaluating the interval alignment for the following two values: the null effect value (MD=0) and the clinically important effect value in the opposite direction to the point estimate.

Incoherence

In the assessment of incoherence, we employed CINeMA to compare direct and indirect evidence. Global inconsistency was assessed using the design-by-treatment interaction model, and local inconsistency was assessed using the SIDE approach. The specific decision rules were as follows: For estimates informed by both direct and indirect evidence: SIDE test p-value >0.10 → "No concerns"; SIDE test p-value <0.10 → Risk level judged based on the agreement between the 95% confidence intervals of the direct and indirect estimates within the range of clinically important effects. For estimates informed by only direct or only indirect evidence: judgment based on the p-value of the design-by-treatment interaction test. Special case: if no closed evidence loop exists, preventing the tests from being conducted → all comparisons are judged as "Major concerns".

Summarising judgments across the 6 domains

The final output table from CINeMA will present the concern level for each domain. We performed an overall judgment across domains, referencing the four levels of evidence quality (very low, low, moderate, high) from the GRADE framework, applying the following specific rules: (1) High: all domains are "No concerns" → evidence quality remains "high". (2) Moderate: no "Major concerns" and ≤2 domains have "Some concerns" → evidence quality is downgraded to "moderate". (3) Low: no "Major concerns" but ≥3 domains have "Some concerns" → evidence quality is downgraded to "low". (4) Very low: ≥2 domains have "Major concerns", OR 1 domain has "Major concerns" combined with ≥1 domain having "Some concerns" → evidence quality is downgraded to "very low". The 6 CINeMA

domains should therefore be considered jointly rather than in isolation, avoiding downgrading the overall level of confidence more than once for related concerns.

References

1. Nikolakopoulou A, Higgins JPT, Papakonstantinou T, Chaimani A, Del Giovane C, Egger M, et al. Cinema: An approach for assessing confidence in the results of a network meta-analysis. *PLOS Medicine*. (2020) 17:e1003082. doi: 10. 1371/journal.pmed.1003082

Supplementary Figures 1–4. Network evidence plots for PSQI, SOL, TST, and WASO

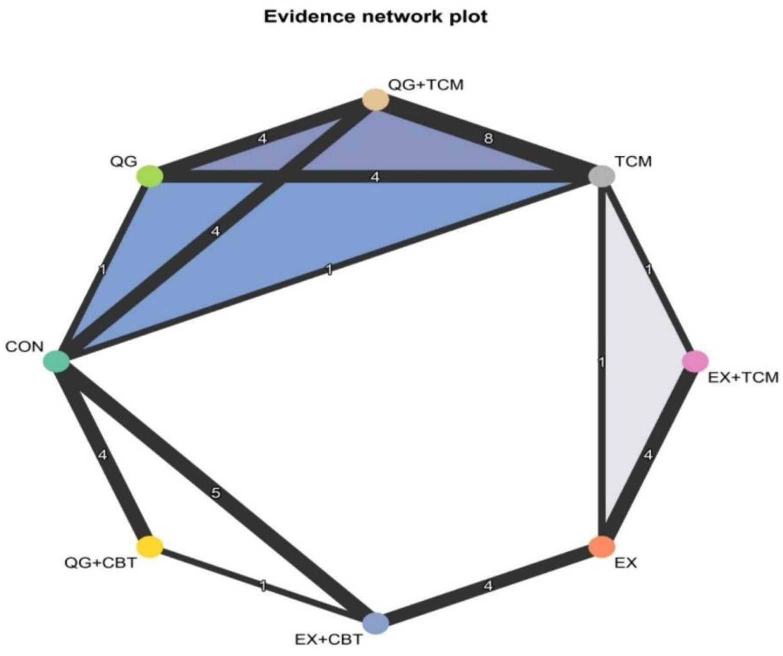

Supplementary Figure 1. Network evidence plot for PSQI

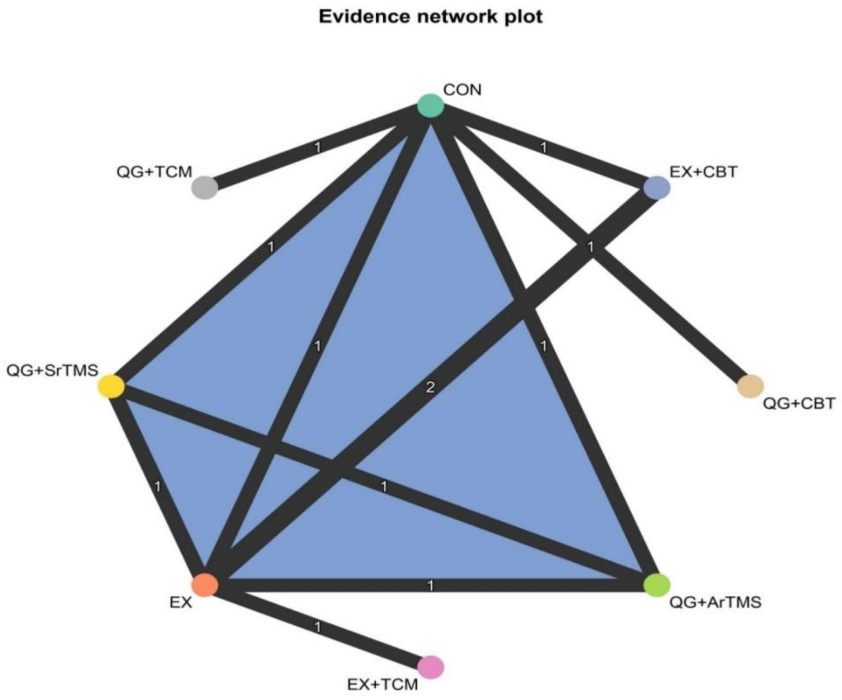

Supplementary Figure 2. Network evidence plot for SOL

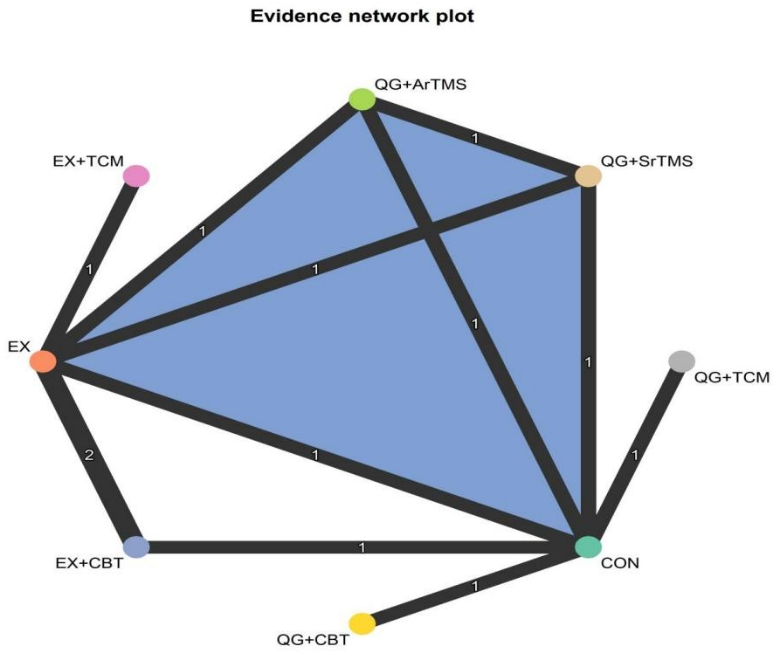

Supplementary Figure 3. Network evidence plot for TST

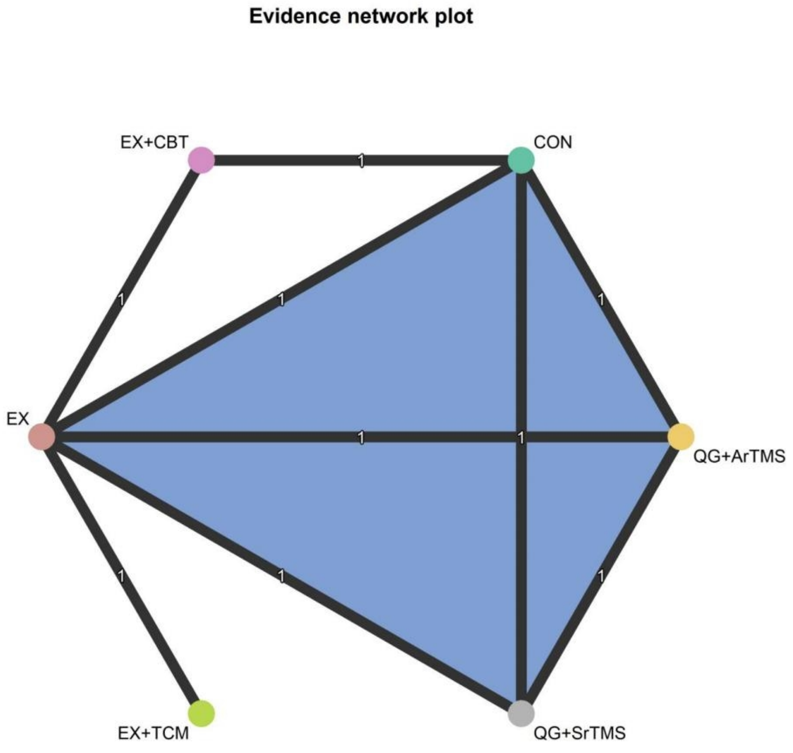

Supplementary Figure 4. Network evidence plot for WASO

Supplementary Figures 5–9. Inconsistency diagnostic plots

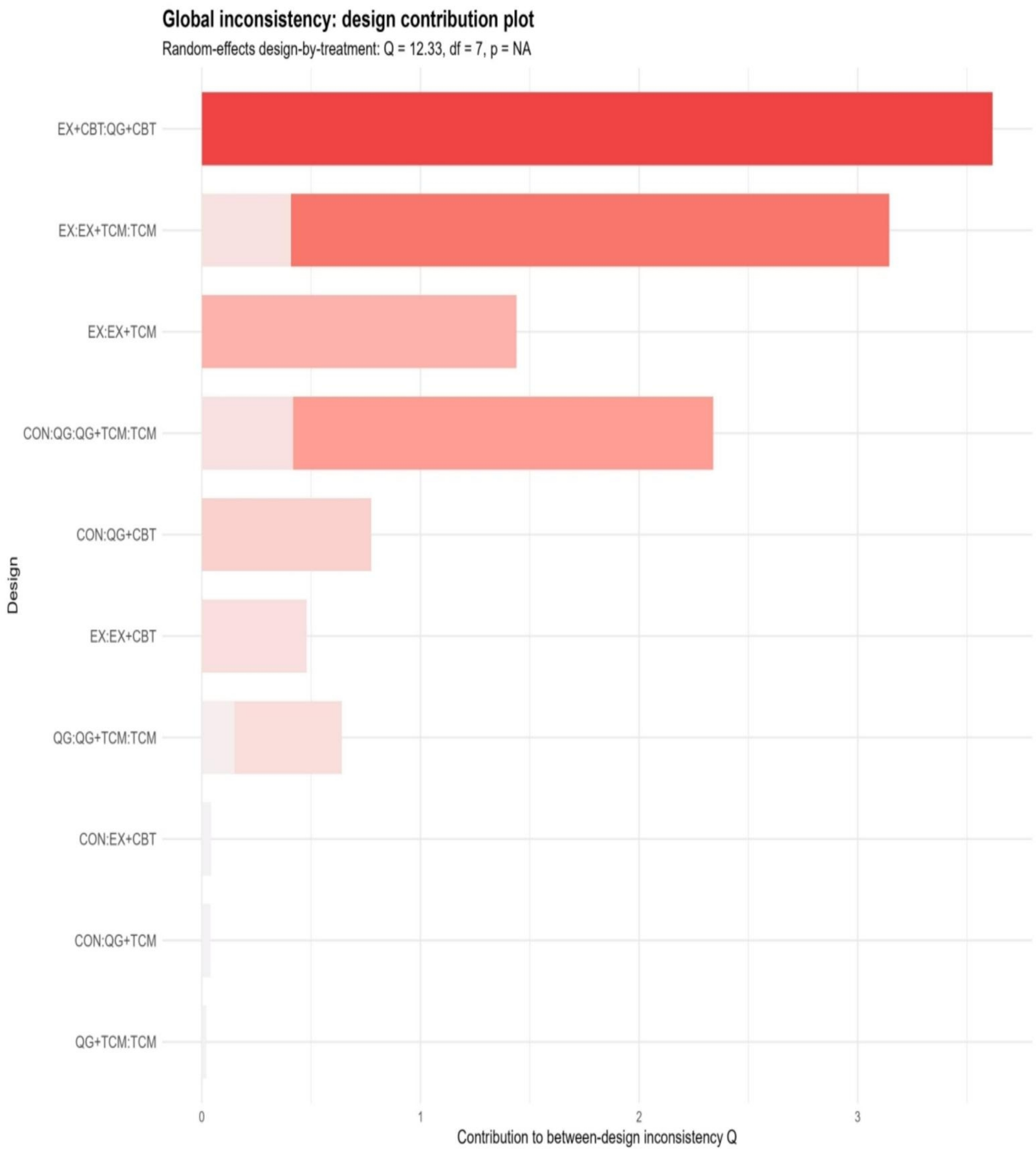

Supplementary Figure 5. Design contribution plot for inconsistency assessment

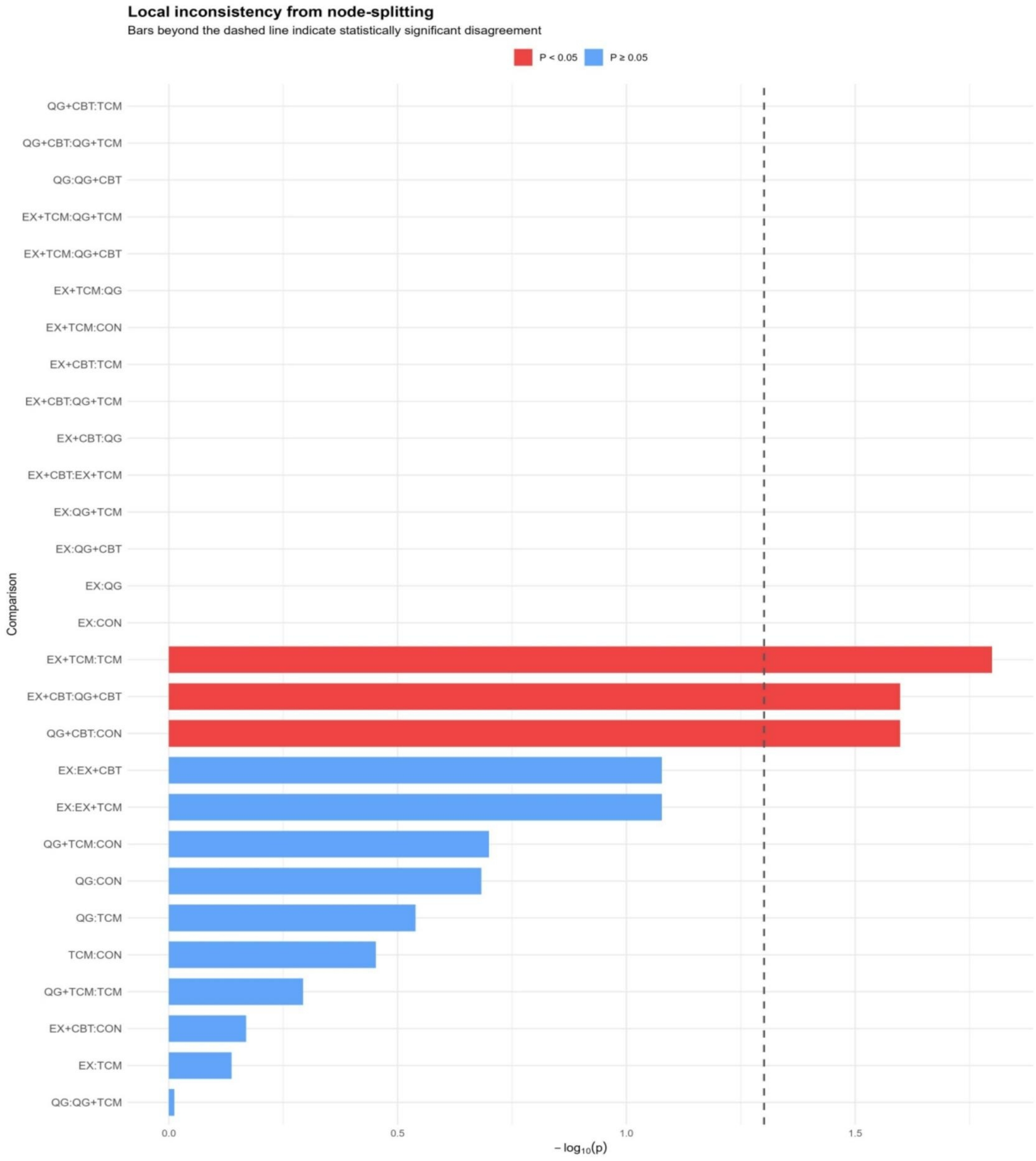

Supplementary Figure 6. Node-splitting p-value plot for inconsistency assessment in the PSQI network

Comparison	Number of Studies	Direct Evidence	I2	MD	95%-CI
**EX+CBT:CON**					
Direct estimate	5	0.76	93.4%	−2.96	[−4.51; −1.42]
Indirect estimate				−2.30	[−5.04; 0.45]
Node−splitting / SIDE forest plot | Network estimate				−2.80	[−4.15; −1.45]
**QG:CON**					
Direct estimate	1	0.32		−1.45	[−4.70; 1.80]
Indirect estimate				1.10	[−1.16; 3.35]
Node−splitting / SIDE forest plot | Network estimate				0.27	[−1.58; 2.12]
**QG+CBT:CON**					
Direct estimate	4	0.85	82.3%	−2.00	[−3.62; −0.39]
Indirect estimate				−6.69	[−10.47; −2.92]
Node−splitting / SIDE forest plot | Network estimate				−2.73	[−4.21; −1.24]
**QG+TCM:CON**					
Direct estimate	4	0.82	98.0%	−2.62	[−4.19; −1.06]
Indirect estimate				−0.23	[−3.54; 3.08]
Node−splitting / SIDE forest plot | Network estimate				−2.19	[−3.60; −0.77]
**TCM:CON**					
Direct estimate	1	0.23		−1.98	[−5.30; 1.34]
Indirect estimate				−0.18	[−2.01; 1.64]
Node−splitting / SIDE forest plot | Network estimate				−0.60	[−2.20; 1.00]
**EX:EX+CBT**					
Direct estimate	4	0.84	82.6%	2.51	[0.83; 4.19]
Indirect estimate				−1.18	[−5.02; 2.65]
Node−splitting / SIDE forest plot | Network estimate				1.92	[0.38; 3.45]
**EX:EX+TCM**					
Direct estimate	4	0.95	74.4%	1.43	[−0.32; 3.19]
Indirect estimate				8.07	[0.76; 15.39]
Node−splitting / SIDE forest plot | Network estimate				1.79	[0.09; 3.50]
**EX:TCM**					
Direct estimate	1	0.35		−0.79	[−4.32; 2.74]
Indirect estimate				−0.02	[−2.60; 2.56]
Node−splitting / SIDE forest plot | Network estimate				−0.29	[−2.37; 1.80]
**EX+CBT:QG+CBT**					
Direct estimate	1	0.28		3.31	[−0.18; 6.80]
Indirect estimate				−1.38	[−3.54; 0.79]
Node−splitting / SIDE forest plot | Network estimate				−0.07	[−1.91; 1.76]
**EX+TCM:TCM**					
Direct estimate	1	0.51		−4.93	[−8.25; −1.61]
Indirect estimate				0.94	[−2.48; 4.37]
Node−splitting / SIDE forest plot | Network estimate				−2.08	[−4.47; 0.30]
**QG:QG+TCM**					
Direct estimate	4	0.85	59.9%	2.47	[0.91; 4.03]
Indirect estimate				2.40	[−1.27; 6.06]
Node−splitting / SIDE forest plot | Network estimate				2.46	[1.02; 3.89]
**QG:TCM**					
Direct estimate	4	0.86	76.7%	1.19	[−0.38; 2.76]
Indirect estimate				−1.09	[−4.98; 2.81]
Node−splitting / SIDE forest plot | Network estimate				0.87	[−0.59; 2.32]
**QG+TCM:TCM**					
Direct estimate	8	0.92	79.5%	−1.48	[−2.61; −0.35]
Indirect estimate				−2.85	[−6.74; 1.05]
Node−splitting / SIDE forest plot | Network estimate				−1.59	[−2.67; −0.50]

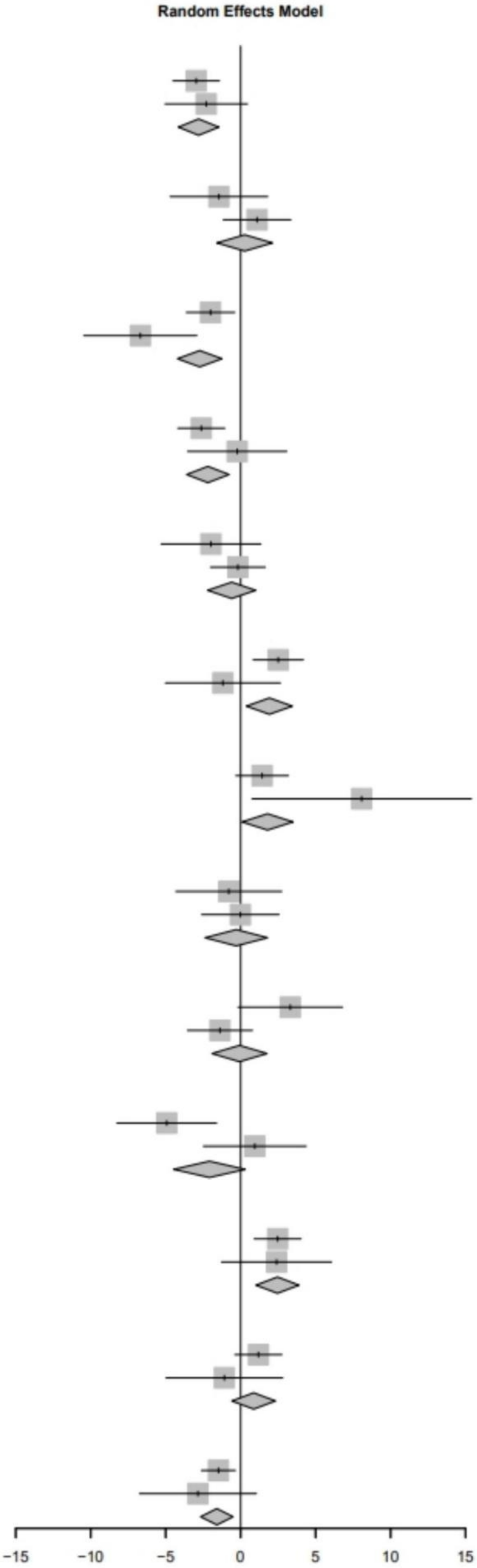

Supplementary Figure 7. Forest plot of node-splitting results in the PSQI network

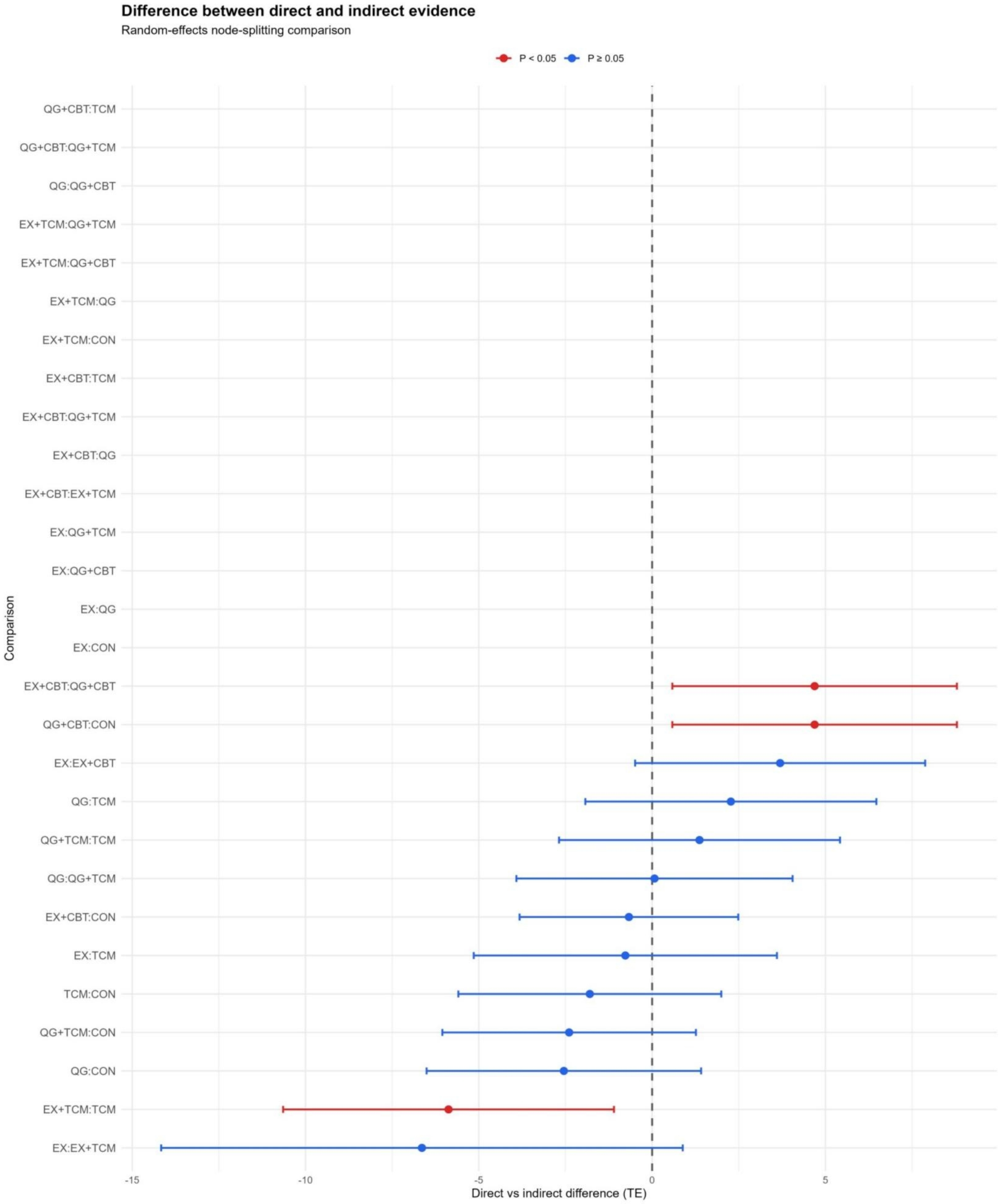

Supplementary Figure 8. Direct–indirect comparison forest plot for the PSQI network

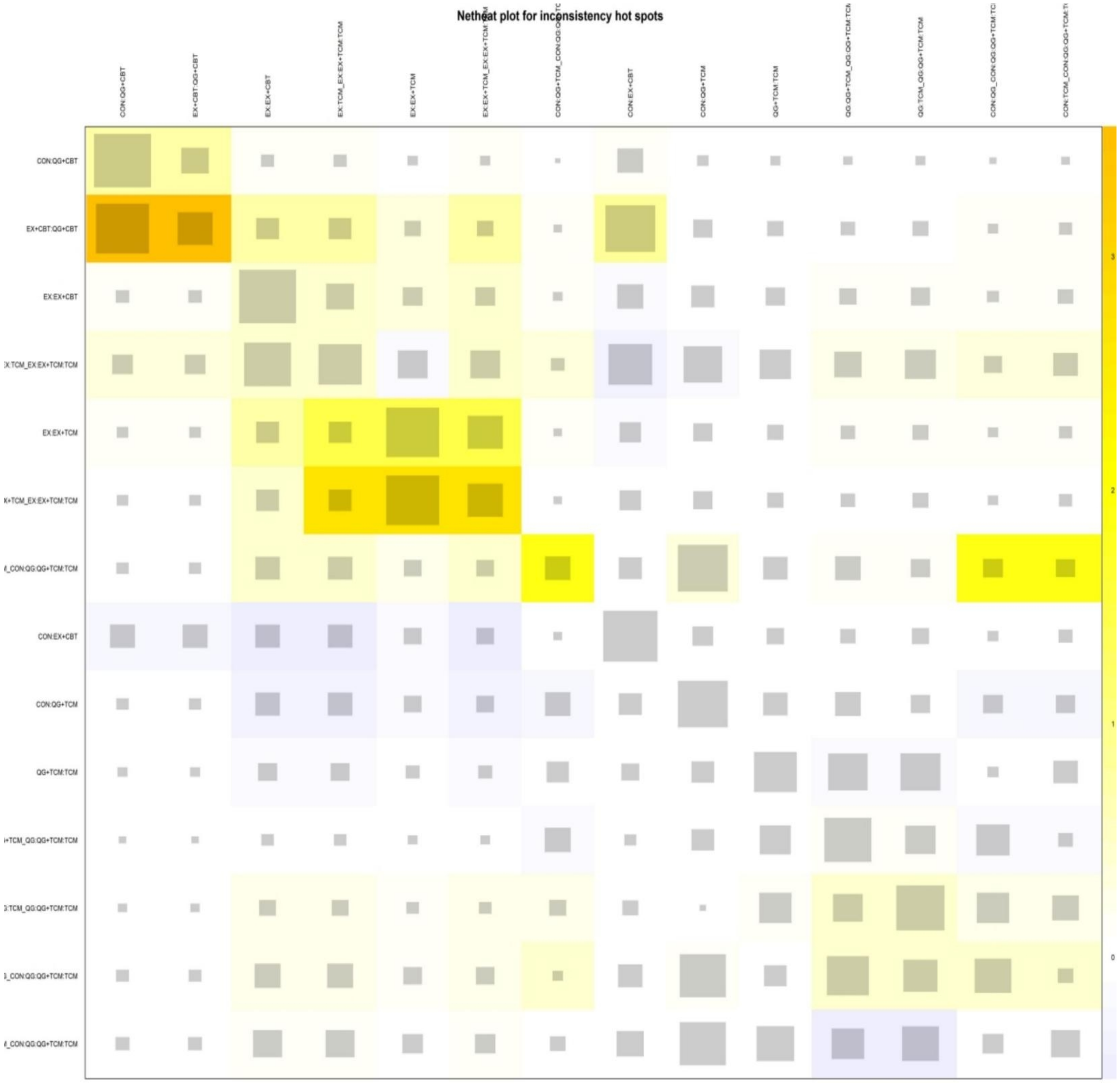

Supplementary Figure 9. Net heat plot for inconsistency assessment in the PSQI network

Supplementary Figures 10–13：PSQI forest plots

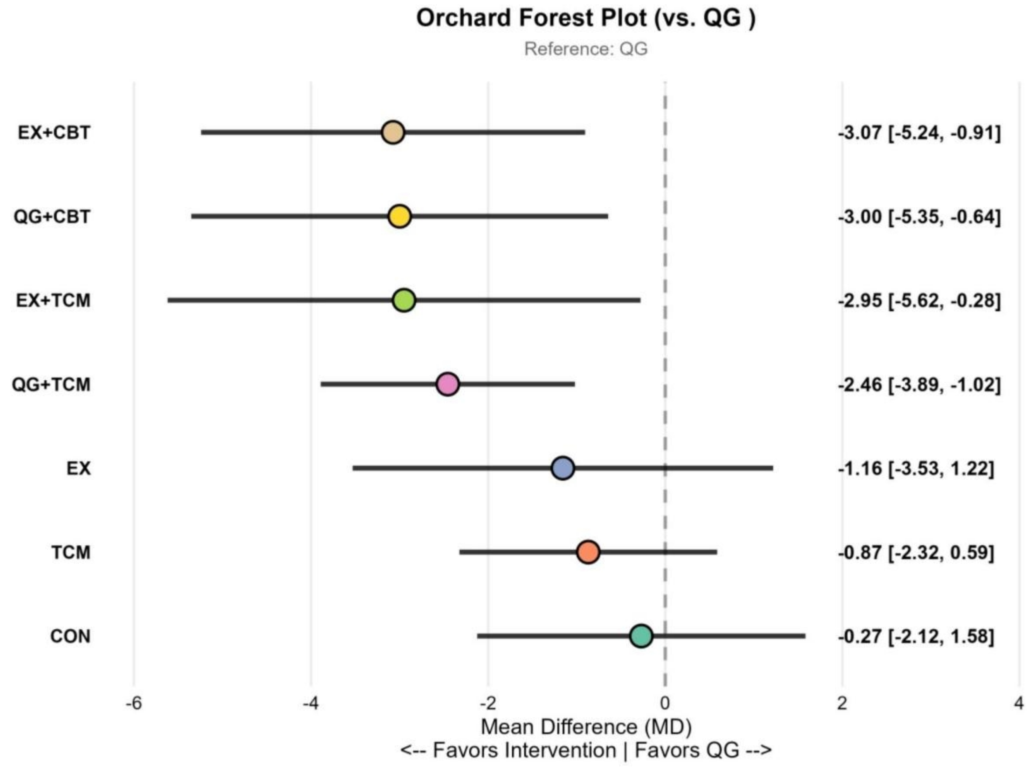

Supplementary Figure 10. Forest plot of network meta-analysis effect estimates versus QG for PSQI

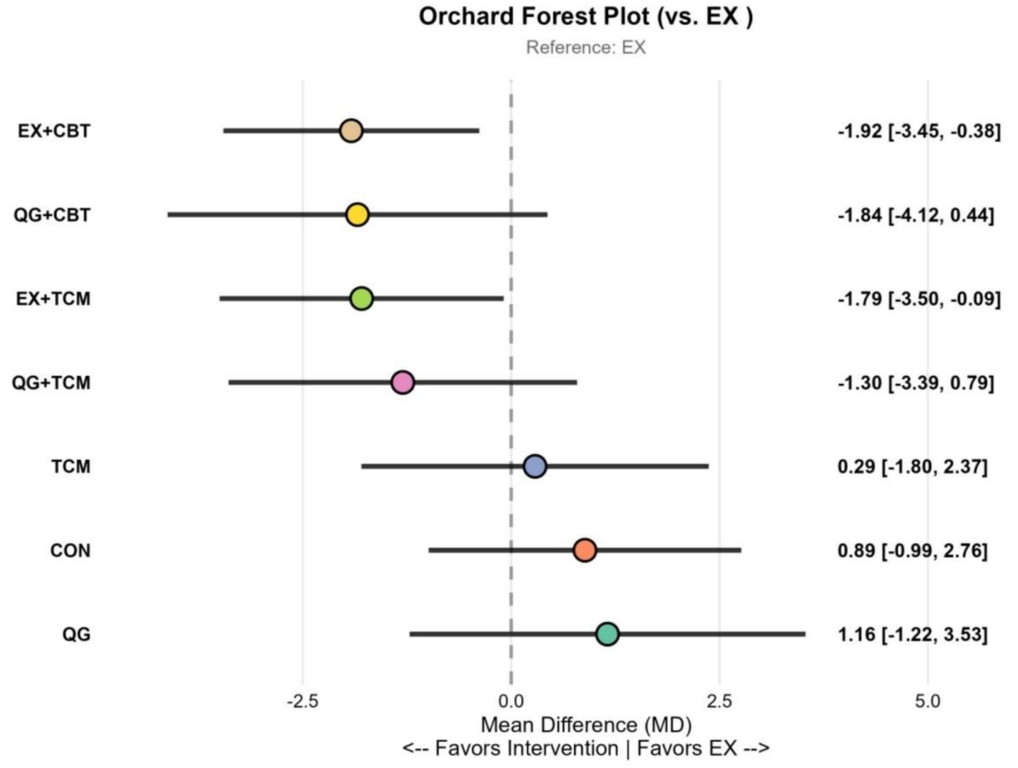

Supplementary Figure 11. Forest plot of network meta-analysis effect estimates versus EX for PSQI

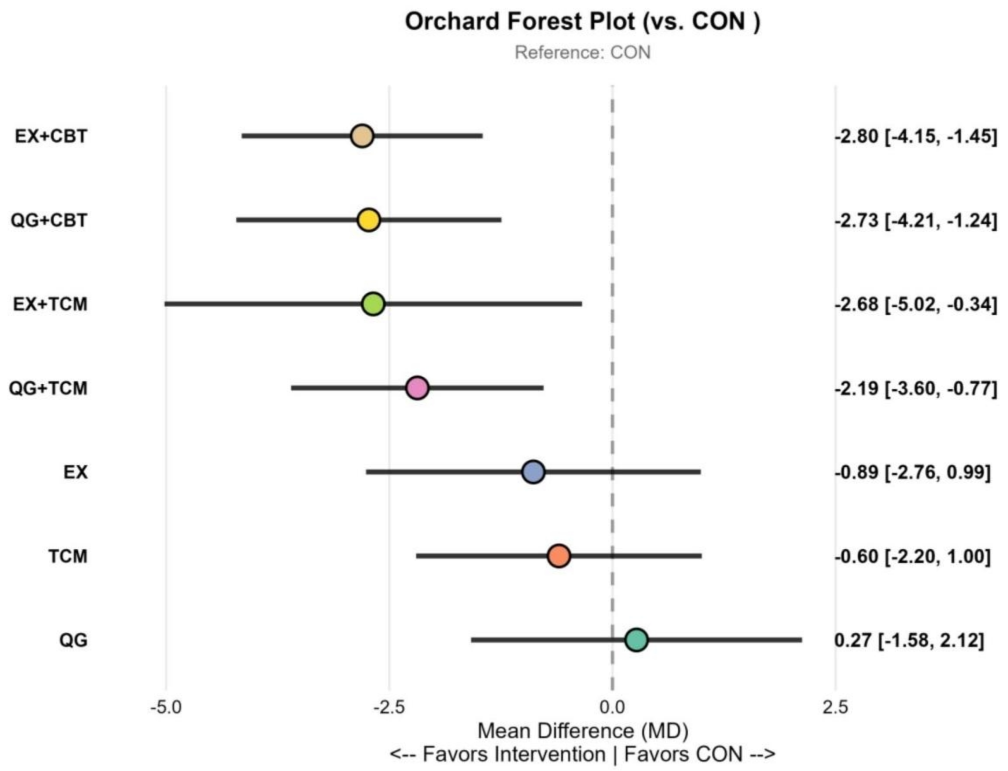

Supplementary Figure 12. Forest plot of network meta-analysis effect estimates versus CON for PSQI

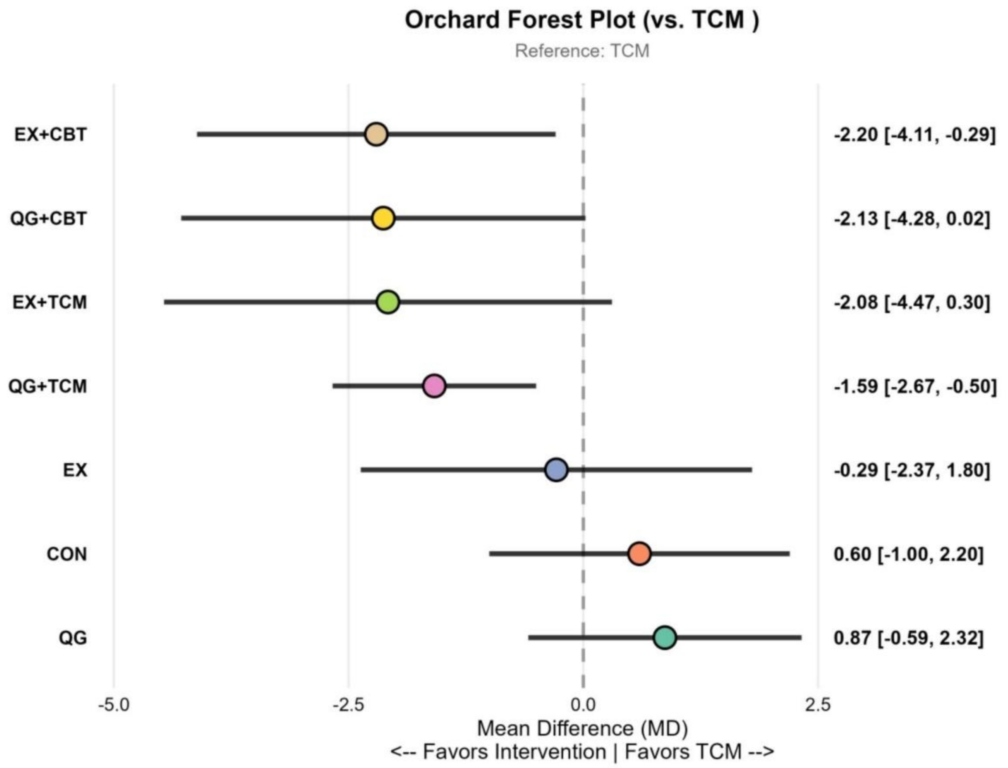

Supplementary Figure 13. Forest plot of network meta-analysis effect estimates versus TCM for PSQI

Supplementary Figures 14–15: TST forest plots

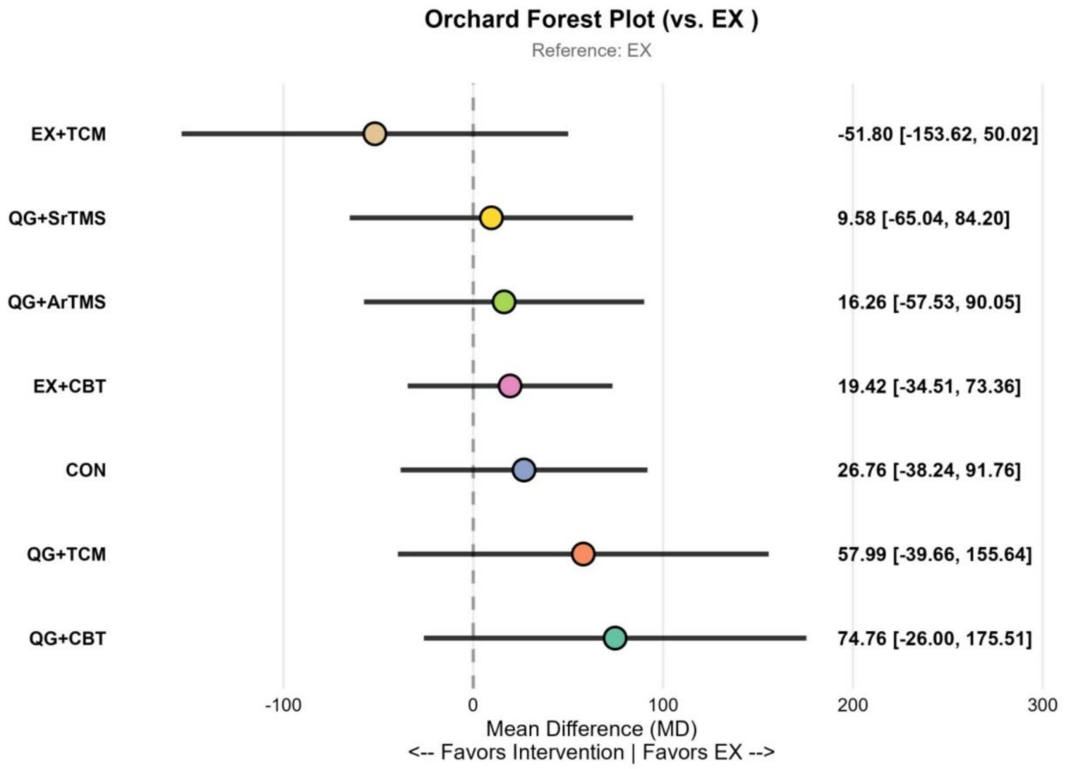

Supplementary Figure 14. Forest plot of network meta-analysis effect estimates versus EX for TST

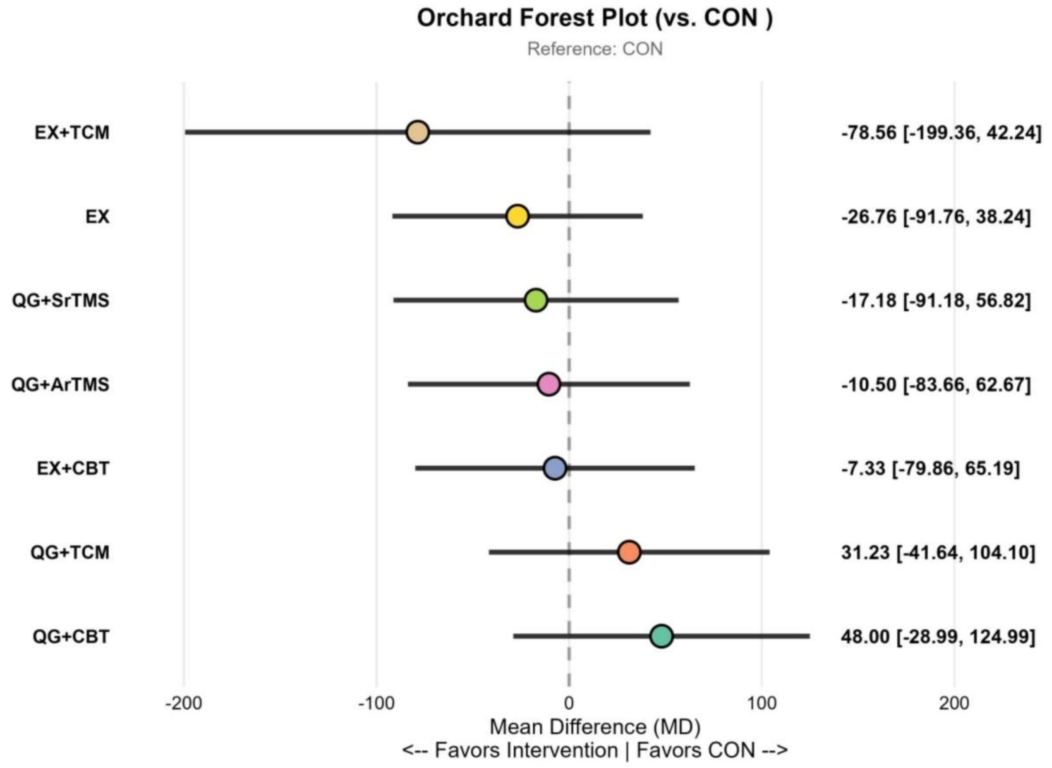

Supplementary Figure 15. Forest plot of network meta-analysis effect estimates versus CON for TST

Supplementary Figures 16–17：SOL forest plots

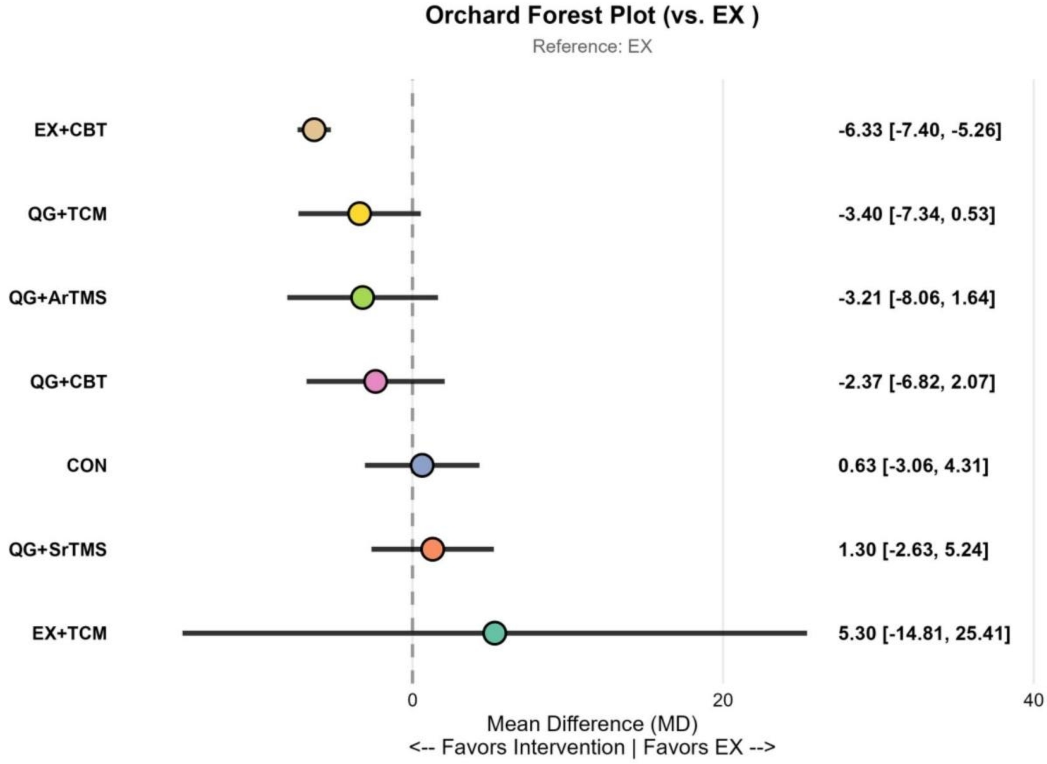

Supplementary Figure 16. Forest plot of network meta-analysis effect estimates versus EX for SOL

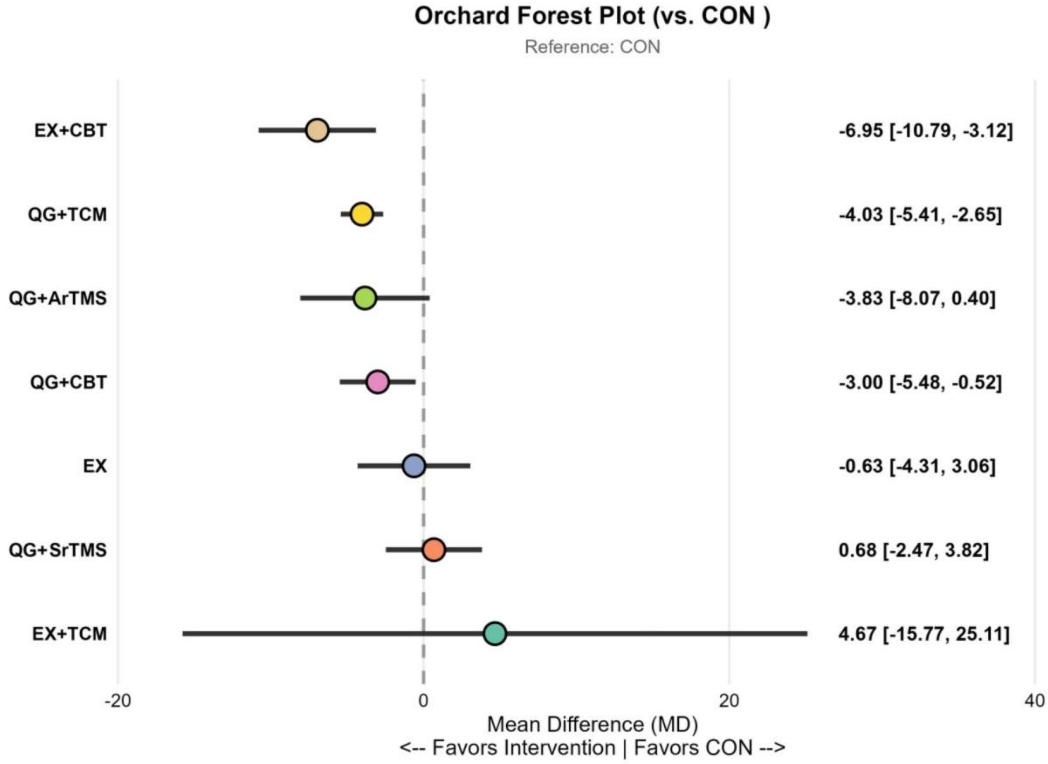

Supplementary Figure 17. Forest plot of network meta-analysis effect estimates versus CON for SOL

Supplementary Figures 18–19： WASO forest plots

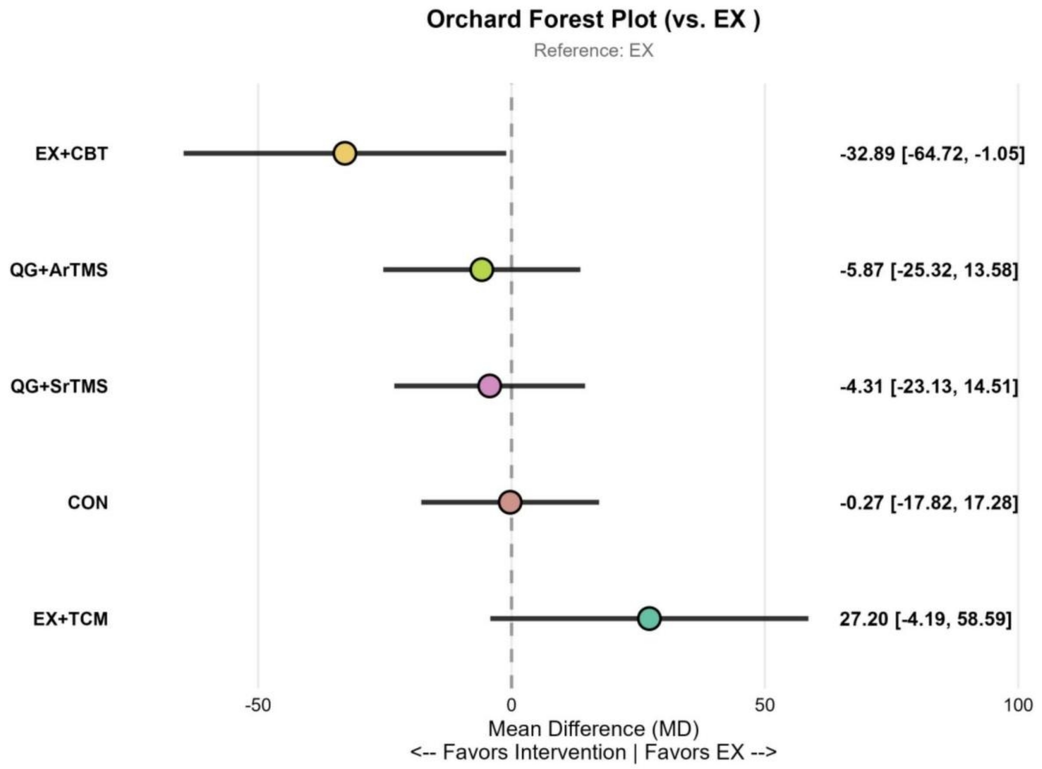

Supplementary Figure 18. Forest plot of network meta-analysis effect estimates versus EX for WASO

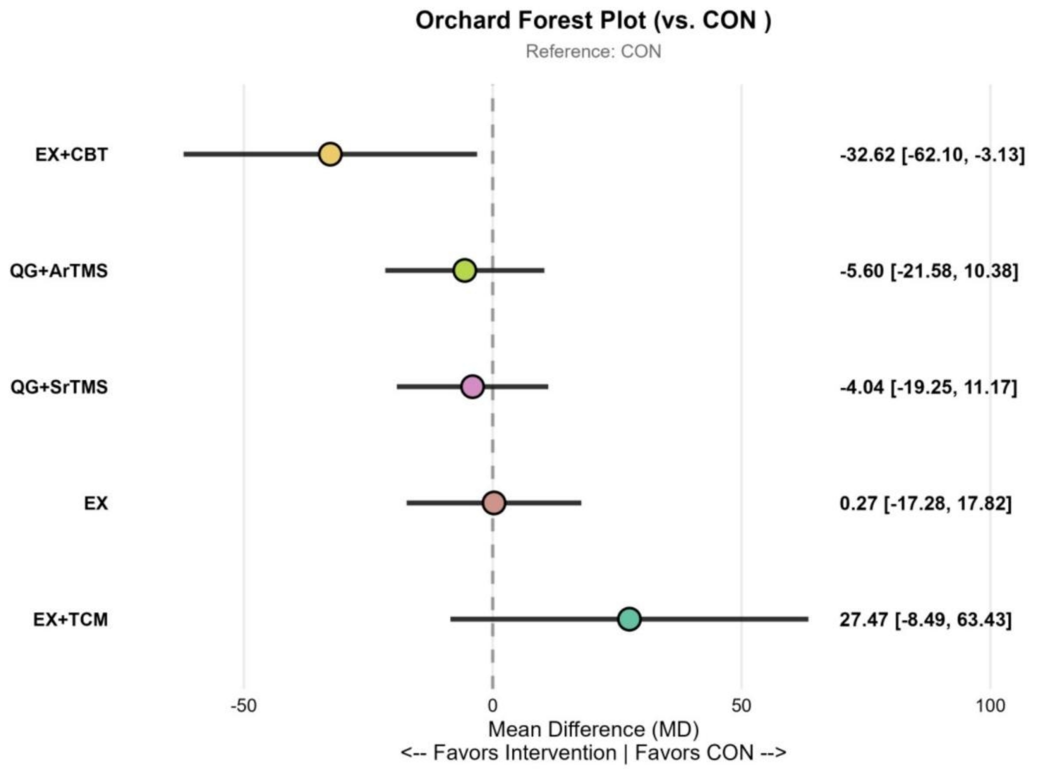

Supplementary Figure 19. Forest plot of network meta-analysis effect estimates versus CON for WASO

Supplementary Figures 20–23. SUCRA ranking plots

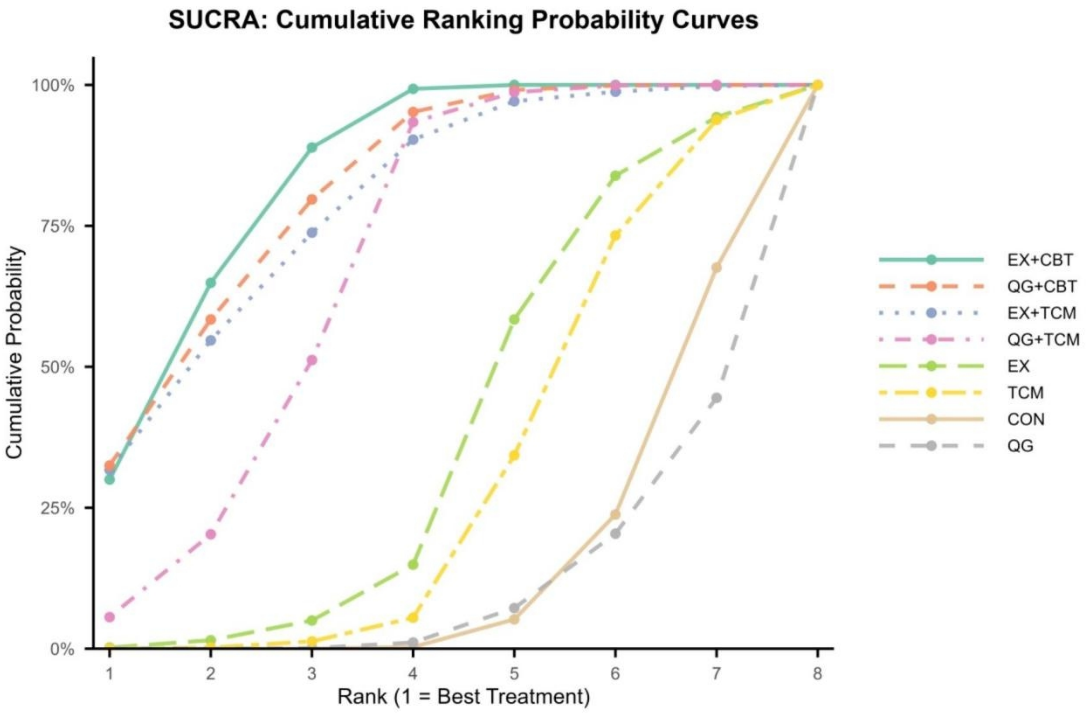

Supplementary Figure 20. SUCRA ranking plot for PSQI

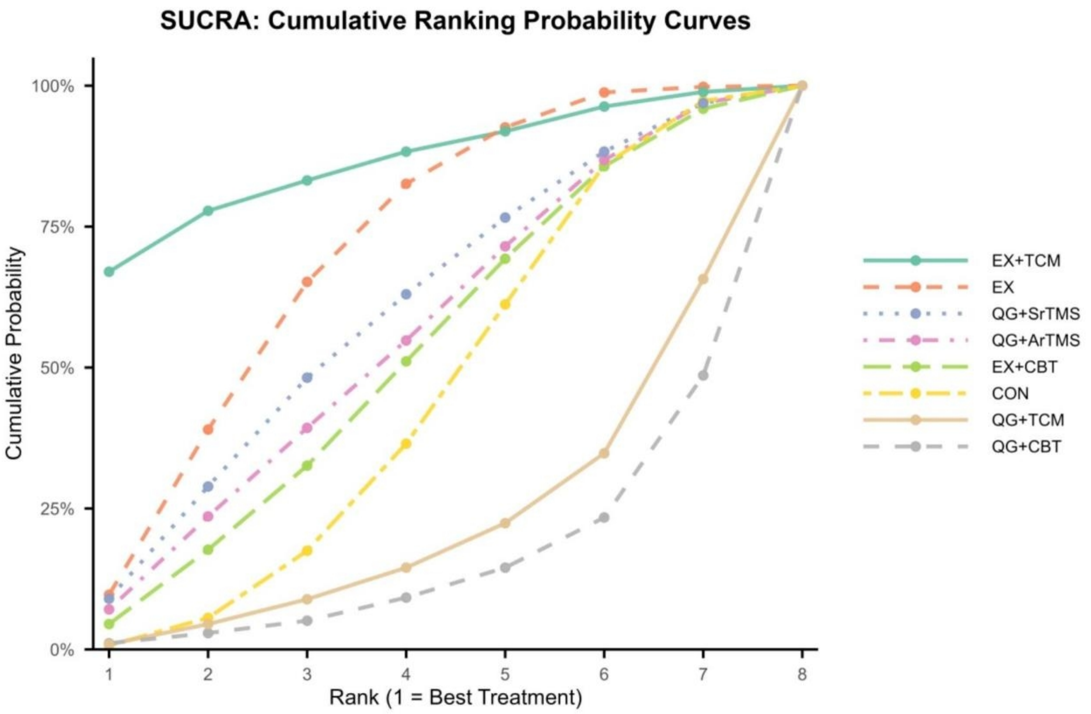

Supplementary Figure 21. SUCRA ranking plot for TST

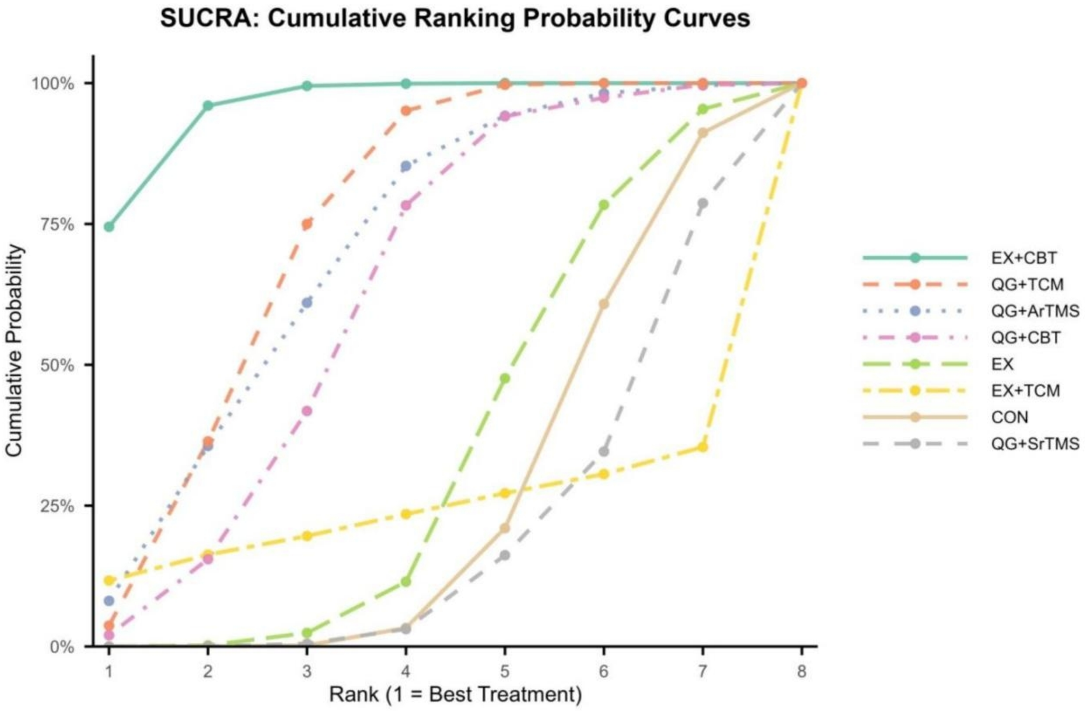

Supplementary Figure 22. SUCRA ranking plot for SOL

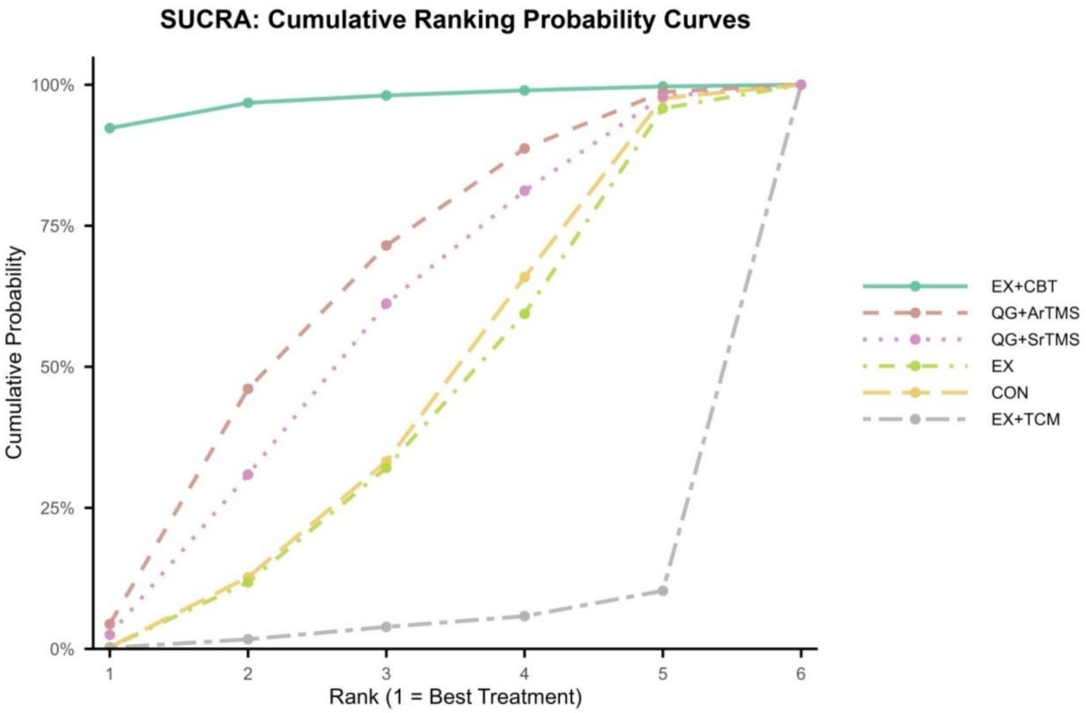

Supplementary Figure 23. SUCRA ranking plot for WASO

Supplementary Figures 24–27. League heatmaps

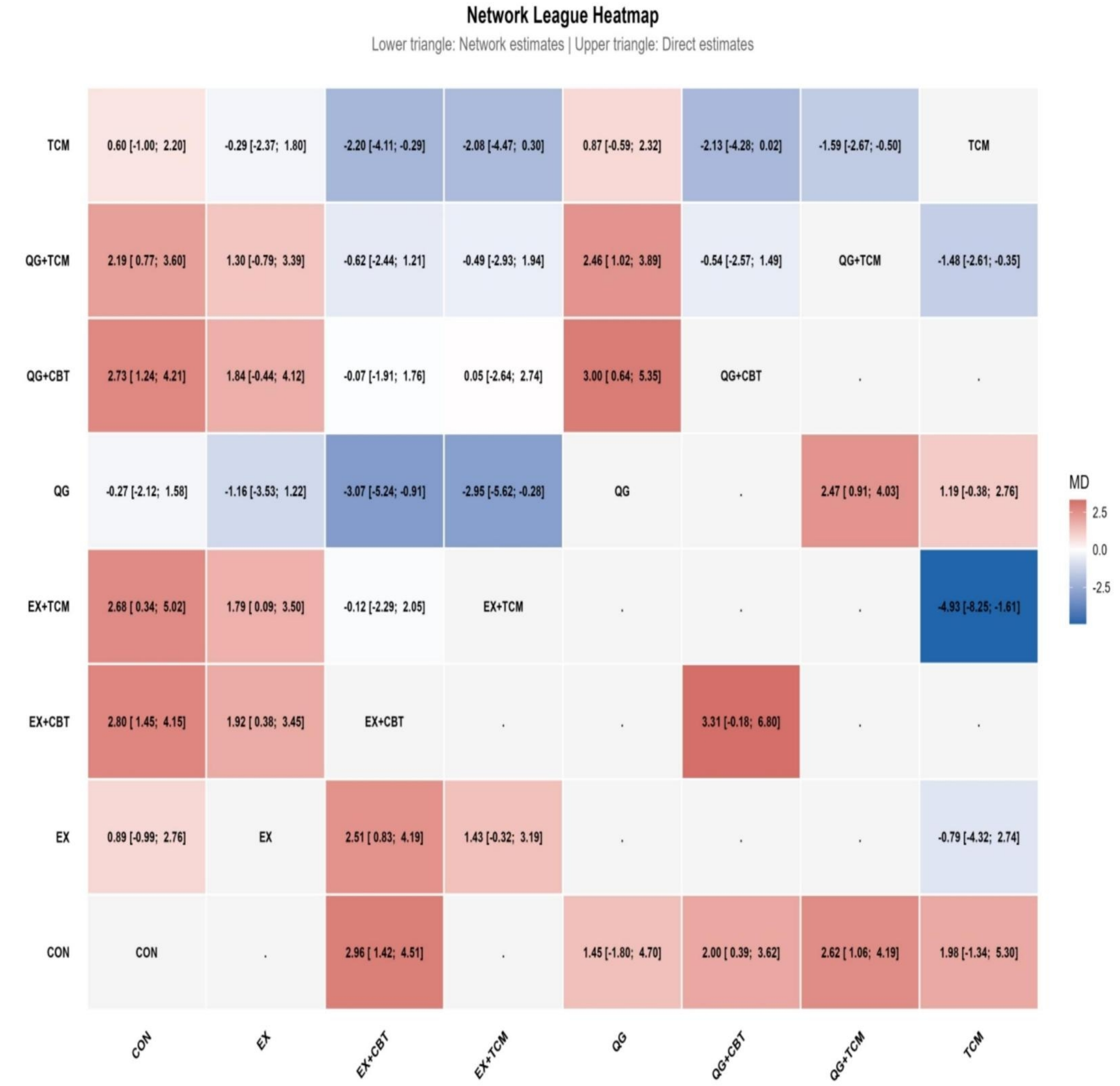

Supplementary Figure 24. League heatmap for PSQI

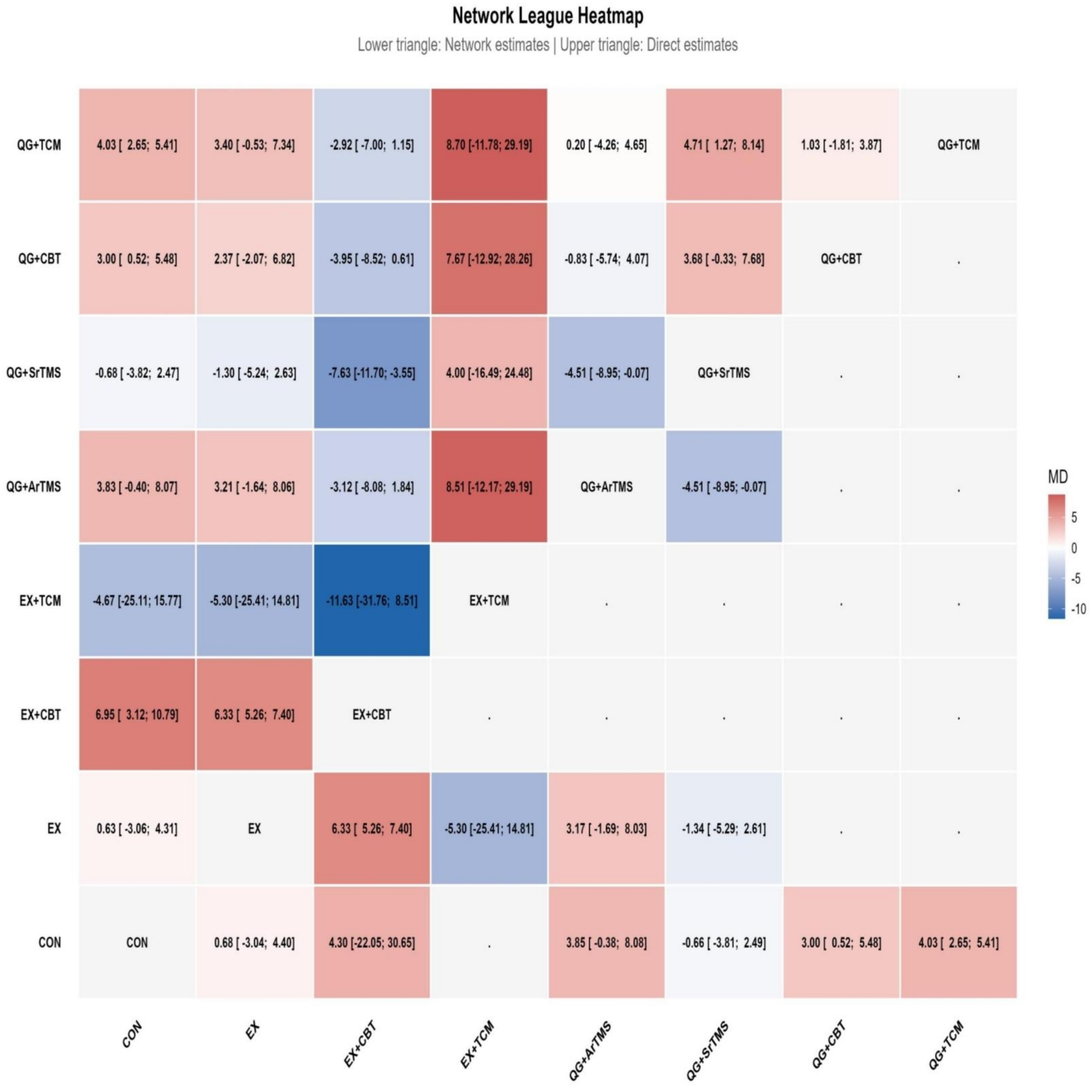

Supplementary Figure 25. League heatmap for SOL

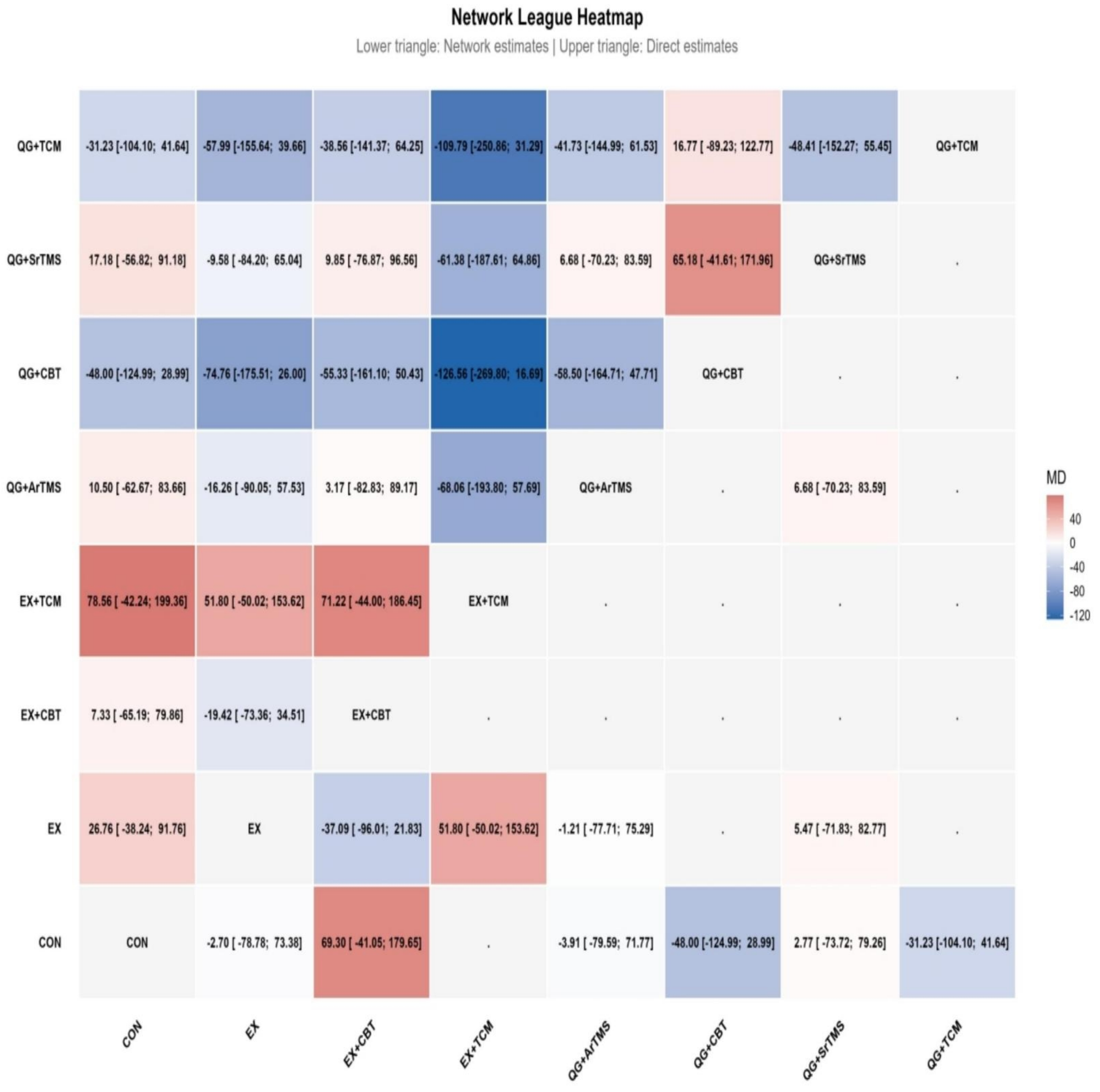

Supplementary Figure 26. League heatmap for TST

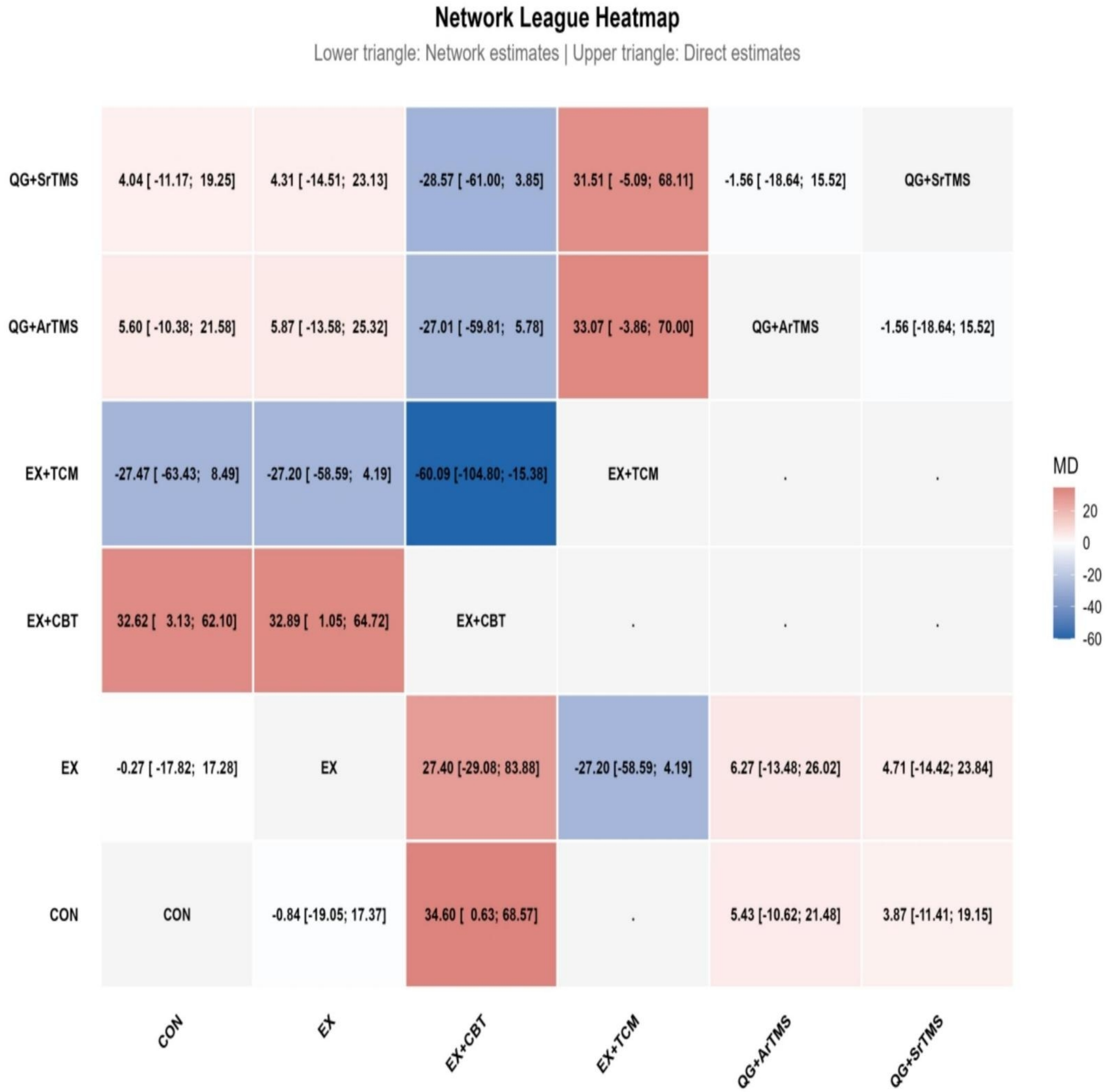

Supplementary Figure 27. League heatmap for WASO

Supplementary Figures 28–33：Network meta-regression bubble plots

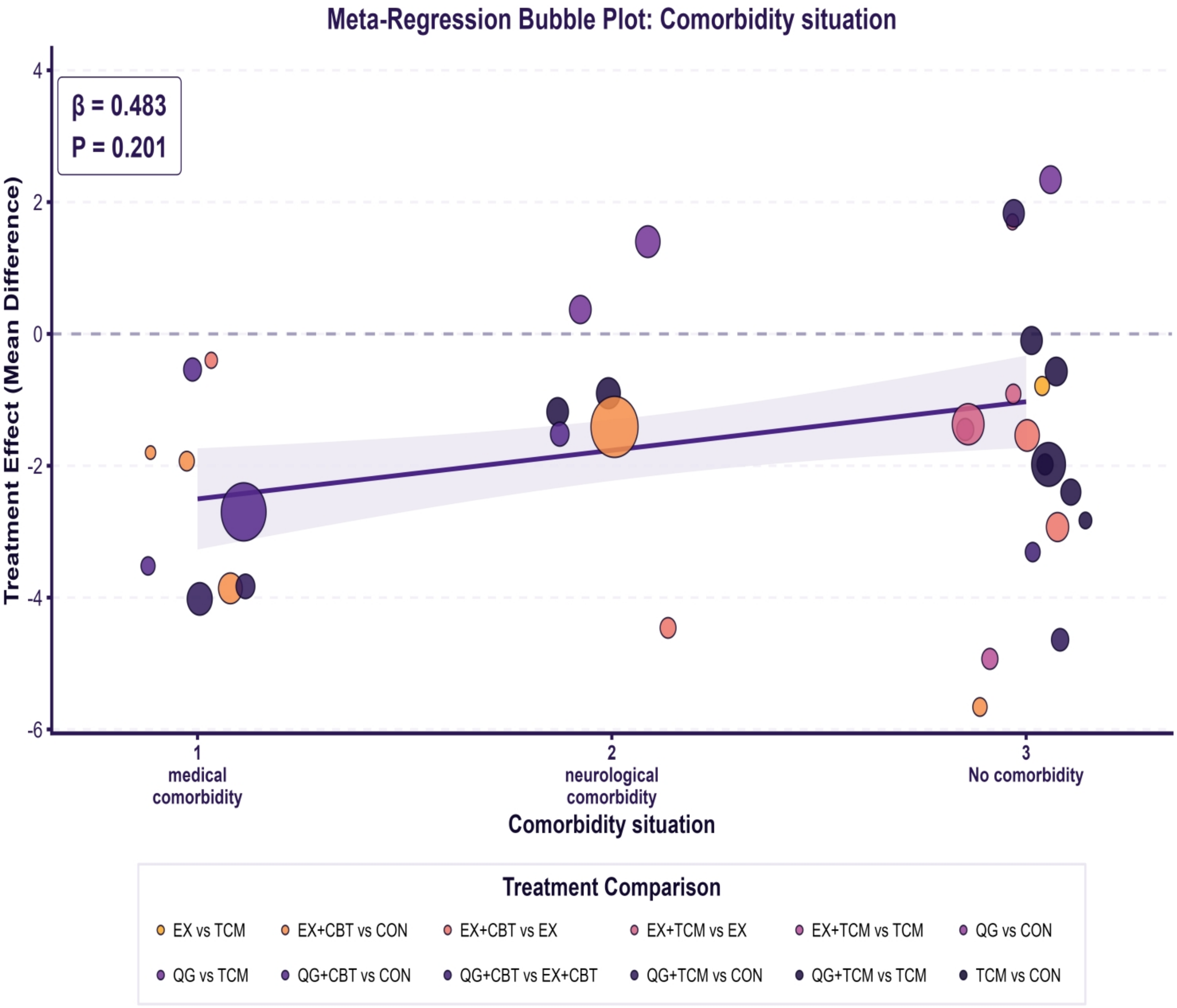

Supplementary Figure 28. Network meta-regression bubble plot for comorbidity status

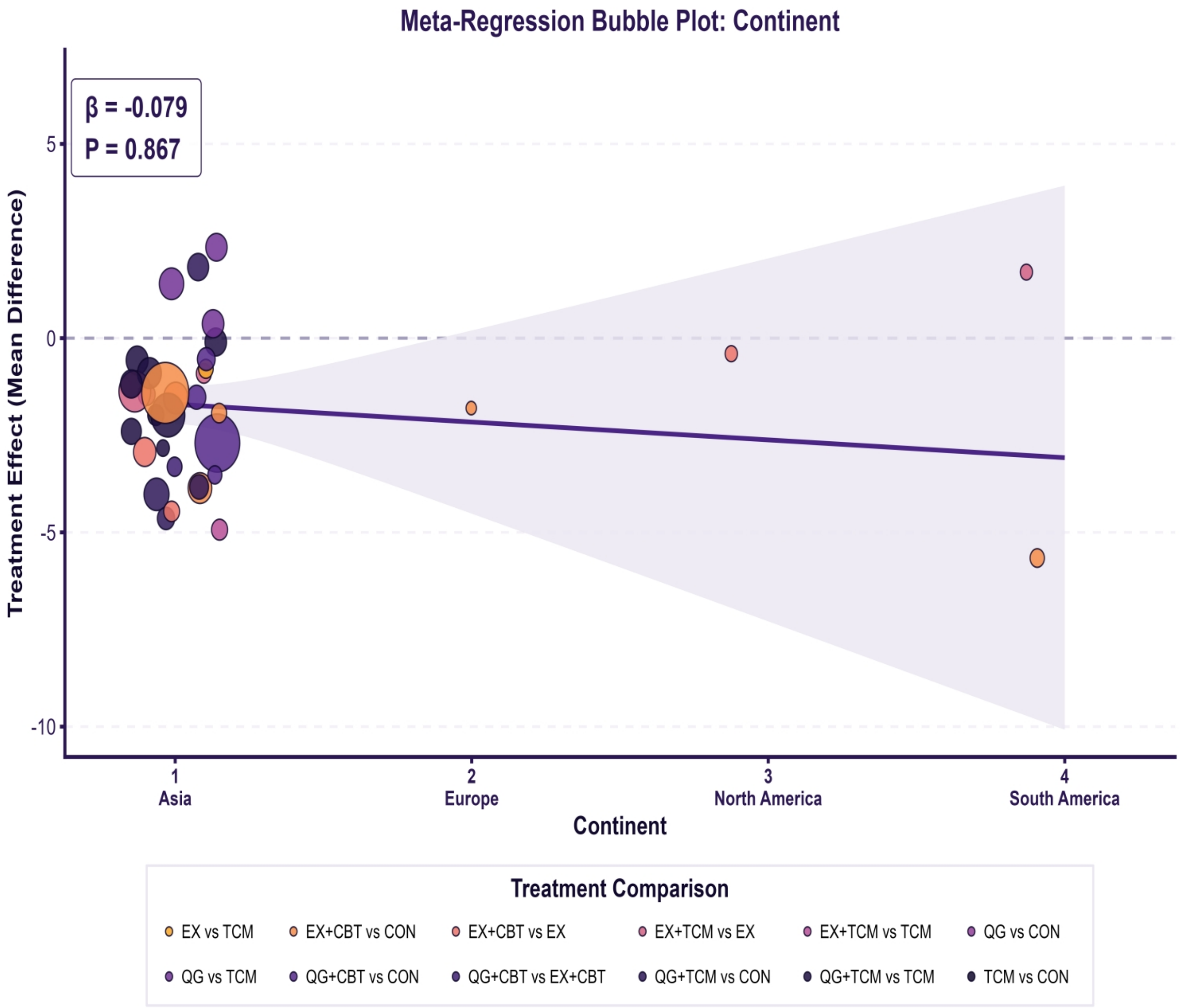

Supplementary Figure 29. Network meta-regression bubble plot for continent

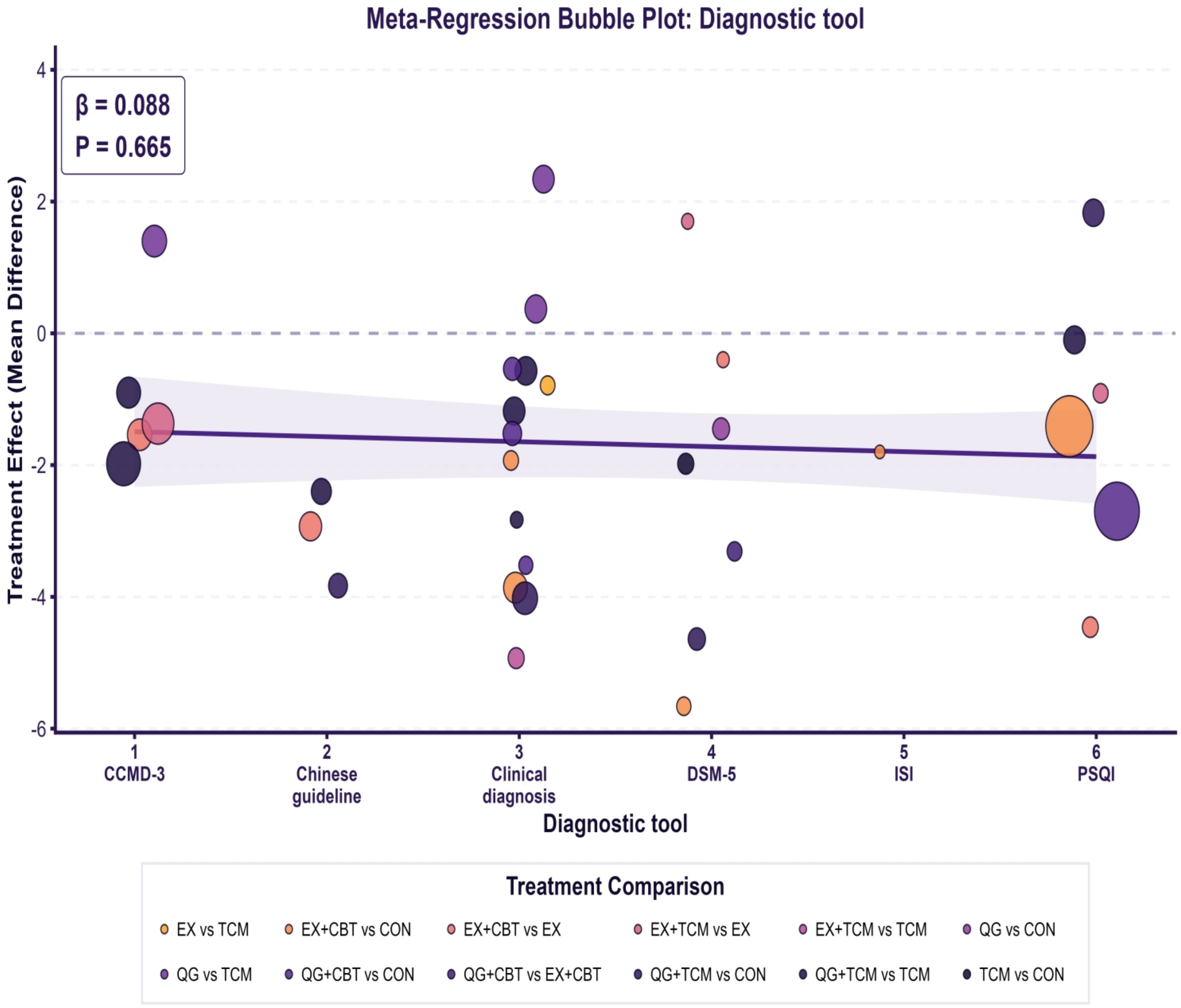

Supplementary Figure 30. Network meta-regression bubble plot for diagnostic tool

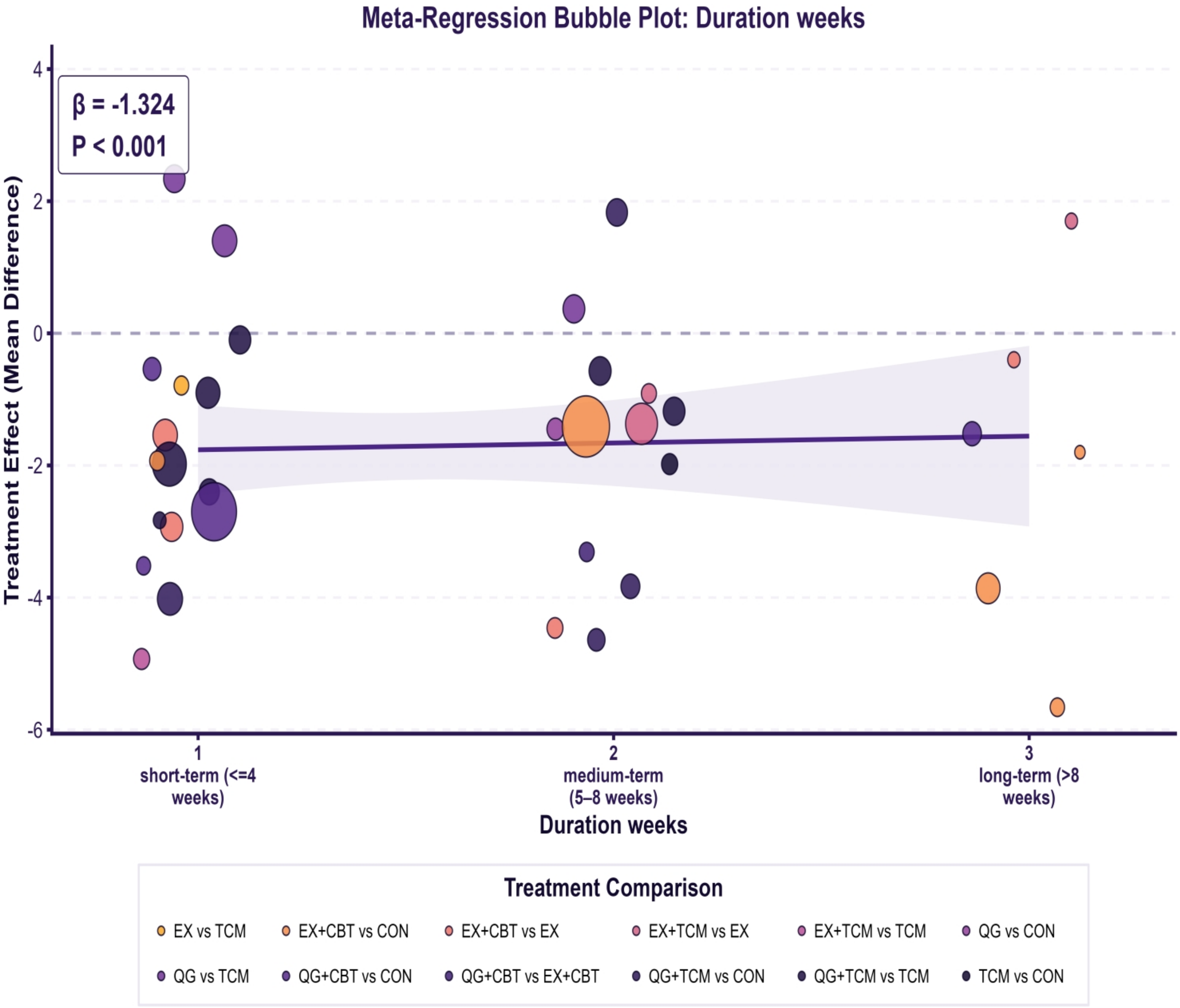

Supplementary Figure 31. Network meta-regression bubble plot for duration weeks

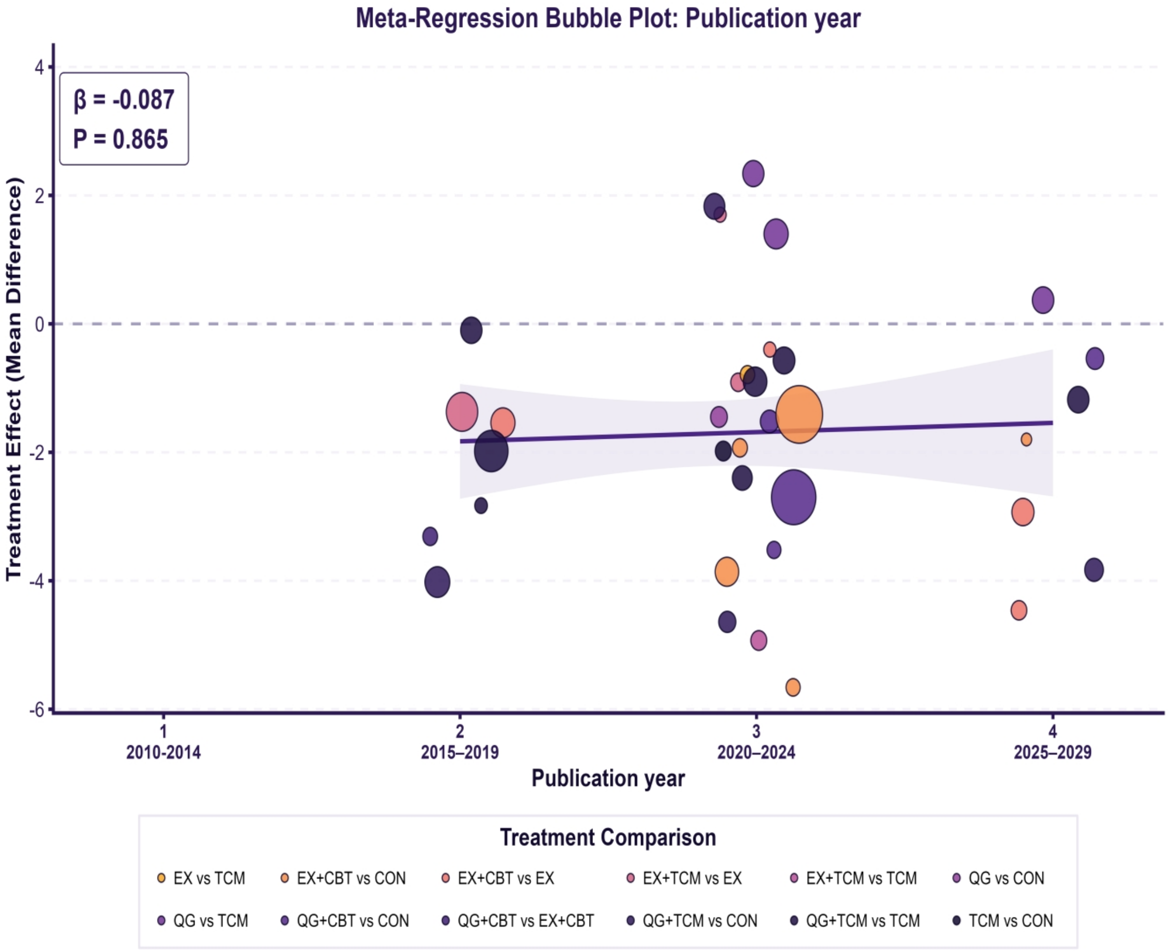

Supplementary Figure 32. Network meta-regression bubble plot for publication year

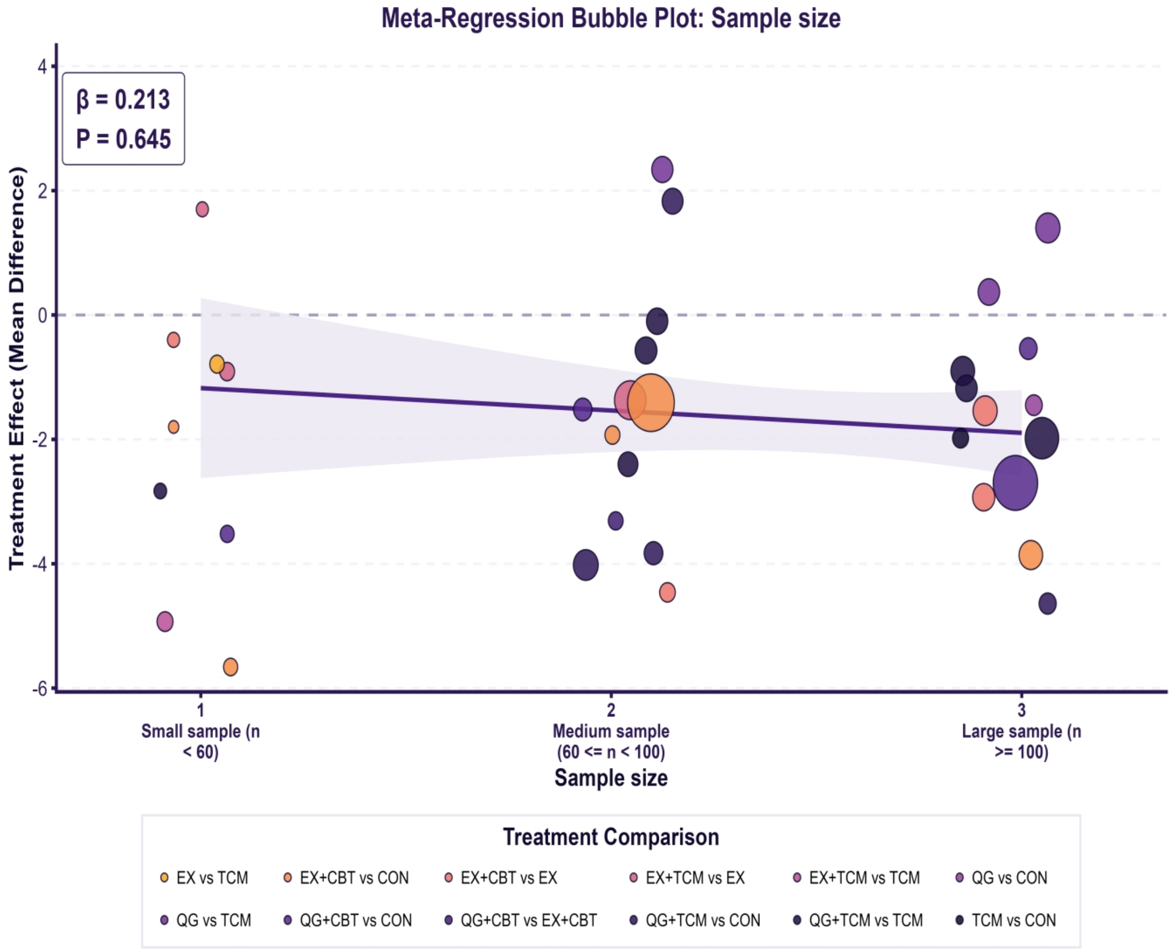

Supplementary Figure 33. Network meta-regression bubble plot for sample size

Supplementary Figures 34–37. Comparison-adjusted funnel plots

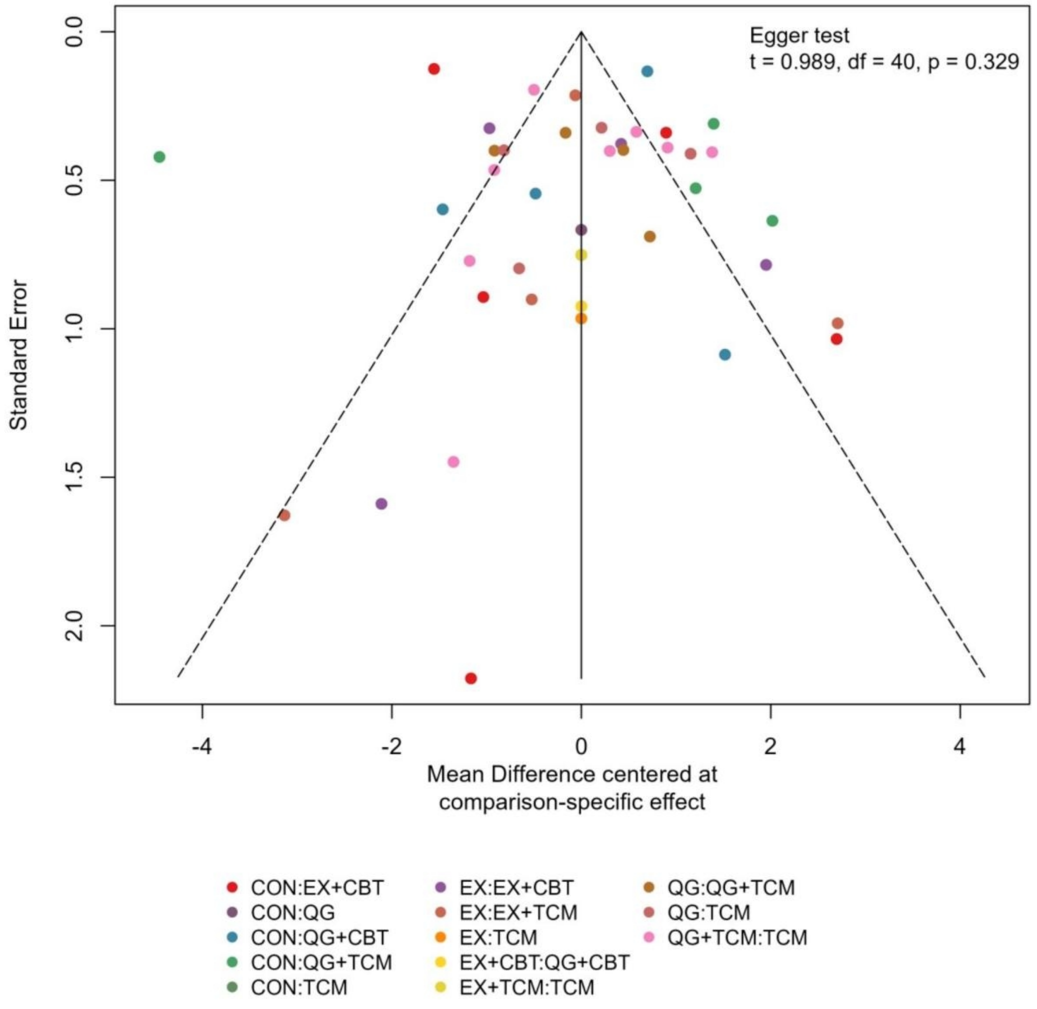

Supplementary Figure 34. Comparison-adjusted funnel plot for PSQI

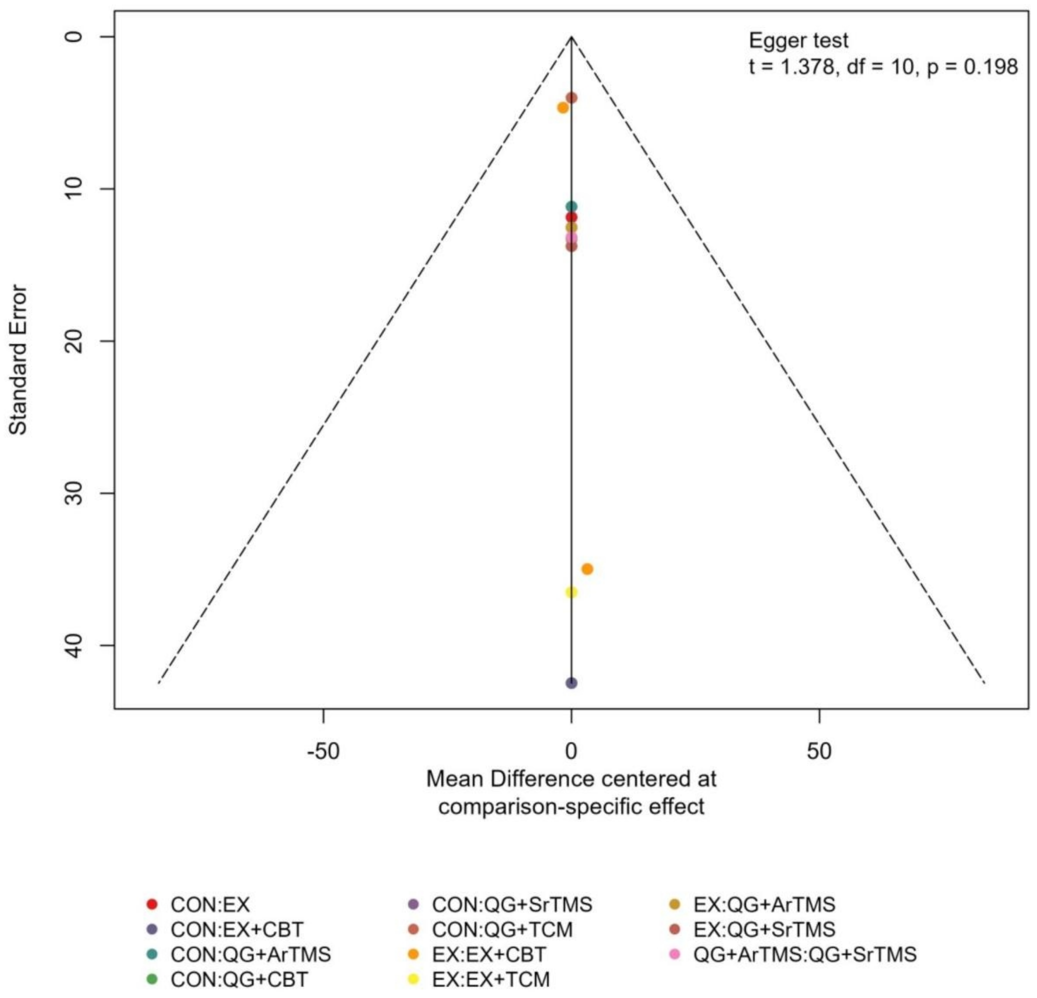

Supplementary Figure 35. Comparison-adjusted funnel plot for TST

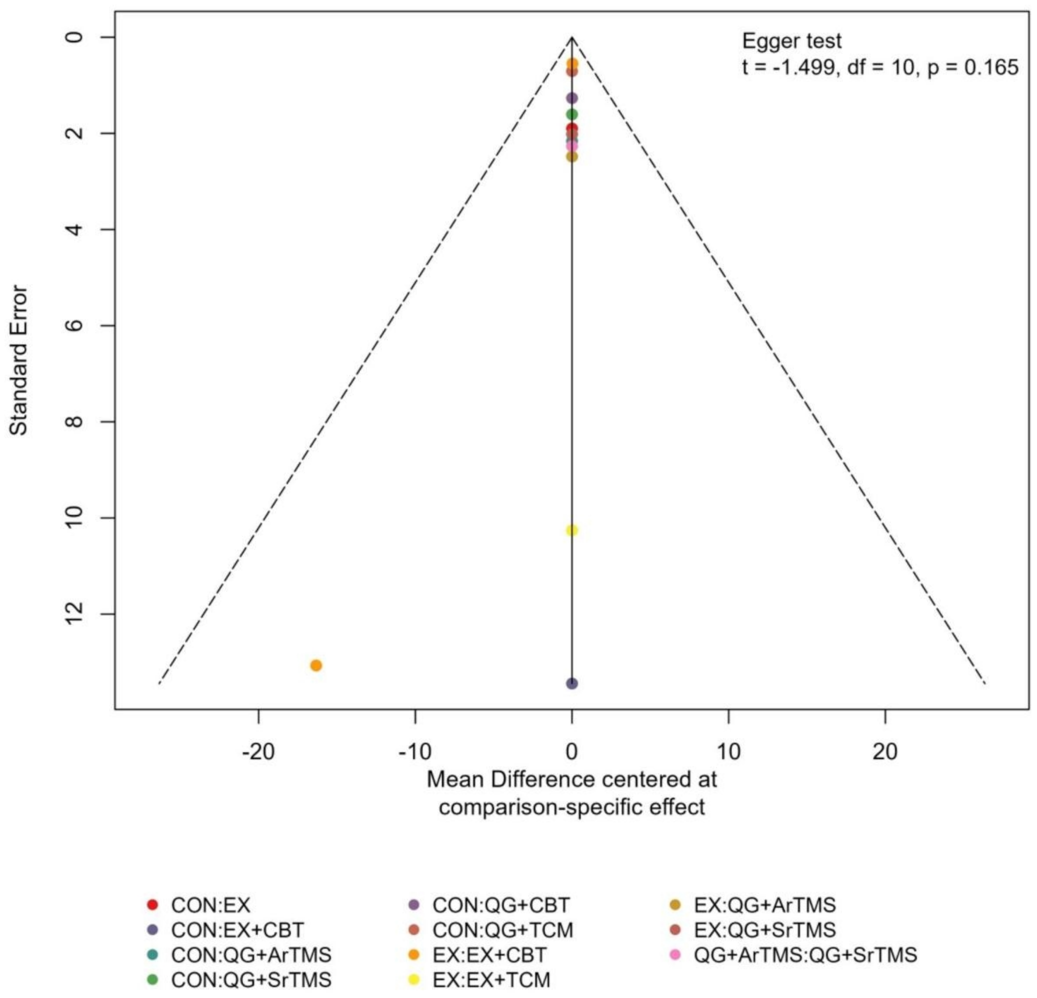

Supplementary Figure 36. Comparison-adjusted funnel plot for SOL

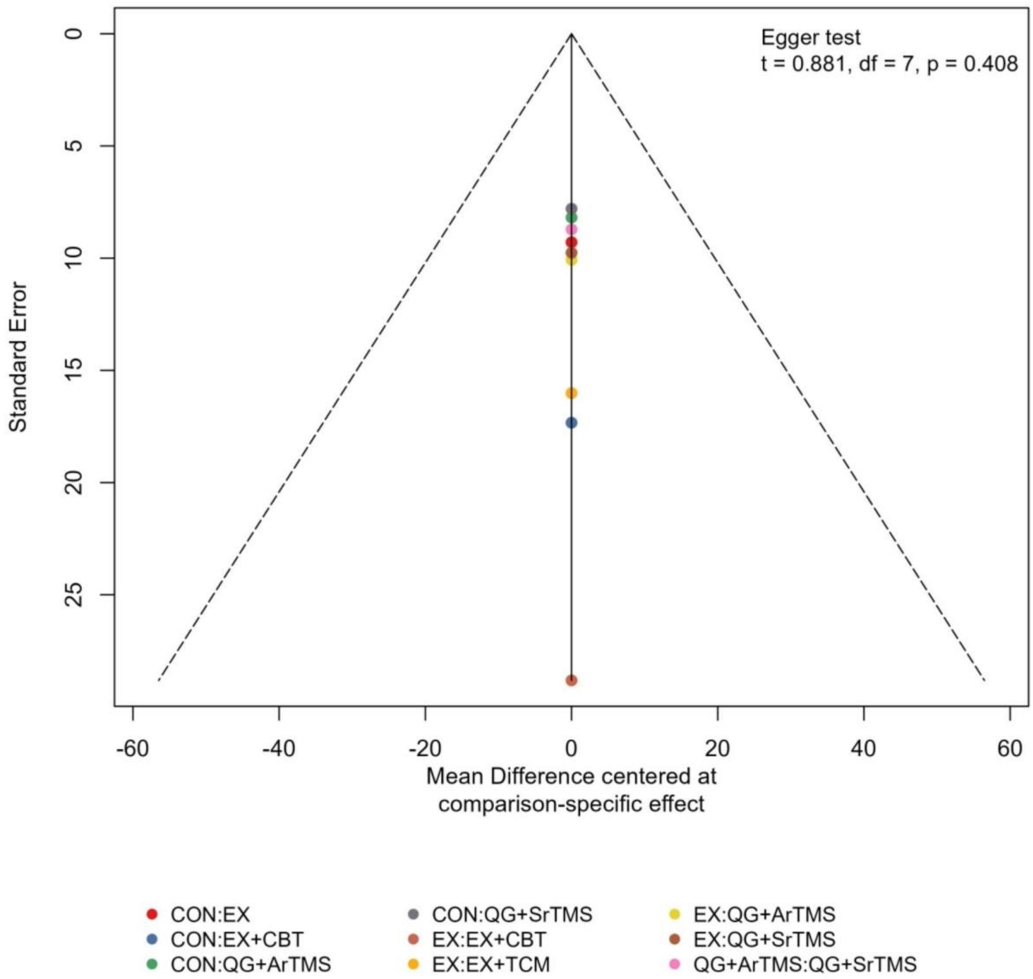

Supplementary Figure 37. Comparison-adjusted funnel plot for WASO
